# Supplementary material for: Evaluation of the effectiveness of potassium chloride in the management of out-of hospital cardiac arrest by refractory ventricular fibrillation: Study protocol of the POTACREH study
Source: PLoS One. 2023 Apr 12;18(4):e0284429. doi: 10.1371/journal.pone.0284429 (PMC10096226; doi:10.1371/journal.pone.0284429)
Supplement: S1 File — (DOC) [file pone.0284429.s002.doc]

| « Evaluation de l’efficacité du chlorure de potassium  dans la prise en charge des arrêts cardiaques extra-hospitaliers  par fibrillation ventriculaire réfractaire»  POTACREH |
| --- |

PROTOCOLE DE RECHERCHE INTERVENTIONNELLE IMPLIQUANT LA PERSONNE HUMAINE PORTANT SUR UN MEDICAMENT A USAGE HUMAIN

Version N°1.1 du 18/12/2019

Code projet : APHP180577 / N° EUDRACT: 2019-002544-24

**Investigateur coordonnateur :** Pr Benoît VIVIEN

Service d’Anesthésie - Réanimation - SAMU

Hôpital Necker – Enfants malades

Tél : 01 44 49 23 67

Courriel : [benoit.vivien@aphp.fr](mailto:benoit.vivien@aphp.fr)

**Responsable scientifique :** Dr Romain JOUFFROY

Service d’Anesthésie - Réanimation - SAMU

Hôpital Necker – Enfants malades

Tél : 01 44 49 24 75

Courriel : [romain.jouffroy@aphp.fr](mailto:romain.jouffroy@aphp.fr)

**Promoteur :** Assistance Publique – Hôpitaux de Paris (AP-HP)

et par délégation : Direction de la Recherche Clinique et de l’Innovation (DRCI)

Hôpital Saint-Louis

1, avenue Claude Vellefaux

Référent projet DRCI-Siege : Sylvie PRIEUR

Tél : 01 44 84 17 92

Courriel : [sylvie.prieur@aphp.fr](mailto:sylvie.prieur@aphp.fr)

Référent vigilance DRCI-Siège : Céline LE GALLUDEC

Courriel : [celine.legalludec@aphp.fr](mailto:celine.legalludec@aphp.fr)

**Structure chargée**

**du suivi de la recherche :** Unité de Recherche Clinique (URC)

Paris Descartes Necker Cochin

Hôpital Necker-Enfants malades

Responsable : Pr Jean-Marc TRELUYER

Référent projet DRCI-URC : Nelly BRIAND

Tél 01 44 38 18 62

Courriel : [nelly.briand@aphp.fr](mailto:nelly.briand@aphp.fr)

**Structure chargée**

**du circuit du médicament :** Département Essais Clinique (DEC)

AGEPS-Paris

Pharmacien Référent projet: Dr Florence EMPANA-BARAT

Chef de projet : Elodie PERROT

Tél Secrétariat : 01 46 69 14 02

Courriel : [florence.barat@aphp.fr](mailto:florence.barat@aphp.fr) / [elodie.perrot@aphp.fr](mailto:elodie.perrot@aphp.fr)

Délégation à la Recherche Clinique et à l’Innovation (DRCI)

Hôpital Saint Louis 75010 PARIS

**Page de SIGNATURE D'UN PROTOCOLE de recherche**

Code de la Recherche : APHP180577

Titre : Evaluation de l’efficacité du chlorure de potassium dans la prise en charge des arrêts cardiaques extra-hospitaliers par fibrillation ventriculaire réfractaire

Version N° 1.1 du 19/12/2019

La recherche sera conduite conformément au protocole, aux bonnes pratiques en vigueur et aux dispositions législatives et réglementaires en vigueur.

| **L’investigateur coordonnateur** : |  |
| --- | --- |
| Pr Benoît Vivien  Service d’Anesthésie - Réanimation - SAMU Hôpital Necker – Enfants malades  Paris | Date : ……………/………/………..  Signature : |
| **Le promoteur** |  |
| Assistance Publique – Hôpitaux de Paris  Délégation à la Recherche Clinique et à l’Innovation (DRCI)  Hôpital Saint Louis  1 avenue Claude Vellefaux  75010 PARIS | Date : ……………/………/………..  Signature : |

**TABLE DES MATIÈRES**

1 RÉSUMÉ SYNOPTIQUE [5](#__RefHeading___Toc438386131)

2 JuSTIFICATION SCIENTIFIQUE de la recherche [8](#__RefHeading___Toc438386132)

2.1 Hypothèses de la recherche [8](#__RefHeading___Toc438386133)

2.2 Description des connaissances relatives à la pathologie concernée [8](#__RefHeading___Toc438386134)

2.3 Résumé des expérimentations pré-cliniques et des essais cliniques concernés [11](#__RefHeading___Toc438386135)

2.4 Pharmacocinétique du KCl [14](#__RefHeading___Toc438386136)

2.5 Description de la population à étudier et justification de son choix [15](#__RefHeading___Toc438386137)

2.6 Dénomination et description du ou des médicaments expérimentaux [16](#__RefHeading___Toc438386138)

2.7 Description et justification de la posologie, de la voie d'administration, du schéma d'administration et de la durée de traitement. [16](#__RefHeading___Toc438386139)

2.8 Résumé des bénéfices et des risques prévisibles et connus pour les personnes se prêtant à la recherche [17](#__RefHeading___Toc438386140)

3 objectifs [18](#__RefHeading___Toc438386141)

3.1 Objectif principal [18](#__RefHeading___Toc438386142)

3.2 Objectifs secondaires [18](#__RefHeading___Toc438386143)

4 conception de la recherche [19](#__RefHeading___Toc438386144)

4.1 Critères d’évaluation de la recherche [19](#__RefHeading___Toc438386145)

4.2 Description de la méthodologie de la recherche [20](#__RefHeading___Toc438386146)

5 Déroulement de la recherche [22](#__RefHeading___Toc438386147)

5.1 Inclusion [22](#__RefHeading___Toc438386148)

5.2 Intervention [23](#__RefHeading___Toc438386149)

5.3 Suivi dans la recherche [24](#__RefHeading___Toc438386150)

5.4 Visite de fin de la recherche [25](#__RefHeading___Toc438386151)

5.5 Schéma récapitulatif de la chronologie de la recherche [25](#__RefHeading___Toc438386152)

5.6 Durée prévue de participation des personnes, description de la chronologie et de la durée de la recherche. [27](#__RefHeading___Toc438386153)

5.7 Distinction soin-recherche [27](#__RefHeading___Toc438386154)

6 CRITERES D’ELIGIBILITE [28](#__RefHeading___Toc438386155)

6.1 Critères d’inclusion [28](#__RefHeading___Toc438386156)

6.2 Critères de non inclusion [28](#__RefHeading___Toc438386157)

6.3 Modalités de recrutement [28](#__RefHeading___Toc438386158)

6.4 Règles d’arrêt [29](#__RefHeading___Toc438386159)

7 TRAITEMENT ADMINISTRE AUX PERSONNES SE PRETANT A LA RECHERCHE [32](#__RefHeading___Toc438386160)

7.1 Description du ou des médicaments expérimentaux [32](#__RefHeading___Toc438386161)

7.2 Description du ou des traitements auxiliaires (médicaments nécessaires à la réalisation de la recherche) [32](#__RefHeading___Toc438386162)

7.3 Description des éléments de traçabilité qui accompagnent le ou les médicaments expérimentaux [32](#__RefHeading___Toc438386163)

7.4 Traitements (médicamenteux, auxiliaires, chirurgicaux) autorisés et interdits, y compris les médicaments de secours [33](#__RefHeading___Toc438386164)

7.5 Méthodes de suivi de l'observance au traitement [33](#__RefHeading___Toc438386165)

8 Evaluation de la securité – RISQUES ET CONTRAINTES AJOUTES PAR LA RECHERCHE [34](#__RefHeading___Toc438386166)

8.1 Procédures mises en place en vue de l’enregistrement et de la notification des évènements indésirables [34](#__RefHeading___Toc438386167)

8.2 Rôles du promoteur [39](#__RefHeading___Toc438386168)

9 gestion des donnees [42](#__RefHeading___Toc438386169)

9.1 Modalités de recueil des données [42](#__RefHeading___Toc438386170)

9.2 Identification des données recueillies directement dans les CRF qui seront considérées comme données-source [42](#__RefHeading___Toc438386171)

9.3 Droit d’accès aux données et documents source [42](#__RefHeading___Toc438386172)

9.4 Traitement des données et conservation des documents et des données [44](#__RefHeading___Toc438386173)

9.5 Propriété des données [44](#__RefHeading___Toc438386174)

10 aspects statistiques [45](#__RefHeading___Toc438386175)

10.1 Hypothèses de calcul du nombre de sujets nécessaires et résultat [45](#__RefHeading___Toc438386176)

10.2 Description des méthodes statistiques prévues y compris du calendrier des analyses intermédiaires prévues [45](#__RefHeading___Toc438386177)

11 contrôle ET ASSURANCE DE LA QUALITE [47](#__RefHeading___Toc438386178)

11.1 Organisation générale [47](#__RefHeading___Toc438386179)

11.2 Contrôle de qualité [48](#__RefHeading___Toc438386180)

11.3 Cahier d’observation [48](#__RefHeading___Toc438386181)

11.4 Gestion des non conformités [49](#__RefHeading___Toc438386182)

11.5 Audit / inspections [49](#__RefHeading___Toc438386183)

11.6 Engagement de responsabilités de l’Investigateur Principal [50](#__RefHeading___Toc438386184)

12 ASPECTS ETHIQUES ET LEGAUX [51](#__RefHeading___Toc438386185)

12.1 Modalités d’information et de recueil du consentement des personnes se prêtant à la recherche [51](#__RefHeading___Toc438386186)

12.2 Interdiction pour la personne de participer à une autre recherche ou période d’exclusion prévue à l’issu de la recherche, le cas échéant [52](#__RefHeading___Toc438386187)

12.3 Autorisation des lieux [52](#__RefHeading___Toc438386188)

12.4 Obligations légales [52](#__RefHeading___Toc438386189)

13 Financement et assurance [55](#__RefHeading___Toc438386190)

13.1 Source de financement [55](#__RefHeading___Toc438386191)

13.2 Assurance [55](#__RefHeading___Toc438386192)

14 regles relatives a la publication [56](#__RefHeading___Toc438386193)

14.1 Mention de l’affiliation de l’AP-HP pour les projets promus par l’AP-HP [56](#__RefHeading___Toc438386194)

14.2 Mention du promoteur AP-HP (DRCI) dans les ”acknowledgments” du manuscrit [56](#__RefHeading___Toc438386195)

14.3 Mention du financeur dans les ”acknowledgments” du manuscrit [56](#__RefHeading___Toc438386196)

15 bibliographie [57](#__RefHeading___Toc438386197)

16 LISTE des addenda [59](#__RefHeading___Toc438386198)

16.1 Liste des Investigateurs [59](#__RefHeading___Toc438386199)

16.2 Formulaire de notification des Evénements Indésirables Graves [60](#__RefHeading___Toc438386200)

16.3 Formulaire de notification et de suivi d’une grossesse [61](#__RefHeading___Toc438386201)

16.4 Echelle CPC score [62](#__RefHeading___Toc438386202)

# RÉSUMÉ SYNOPTIQUE

| Titre complet | Evaluation de l’efficacité du chlorure de potassium dans la prise en charge des arrêts cardiaques extra-hospitaliers par fibrillation ventriculaire réfractaire |
| --- | --- |
| Acronyme/référence | POTACREH |
| Investigateur coordonnateur | Pr Benoît Vivien |
| Responsable scientifique | Dr Romain Jouffroy |
| Promoteur | Assistance Publique – Hôpitaux de Paris |
| Justification scientifique | L’arrêt cardiaque extra-hospitalier (ACEH) est grevé d’un pronostic médiocre, globalement de l’ordre de 5% de survie à la sortie de l’hôpital. Les arrêts cardiaques (AC) de rythme « chocable » (fibrillation ventriculaire (FV) et tachycardie ventriculaire sans pouls), sont de meilleur pronostic.  En cas de rythme chocable, le traitement repose sur la défibrillation, puis en cas d’échec de 3 chocs électriques externes (CEE), sur l’administration de 300 mg d’amiodarone en intraveineux direct (IVD), suivi de 150 mg supplémentaires en cas d’inefficacité de 2 autres CEE. La lidocaïne, longtemps utilisée dans cette indication, n’est recommandée qu’en cas d’indisponibilité ou d’échec de l’amiodarone. Néanmoins, ces 2 médicaments, s’ils peuvent être efficaces pour faire céder une FV réfractaire, ont des effets cardiodépresseurs marqués, (bradycardie, et/ou effet inotrope négatif) persistants après leur administration IVD. Ceci explique, au moins partiellement, qu’une étude récente n’a pas montré de différence significative de survie à la sortie de l’hôpital entre amiodarone, lidocaïne et placebo chez des patients présentant un ACEH par FV réfractaire.  Lors d’interventions chirurgicales sous circulation extra-corporelle, une solution de cardioplégie est administrée pour interrompre l’activité cardiaque et faciliter le geste chirurgical. Sur le plan physio-pathologique, le mode d’action de ces solutions repose sur une concentration élevée en potassium permettant d’abaisser le potentiel de repos membranaire des myocytes. Par extension, il a été montré que l’administration IVD de 20 mmol de chlorure de potassium (KCl) permettait de faire céder une FV, entrainant le retour en quelques minutes à un rythme cardiaque organisé efficace sur le plan hémodynamique. Les kaliémies mesurées étaient à la limite supérieure de la normale (5,5 mmol/l) 10 min après cette injection, et normales à 20 min. Un cas clinique récent d’un patient de réanimation sous CEC en FV réfractaire a montré l’efficacité de l’injection IVD de 3 g de KCl, permettant d’obtenir un retour en rythme sinusal en quelques minutes.  L’avantage immédiat du KCl, par comparaison avec l’amiodarone (et également avec la lidocaïne), est l’absence d’effet cardiodépresseur (bradycardie et/ou hypotension) rémanent à distance de l’injection. Le mode d’action de l’injection IVD de KCl pour faire céder la FV est en effet lié au pic d’hyperkaliémie, et la kaliémie se normalisant ensuite rapidement en quelques minutes, il ne persiste consécutivement aucun effet délétère rémanent suite à cette injection de KCl. De plus, dans le cas d’un AC, le patient étant déjà sous massage cardiaque externe continu, aucune conséquence délétère liée à cette hyperkaliémie transitoire n’est attendue.  L’injection IVD de KCl chez un patient en ACEH présentant une FV réfractaire à 3 CEE, en lieu et place de l’amiodarone, devrait permettre d’interrompre cette FV et autoriser ensuite un retour rapide en rythme cardiaque organisé, et donc la restauration d’une activité cardiaque spontanée efficace. |
| Objectif et critère d’évaluation principal | L’objectif principal est d’évaluer, chez des patients en ACEH par FV réfractaire à 3 CEE, l’efficacité d’une injection IVD de 20 mmol de KCl sur la survie à l’admission hospitalière.  Le critère d’évaluation principal est la survie du patient à son arrivée à l’hôpital. |
| Objectifs et critères d’évaluation secondaires | Les objectifs secondaires sont, chez ces patients, d’évaluer l’efficacité d’une injection IVD de 20 mmol de KCl sur :   - le pourcentage de retour à une activité cardiaque spontanée en pré-hospitalier, - le temps de retour à une activité cardiaque spontanée en pré-hospitalier, - la dose totale d’adrénaline administrée en pré-hospitalier, - le nombre total de chocs électriques externes délivrés en pré-hospitalier, - le nombre de persistances ou récidives de trouble du rythme nécessitant un CEE en pré-hospitalier, - les paramètres hémodynamiques à l’admission à l’hôpital, - la survie à la sortie de l’hôpital avec un bon pronostic neurologique (CPC scores 1 et 2), - la survie à 3 mois avec un bon pronostic neurologique (CPC scores 1 et 2).   Les critères d’évaluation secondaires sont :   - le retour à une activité cardiaque spontanée en pré-hospitalier, - le temps de retour à une activité cardiaque spontanée en pré-hospitalier, - la dose totale d’adrénaline administrée en pré-hospitalier, - le nombre total de chocs électriques externes délivrés en pré-hospitalier, - le nombre de persistances ou récidives de trouble du rythme nécessitant un CEE en pré-hospitalier, - les paramètres hémodynamiques à l’admission à l’hôpital, - la survie à la sortie de l’hôpital avec un bon pronostic neurologique (CPC scores 1 et 2), - la survie à 3 mois avec un bon pronostic neurologique (CPC scores 1 et 2). |
| Schéma expérimental | Essai clinique prospectif non comparatif de phase II. |
| Population concernée | Patient majeur, victime d’un arrêt cardiaque extra-hospitalier d’origine cardiaque présumée, avec une fibrillation ventriculaire réfractaire malgré 3 chocs électriques externes. |
| Critères d’inclusion | - Patient majeur (âge ≥ 18 ans), - victime d’un ACEH d’origine cardiaque présumée avec une FV réfractaire malgré 3 CEE, - bénéficiant d’un régime d’assurance maladie. |
| Critères de non inclusion | - Grossesse avérée ; - Incapable majeur (patient sous tutelle ou curatelle) ; - Patient n’ayant encore pas de voie veineuse fonctionnelle après réalisation des 3 chocs électriques externes. |
| Traitement à l’essai | Chlorure de potassium (KCl) 20 mmol : Injection par voie intraveineuse directe (IVD) en 1 dose unique.  Phase de développement du traitement : phase 2. |
| Traitement de référence | NA |
| Autres actes ajoutés par la recherche | NA |
| Bénéfices attendus pour les participants | Améliorer la probabilité de survie de chaque patient en ACEH réfractaire participant à l’étude. |
| Risques ajoutés par la recherche | Il s’agit de patients en AC réfractaire, déjà sous massage cardiaque externe et ventilation artificielle. Dans ce contexte, aucune conséquence a priori délétère liée à cette hyperkaliémie transitoire n’est attendue.  Niveau de risque de la recherche : D |
| Déroulement pratique | Le plan expérimental est une phase II de Simon en 2 étapes.  * 34 patients seront inclus en 1ère étape.  * Si 12 succès ou moins sont observés, l’essai ne sera pas poursuivi et on conclura à l’inefficacité du KCl.  * Dans le cas contraire, si au moins 13 succès sont observés, l’essai se poursuivra avec l’inclusion de 47 patients supplémentaires.  * Le traitement sera considéré efficace selon ces hypothèses si au moins 34 succès sont observés au total. |
| Nombre de sujets sélectionnés | 81 |
| Nombre de centres | Recherche nationale multicentrique  4 centres extra-hospitaliers et 9 services de réanimation |
| Durée de la recherche | - durée d’inclusion : 18 mois - durée de participation (traitement + suivi) : 3 mois - durée totale de la recherche : 21 mois |
| Nombre d’inclusions prévues par centre et par mois | 1 à 2 patients / centre recruteur / mois |
| Analyse statistique | Analyse intermédiaire prévue après inclusion de 34 patients |
| Source de financement | Ministère de la Santé, PHRC Inter-Régional 2018 |
| Comité de Surveillance Indépendant prévu | Oui |

# JuSTIFICATION SCIENTIFIQUE de la recherche

## Hypothèses de la recherche

L’administration intraveineuse directe de chlorure de potassium (KCl) devrait permettre d’interrompre immédiatement une fibrillation ventriculaire (FV) réfractaire de patients victimes d’arrêt cardiaque extra-hospitalier (ACEH), permettant d’obtenir en quelques minutes le retour à un rythme cardiaque spontané organisé, associé à une reprise d’activité circulatoire spontanée efficace (RACS).

Cette modification de la stratégie de prise en charge des patients victimes d’ACEH par FV réfractaire devrait permet d’améliorer le pronostic de ces patients en terme de survie à la sortie de l’hôpital.

## Description des connaissances relatives à la pathologie concernée

L’arrêt cardiaque (AC) inopiné, ou mort subite de l’adulte est défini comme l’interruption brutale de la circulation spontanée. Cette pathologie représente un problème majeur de santé publique dans les pays industrialisés, qui concerne annuellement plus de 420 000 patients aux USA. En France, on estime qu’il survient entre 30 0000 et 50 000 arrêts cardiaques (AC) par an, dont 85% en milieu extra-hospitalier **[1]**.

La classification des arrêts cardiaques, aussi bien intra-hospitaliers (ACIH) qu’extra-hospitaliers, repose sur l’analyse du premier rythme identifié par le tracé électrocardioscopique. Ainsi, on distingue d’une part les rythmes choquables, c’est-à-dire justifiant l’administration d’un choc électrique externe (CEE) : fibrillation ventriculaire (FV) et tachycardie ventriculaire (TV) sans pouls ; et d’autre part les rythmes non-choquables : asystolie et rythmes sans pouls **[2]**. Le pronostic des AC par rythme choquable est meilleur que celui des AC par rythme non choquable, en raison de l’efficacité potentielle d’un CEE permettant un retour à un rythme cardiaque spontané efficace sur le plan hémodynamique.

L’ACEH est grevé d’un pronostic médiocre, avec un taux de survie global à la sortie de l’hôpital extrêmement faible, classiquement considéré comme voisin de 5% **[2]**. Une méta-analyse publiée en 2010 avait montré que le pronostic de l’ACEH avait peu évolué durant les 30 années précédant cette publication, avec un taux global de survie de 7,6% à la sortie de l’hôpital **[3]**. Des données plus récentes suggèrent cependant que le pronostic global des ACEH s’est sensiblement amélioré, passant de 8,2% de survie à la sortie de l’hôpital en 2006 à 10,4% en 2010. En revanche, si l’on s’intéresse plus spécifiquement aux patients victimes d’un ACEH par rythme chocable (FV et TV sans pouls), rythmes de meilleur pronostic car accessibles à une défibrillation précoce, celui-ci passe de 23,5% de survie à la sortie de l’hôpital en 2006 à 30,3% en 2010 **[4]**.

La prise en charge d’un patient en AC suit des recommandations internationales établies par un consensus d’experts et regroupées au sein de l’ILCOR (*International Liaison Committee on Resuscitation*). La dernière version de ces recommandations de l’ILCOR a été publiée en 2015 par l’*American Heart Association* (AHA) **[5]** et par l’*European Resuscitation Council* (ERC) **[6]**. Entre autres éléments, l’algorithme de la réanimation cardio-pulmonaire (RCP) différencie notamment la prise en charge des rythmes chocables de celle des rythmes non-chocables, de par la réalisation d’un CEE dans le premier cas (fig. 1).


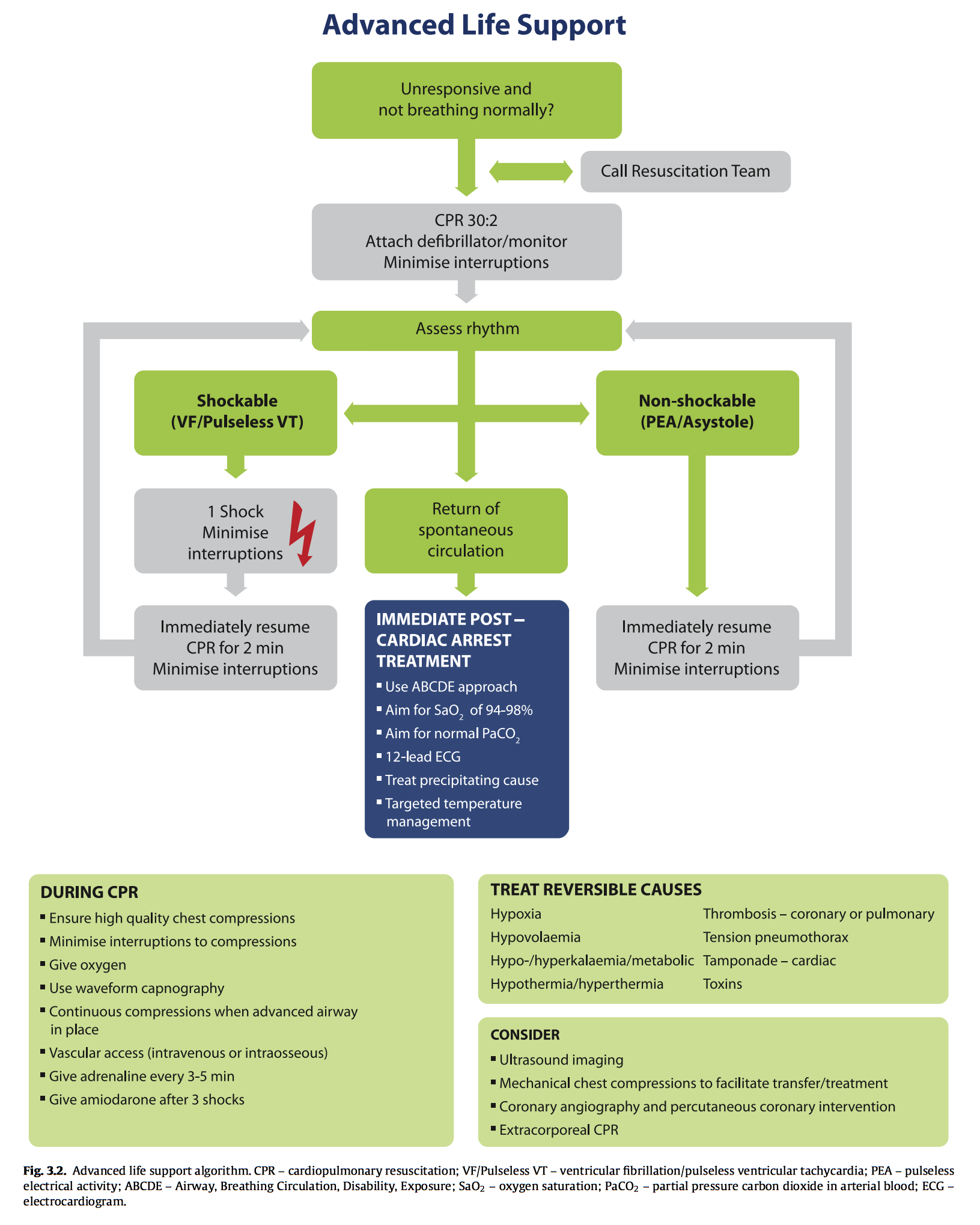


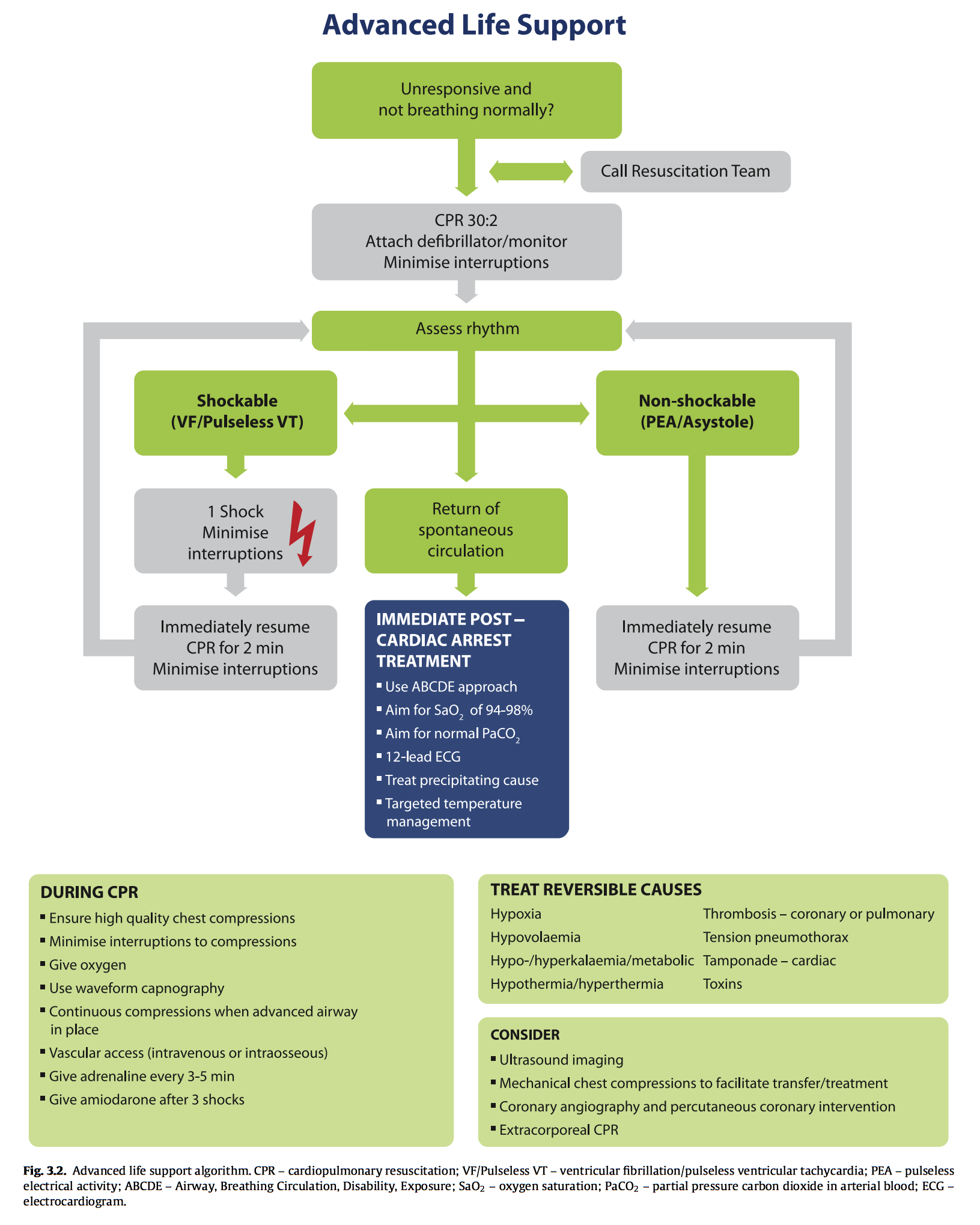


**Figure 1 :** Algorithme de la RCP médicalisée, version de l’*European Resuscitation Council* (ERC) **[6]**.

Si dans le cas d’un rythme chocable, la défibrillation permet le plus souvent le retour d’un rythme cardiaque efficace sur le plan hémodynamique, cette thérapeutique est parfois inefficace. C’est pourquoi les recommandations internationales préconisent alors l’injection intraveineuse d’un médicament antiarythmique au-delà du 3^ème^ CEE. Deux médicaments sont classiquement recommandés dans cette indication qualifiée d’AC par rythme chocable « réfractaire » aux CEE : l’amiodarone (300 mg IVD) en première intention, et la lidocaïne (1 mg/kg) en deuxième intention **[2]**.

Néanmoins, si ces deux médicaments peuvent, selon les études publiées dans la littérature, permettre d’augmenter le taux de retour à une activité circulatoire spontanée efficace, et/ou le taux de patients admis vivants à l’hôpital, il faut noter que les résultats sont beaucoup plus mitigés concernant la survie à 3 mois et/ou à la sortie de l’hôpital, ce qui est le critère « fort » généralement utilisé pour évaluer la survie après un ACEH. Ainsi, l’une des études majeures les plus récentes sur ce sujet a été publiée dans le *New England Journal of Medicine* par Kudenchuk et al. en 2016 **[7]**. Dans ce travail les auteurs ont comparé l’administration d’amiodarone à celle de lidocaïne et à un placebo, chez 3026 patients en ACEH par FV ou TV sans pouls réfractaire à 3 CEE. Si l’amiodarone et la lidocaïne permettaient effectivement une augmentation du taux de patients admis vivants à l’hôpital (respectivement 45,7% *vs* 39,7%, p=0,01 ; et 47,0% *vs* 39,7%, p<0,001), sans différence statistique entre ces 2 molécules (45,7% *vs* 47,0%, p=0,55), il faut noter qu’il n’y avait pas de différence significative en terme de survie à la sortie de l’hôpital entre amiodarone, lidocaïne et placebo (amiodarone *vs* placebo : 24,4% *v*s 21,0%, p=0,08 ; lidocaïne *vs* placebo : 23,7% *vs* 21,0% p=0,16 ; amiodarone *vs* lidocaïne : 24,4% *vs* 23,7%, p=0,70).

Ces résultats non significatifs en termes de survie peuvent probablement être expliqués par les propriétés pharmacocinétiques et pharmacodynamiques des 2 molécules antiarythmiques que sont l’amiodarone et de la lidocaïne. L’amiodarone, antiarythmique de classe III selon la classification de Vaughan-Williams, a notamment pour effets secondaires un effet bradycardisant et un effet dromotrope négatif (ralentissement de la conduction), plus marqués lors d’une administration à forte dose et par voie intraveineuse, ce qui est le cas lors d’un AC réfractaire. La lidocaïne, antiarythmique de classe Ib selon la classification de Vaughan-Williams, a notamment pour effet secondaire un effet dépresseur myocardique, caractérisé par une hypotension, une dépression myocardique et une bradycardie. Il est donc parfaitement possible que les effets bénéfiques de ces 2 molécules en termes de RACS immédiat soient contrebalancés par leurs effets secondaires négatifs « rémanents » sur le plan cardiovasculaire lors d’une administration intraveineuse à forte dose, et donc finalement ne permettent pas d’améliorer la survie à la sortie de l’hôpital.

Par ailleurs, il faut noter que les taux de survie à la sortie de l’hôpital dans les groupes amiodarone (24,4%), lidocaïne (23,7%) et placebo (21%) de l’étude de Kudenchuk et al. **[7]** peuvent sembler en première approximation très inférieurs aux 30,3% de survie à la sortie de l’hôpital de l’étude de Daya et al. publiée en 2010 **[4]**. Mais il faut observer que Daya et al. analysaient l’ensemble des patients présentant un rythme chocable, alors que Kudenchuk et al. analysaient uniquement des patients présentant un rythme chocable déjà réfractaire à 3 CEE, ce qui explique ce pronostic plus péjoratif dans cette deuxième étude.

Il apparaît donc lors de l’analyse de la littérature qu’aucune molécule n’est plus efficace qu’un placebo en terme de survie à la sortie de l’hôpital chez des patients présentant un ACEH par FV ou TV sans pouls réfractaire à 3 CEE. D’autres voies de recherche pharmacologiques sont par conséquent nécessaires pour tenter d’améliorer le pronostic de ces patients.

## Résumé des expérimentations pré-cliniques et des essais cliniques concernés

Lors des interventions chirurgicales effectuées sous circulation extracorporelle (CEC), notamment en chirurgie cardiaque valvulaire et/ou coronaire, une solution de cardioplégie est administrée pour interrompre l’activité mécanique du cœur et ainsi faciliter le geste chirurgical. Diverses solutions de cardioplégie sont disponibles, variant dans leur composition chimique. Mais sur le plan physiopathologique, l’élément essentiel de ces solutions est toujours représenté par le chlorure de potassium à une concentration élevée, habituellement de l’ordre de 20 à 30 mmol/L **[8]**. L’administration intraveineuse directe de cette solution est alors responsable d’une hyperkaliémie transitoire, qui a pour conséquence immédiate d’abaisser le potentiel de repos membranaire des myocytes, entrainant la cessation de leur activité électrique et mécanique, et consécutivement alors l’immobilité du muscle cardiaque.

Ces solutions de cardioplégie étant efficaces pour stopper l’activité électrique myocardique lors de la mise en œuvre d’une CEC, certains auteurs ont par extension évalué leur efficacité pour interrompre une fibrillation ventriculaire, essentiellement dans le contexte du déclampage aortique après CEC, à l’exception du premier cas clinique présenté ci-après.

En effet, la première publication scientifique décrivant l’administration de chlorure de potassium chez l’homme pour convertir une fibrillation ventriculaire est un cas clinique publié par Weinstock et Clark en 1961 **[9]**. Les auteurs y rapportent le cas d’un enfant de 3 ans présentant un AC avec FV réfractaire au décours d’une intubation impossible au bloc opératoire, et qui sera finalement convertie en rythme sinusal grâce à l’injection intracardiaque de 4 mmol de chlorure de potassium. L’enfant a par la suite survécu avec un développement psychomoteur totalement normal d’après les auteurs.

En 1984, Robicsek et al. ont publié une série de 12 patients chez lesquels une injection de chlorure de potassium avait permis de faire céder une FV survenue au décours d’une chirurgie cardiaque avec CEC et cardioplégie **[10]**.

Onze ans plus tard, Øvrum et al. ont décrit une série de 200 patients opérés de chirurgie cardiaque sous CEC et cardioplégie **[11]**. Une injection de 20 mmol de chlorure de potassium a été effectuée chez 100 patients, permettant de faire céder la FV dans 82% des cas. Les 18 autres patients ayant reçu 20 mmol de chlorure de potassium sans avoir permis de régulariser la FV ont nécessité un nombre plus faible de CEE (p<0,005) pour faire céder la FV, par comparaison avec les 100 patients du groupe contrôle.

Une étude très similaire à la précédente a été publiée par Almdahl et al. en 2013 sur un collectif de 8465 patients opérés sous CEC avec cardioplégie **[12]**. Dans ce travail, 1721 patients présentant une FV au décours du déclampage aortique ont reçu une injection de chlorure de potassium, qui a permis de faire céder le trouble du rythme chez 1366 d’entre eux (79,1%). Parmi tous les patients chez lesquels cette injection a été efficace, la dose de potassium nécessaire était de 20 mmol dans 88% des cas, et 30 mmol dans 12% des cas. Aucun effet délétère lié à cette injection de chlorure de potassium n’était rapporté dans cette étude. Seulement 355 patients (20,9%), chez lesquels l’injection de chlorure de potassium était inefficace, ont nécessité un ou plusieurs chocs électriques pour faire céder la FV. L’avantage essentiel de cette « défibrillation pharmacologique » mis en avant par les auteurs est l’absence de nécessité d’interrompre la chirurgie pour la mise en place des palettes de défibrillation, et consécutivement l’absence de risque de lésion iatrogène des pontages coronaires liées au positionnement de ces palettes et au mouvement induit lors du CEE.

En dehors de ces cas de FV survenant au décours d’une chirurgie cardiaque sous CEC et cardioplégie, plusieurs publications rapportent l’efficacité d’une injection de chlorure de potassium pour traiter une FV ou TV sans pouls réfractaire à une défibrillation par chocs électriques.

Ainsi, sur un modèle animal porcin (35±2 kg), Liakopoulos et al. ont montré l’efficacité d’une injection de chlorure de potassium (15±2 mEq) lors d’une FV durant depuis plus de 10 minutes **[13]**.

Une série de 3 patients présentant un orage rythmique avec TV et FV réfractaires au décours du déclampage aortique après CEC a été publiée en 2011 par Watanabe et al. **[14]**. Ces 3 patients présentaient une FV ou TV réfractaire malgré 5 à 10 chocs électriques internes de 50 J. Une injection de 20 mEq de chlorure de potassium a permis chez chacun d’entre eux de faire céder le trouble du rythme ventriculaire réfractaire, avec le retour à un rythme cardiaque organisé et efficace sur le plan hémodynamique.

Enfin, nous avons publié récemment le cas d’un patient de réanimation sous CEC ayant présenté une FV réfractaire au décours d’un ACEH d’origine ischémique **[15]**. L’injection IVD de 3 g (40 mmol) de chlorure de potassium a permis le retour à un rythme sinusal en quelques minutes, sans récidive ultérieure de troubles de rythme, et le patient est sorti vivant d’hospitalisation avec un bon pronostic sur le plan neurologique.

Ces différentes études et cas cliniques rapportant l’efficacité d’une injection de chlorure de potassium sont supportés par des données expérimentales. Ainsi, il a été montré sur un modèle de fibre endocardique isolée qu’une concentration de 12 mmol/l de chlorure de potassium permettait de faire céder une FV, avec consécutivement un retour en rythme électrique organisé **[16]**. Une étude électrophysiologique récente sur un modèle de cœur isolé a permis de montrer que l’hyperkaliémie agissait principalement sur le caractère dynamique et sur l’organisation temporo-spatiale de la FV **[17]**.

La question qui se pose évidemment au décours d’une injection de chlorure de potassium est la pharmacocinétique de cette hyperkaliémie. Si les études sont limitées dans ce domaine, des données sont cependant disponibles dans la littérature. Ainsi, dans leur série de 100 patients ayant reçu une injection de 20 mmol de chlorure de potassium, Øvrum et al. rapportent des valeurs de kaliémie à la limite supérieure de la normale (5,5±1,0 mmol/l) 10 minutes après l’injection, et revenues dans les valeurs normales (4,3±0,4 mmol/l) après 20 minutes **[11]**. Ceci atteste donc du caractère extrêmement transitoire de cette hyperkaliémie lors d’une injection intraveineuse directe de 20 mmol de chlorure de potassium.

Enfin, concernant l’éventuelle morbimortalité liée à une injection de chlorure de potassium, Almdahl et al. ont publié des données rassurantes dans un 2^ème^ article **[18]** utilisant la même méthodologie que leur premier article **[12]**. Sur une série de 12113 patients opérés de chirurgie cardiaque sous CEC et cardioplégie, la survie à J30 n’était pas différente entre les 9723 patients n’ayant pas présenté de FV post-CEC et les 1877 patients ayant présenté une FV convertie avec succès par une injection de 20 mmol de chlorure de potassium (1,2% vs 1,32%, p=0,269). En revanche, il existait une tendance à une augmentation de mortalité à long terme chez les 400 patients ayant présenté une FV résistant à la « défibrillation pharmacologique » et ayant donc nécessité un choc électrique interne (hazard ratio=1,19 IC95% [0,99-1,4], p=0,07). Ces résultats sont donc rassurants vis-à-vis d’éventuelles conséquences délétères qui auraient pu être liées à l’injection de chlorure de potassium. Ils laissent par ailleurs envisager (mais avec toutes les réserves de résultats non statistiquement significatifs) que cette défibrillation pharmacologique pourrait améliorer la survie par comparaison avec une défibrillation classique par chocs électriques (en l’occurrence chocs électriques internes dans ce travail puisqu’il s’agit de patients opérés de chirurgie cardiaque).

L’ensemble de ces travaux permet donc d’envisager qu’une injection intraveineuse directe de 20 mmol de chlorure de potassium, chez un patient en ACEH présentant une FV ou TV sans pouls réfractaire à 3 CEE, permette, grâce à une hyperkaliémie transitoire, de faire céder ce trouble du rythme et consécutivement autoriser le retour à un rythme cardiaque organisé et efficace sur le plan hémodynamique, et ce sans conséquence délétère immédiate ni à long terme.

## Pharmacocinétique du KCl

L'organisme contient 3 500 mmol de potassium qui se répartissent pour 98 % dans le secteur intracellulaire, et représentent la majeure partie du potassium échangeable. Les hématies stockent 2 % du potassium intracellulaire, soit 70 mmol. Le secteur extracellulaire contient 80 mmol de K+, soit 2 % du potassium total. Le maintien du gradient entre les secteurs extra-cellulaire (Ke) et intra-cellulaire (Ki) se fait activement par la pompe Na+K+-ATPase et par l'électronégativité intracellulaire.

Les variations à court terme de la kaliémie, comme par exemple lors de l’administration d’une dose de charge de potassium par voie intraveineuse directe, sont régulées par ces échanges transmembranaires, essentiellement liés à la pompe Na+K+-ATPase. A contrario, l'excrétion urinaire du potassium est un phénomène beaucoup plus lent, dépendant de sa sécrétion tubulaire dans le néphron distal qui ajuste les pertes rénales aux apports quotidiens **[19]**. Il faut donc considérer que le rein régule la balance potassique non pas à court terme, mais à moyen terme **[20]**.


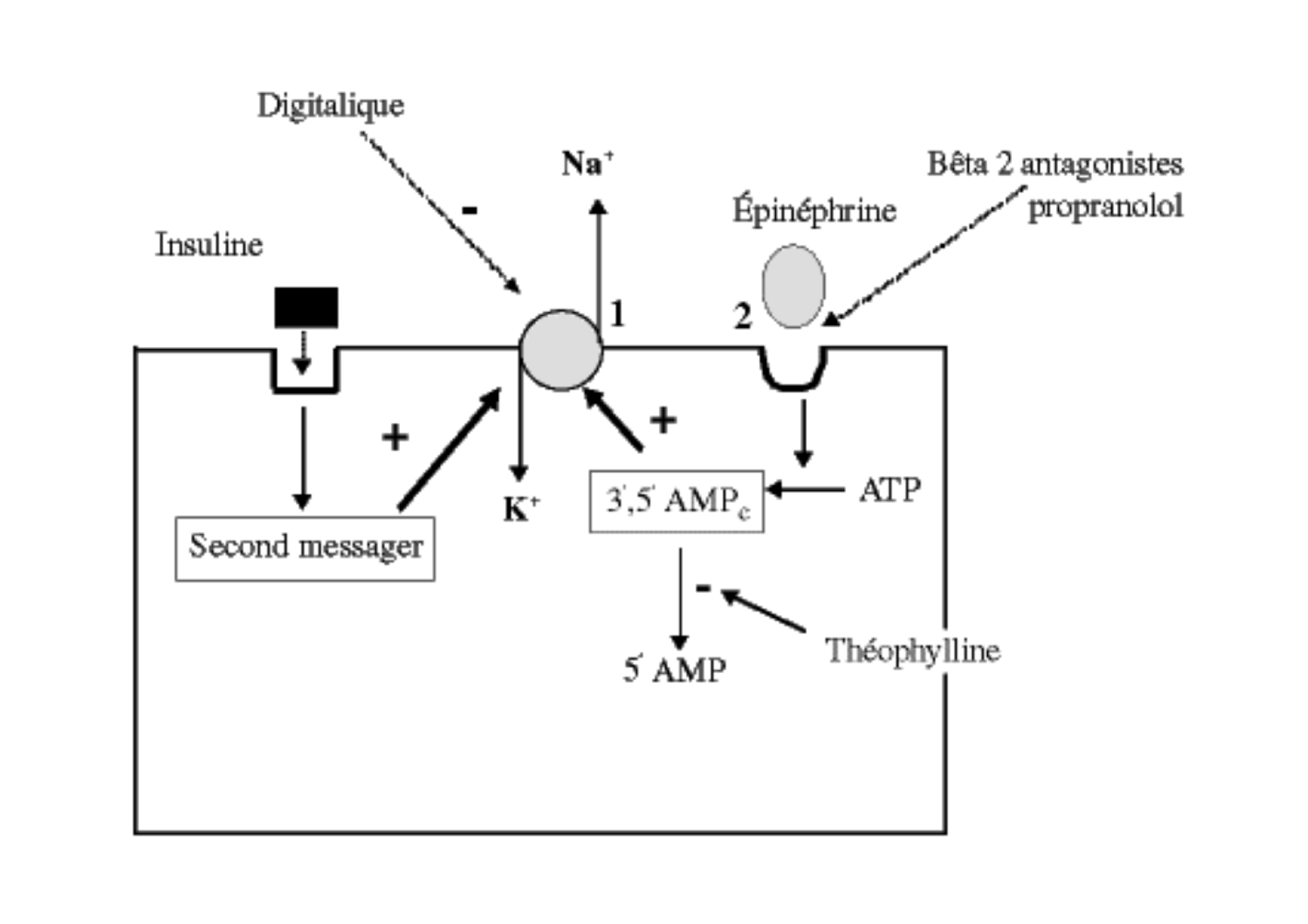


**Figure 3 :** Régulation des flux du potassium entre les compartiments intra-cellulaire et extra-cellulaire (d’après **[21]**).

Ce mode de régulation de la kaliémie à court terme lié à la pompe Na+K+-ATPase permet d’expliquer qu’une injection de 20 mmol de potassium par voie intraveineuse directe se traduise par une hyperkaliémie transitoire, avec le retour à une valeur de kaliémie à la limite supérieure de la normale 10 min. après l’injection, et revenues dans les valeurs normales après 20 min. Corolaire de cette diminution rapide de la kaliémie lors d’une injection intraveineuse directe, il peut être considéré que l’élimination rénale du potassium joue par conséquent un rôle mineur dans cette décroissance, par comparaison avec la mise en jeu immédiate de la pompe Na+K+-ATPase qui va favoriser le transfert du potassium depuis le compartiment extra-cellulaire vers le compartiment intra-cellulaire. Consécutivement, chez un patient insuffisant rénal, l’absence complète d’élimination urinaire ne devrait avoir que peu d’impact sur la pharmacocinétique du potassium par comparaison avec un sujet ayant une fonction rénale conservée.

Enfin, il faut également mentionner le rôle des catécholamines, qui favorisent le transfert intracellulaire du potassium par stimulation des récepteurs beta 2 adrénergiques via l’AMPc **[22]**. Dans le cas d’un patient en arrêt cardiaque, l’administration de forte doses (1 mg IVD) d’adrénaline, stimulant à la fois des récepteurs alpha et beta-adrénergiques, va donc activer encore plus la Na+K+-ATPase transmembranaire, ce qui va d’autant favoriser le transfert du potassium depuis le compartiment extra-cellulaire vers le compartiment intra-cellulaire.

## Description de la population à étudier et justification de son choix

La population étudiée concernera les patients majeurs (âge supérieur ou égal à 18 ans) victimes d’un arrêt cardiaque extra-hospitalier d’origine cardiaque présumée et présentant un rythme chocable, c’est-à-dire une fibrillation ventriculaire, réfractaire malgré la réalisation de 3 chocs électriques externes.

Le choix de cette population est effectué en raison de l’absence de traitement anti-arythmique (amiodarone et lidocaïne) ayant fait preuve d’une efficacité supérieure contre placebo sur l’amélioration sur la survie à la sortie de l’hôpital.

A contrario, il a été observé l’efficacité en chirurgie cardiaque et en réanimation de l’administration de chlorure de potassium IVD pour interrompre une FV ou TV sans pouls réfractaire, sans effet délétère et en autorisant un bon pronostic neurologique à la sortie de l’hôpital.

Il est donc licite d’évaluer l’efficacité d’une injection de chlorure de potassium IVD pour interrompre une fibrillation ventriculaire réfractaire à 3 chocs électriques externes.

## Dénomination et description du ou des médicaments expérimentaux

- Dénomination du médicament : Chlorure de potassium (KCl) ampoule à 10%, solution pour perfusion.

- Posologie utilisée : 20 mmol de chlorure de potassium soit 14,9 ml de KCl à 10%, chiffre qui de manière pragmatique est arrondi à 15 ml de KCl à 10%.

- Modalités et voie d’administration : administration unique intraveineuse directe (IVD).

## Description et justification de la posologie, de la voie d'administration, du schéma d'administration et de la durée de traitement.

La posologie de 20 mmol de KCl en injection intraveineuse directe pour cette étude est celle qui a été utilisée dans la plupart des études présentées précédemment chez l’adulte. En revanche il faut préciser que dans ces études, le KCl était généralement administré en intra-aortique et/ou par l’intermédiaire de la CEC, ce qui n’est évidemment pas possible dans notre étude sur des patients en arrêt cardiaque extra-hospitalier. La voie d’administration sera donc intraveineuse périphérique directe. Il s’agit d’une administration « flash », unique, dans le but d’obtenir un pic d’hyperkaliémie, donc la durée de traitement stricto sensu (= durée d’administration) sera de l’ordre de quelques secondes.

Parallèlement à cette injection, la réanimation cardiopulmonaire spécialisée sera effectuée de manière conventionnelle conformément aux recommandations internationales et aux bonnes pratiques cliniques, notamment en terme de massage cardiaque externe et de ventilation. La seule différence par comparaison aux recommandations internationales s’effectuera lorsque le patient aura reçu 3 CEE : en lieu et place des 300 mg d’amiodarone IVD classiquement recommandés, le patient recevra une dose unique de 20 mmol de chlorure de potassium IVD (soit 14,9 ml arrondie à 15 ml de KCl à 10%). L’administration d’amiodarone 300 mg, si elle est nécessaire en raison d’une FV réfractaire ou récidivante, sera simplement « retardée » de 2 CEE dans le cadre de la recherche.

La réanimation cardiopulmonaire spécialisée sera ensuite poursuivie de manière conventionnelle, toujours conformément aux recommandations internationales. En particulier, les chocs électriques seront à nouveau effectués si le patient présente une fibrillation ventriculaire persistante ou récidivante après cette injection de 20 mmol de chlorure de potassium IVD.

## Résumé des bénéfices et des risques prévisibles et connus pour les personnes se prêtant à la recherche

Cette injection de chlorure de potassium 20 mmol IVD pourrait permettre d’interrompre immédiatement une FV réfractaire, en permettant consécutivement le retour rapide à un rythme cardiaque spontané efficace sur le plan hémodynamique.

L’efficacité du chlorure de potassium pour interrompre une FV réfractaire est en effet liée au pic d’hyperkaliémie, et il a été montré que la kaliémie mesurée 10 minutes après cette injection est à la limite de la normale (5,5 mmol/l) et normale après 20 minutes. Il n’y a donc pas de risque d’un effet dépresseur persistant sur le plan cardiovasculaire une fois le pic d’hyperkaliémie disparu. Par ailleurs, la 2^ème^ étude d’Almdahl et al., certes dans un contexte post-opératoire de chirurgie cardiaque sous CEC, a bien montré l’absence de morbimortalité spécifique chez les 1877 patients ayant présenté une FV convertie avec succès par une injection de 20 mmol de chlorure de potassium **[18]**.

A contrario, l’amiodarone et la lidocaïne, molécules de références selon les recommandations internationales pour interrompre une FV réfractaire, sont caractérisées par des effets dépresseurs sur le plan cardiovasculaire, persistant bien après leur administration intraveineuse directe.

L’injection de chlorure de potassium IVD, en lieu et place de l’amiodarone ou de la lidocaïne, pourrait donc permettre comme ces 2 dernières molécules d’interrompre immédiatement une FV réfractaire, mais en revanche sans présenter le risque d’un effet dépresseur persistant sur le plan cardiovasculaire.

Le risque éventuel de cette injection IVD de chlorure de potassium peut être considéré comme nul, puisque le patient est déjà en arrêt cardiaque réfractaire, sous réanimation cardiopulmonaire spécialisée, donc avec un massage cardiaque externe permettant de pallier l’absence d’activité cardiaque spontanée, et une ventilation artificielle manuelle ou mécanique permettant de pallier l’absence de ventilation spontanée.

L’efficacité éventuelle de l’injection de chlorure de potassium dans le traitement des ACEH par FV réfractaire serait donc susceptible d’améliorer le pronostic des patients présentant une FV réfractaire lors d’un ACEH. Cela devrait donc avoir logiquement pour conséquence une modification des recommandations internationales concernant cette pathologie.

# objectifs

## Objectif principal

L’objectif principal est d’évaluer chez des patients en arrêt cardiaque extra-hospitalier par fibrillation ventriculaire réfractaire à 3 chocs électriques externes l’efficacité d’une injection IVD de 20 mmol de chlorure de potassium sur la survie à l’admission hospitalière, c’est-à-dire sur le pourcentage de patients arrivant à l’hôpital avec une activité cardiaque spontanée efficace.

## Objectifs secondaires

Les objectifs secondaires sont, chez ces patients, d’évaluer l’efficacité d’une injection IVD de 20 mmol de chlorure de potassium sur :

- Le retour à une activité cardiaque spontanée efficace en pré-hospitalier,
- Le temps de retour à une activité cardiaque spontanée en pré-hospitalier,
- La dose totale d’adrénaline administrée en pré-hospitalier,
- Le nombre total de chocs électriques externes délivrés en pré-hospitalier,
- Le nombre de persistances et/ou récidives de trouble du rythme nécessitant un choc électrique externe en pré-hospitalier,
- Les paramètres hémodynamiques à l’admission à l’hôpital,
- La survie à la sortie de l’hôpital avec un bon pronostic neurologique (*Cerebral Performance Category* scores 1 et 2),
- La survie à 3 mois avec un bon pronostic neurologique (*Cerebral Performance Category* scores 1 et 2).

# conception de la recherche

## Critères d’évaluation de la recherche

### Critère d’évaluation principal

Le critère d’évaluation principal est la survie du patient à son arrivée à l’hôpital (avec une activité cardiaque spontanée efficace).

L’évaluation sera qualitative : présence ou absence d’une activité cardiaque spontanée efficace mesurée au moment de l’arrivée du patient dans le service hospitalier (réanimation ou salle de cathétérisme cardiaque).

### Critères d’évaluation secondaires

Les critères d’évaluation secondaires sont :

- Le retour à une activité cardiaque spontanée en pré-hospitalier. L’évaluation sera qualitative : présence ou absence d’une activité cardiaque spontanée efficace avant l’arrivée du patient à l’hôpital.

- Le temps de retour à une activité cardiaque spontanée en pré-hospitalier. L’évaluation sera quantitative : délai en minutes entre le moment de l’arrêt cardiaque et le retour à une activité cardiaque spontanée efficace.

- La dose totale d’adrénaline administrée en pré-hospitalier. L’évaluation sera quantitative : le nombre total de milligrammes d’adrénaline administrés avant l’arrivée du patient dans le service hospitalier (réanimation ou salle de cathétérisme cardiaque) sera relevé.

- Le nombre total de chocs électriques externes délivrés en pré-hospitalier. L’évaluation sera quantitative : le nombre total de choc délivrés avant l’arrivée du patient dans le service hospitalier (réanimation ou salle de cathétérisme cardiaque) sera relevé.

- Le nombre de persistances et/ou récidives de trouble du rythme nécessitant un choc électrique externe en pré-hospitalier. L’évaluation sera quantitative : le nombre total de trouble du rythme nécessitant un choc électrique externe avant l’arrivée du patient dans le service hospitalier (réanimation ou salle de cathétérisme cardiaque) sera relevé.

- Les paramètres hémodynamiques à l’admission à l’hôpital. L’évaluation sera quantitative : la fréquence cardiaque et la pression artérielle seront mesurées au moment de l’arrivée du patient dans le service hospitalier (réanimation ou salle de cathétérisme cardiaque).

- La survie à la sortie de l’hôpital avec un bon pronostic neurologique (*Cerebral Performance Category* scores 1 et 2). A la sortie de l’hôpital, l’évaluation sera qualitative pour la survie (présente ou absente) et quantitative pour le score CPC coté sur une échelle de 1 à 5.

- La survie à 3 mois avec un bon pronostic neurologique (*Cerebral Performance Category* scores 1 et 2). A 3 mois, l’évaluation sera qualitative pour la survie (présente ou absente) et quantitative pour le score CPC coté sur une échelle de 1 à 5.

## Description de la méthodologie de la recherche

### Plan expérimental

Il s’agit d’un essai clinique prospectif non comparatif de phase II.

L’objectif de cet essai de phase II est de pouvoir disposer dans un premier temps d’une première évaluation de l’efficacité du traitement. Si l’efficacité s’avère être satisfaisante (survie à l’admission proche de 50%), un essai randomisé national sera mené pour comparer la stratégie incluant le chlorure de potassium en première intention à la stratégie classique.

En l’absence de données préliminaires d’efficacité dans l’arrêt cardiaque extra-hospitalier par FV réfractaire, il n’aurait pas été pertinent de proposer d’emblée un essai randomisé contre placebo. En effet, dans ce contexte, pour montrer une amélioration de la survie cliniquement significative, il faudrait inclure un grand nombre de sujets (par exemple 1500 sujets par groupe pour montrer une amélioration de la survie de 35% à 40%).

### Nombre de centres participants

Les centres participant à cette recherche multicentrique régionale sont :

- D’une part des services d’urgences pré-hospitaliers qui assureront le recrutement pré-hospitalier des patients participant à cette étude,
- D’autre part, des services de réanimation dans lesquels seront hospitalisés les patients au décours de leur prise en charge pré-hospitalière.

### Identification des sujets

Dans le cadre de cette recherche, les sujets seront identifiés de la façon suivante :

n°centre (3 positions numériques) – n°ordre de sélection de la personne dans le centre (4 positions numériques) - initiale nom - initiale prénom.

Cette référence est unique et sera conservée pour toute la durée de la recherche.

# Déroulement de la recherche

**Avant tout examen ou acte lié à la recherche, l’investigateur recueille le consentement *libre, éclairé et écrit de la personne qui se prête à la recherche ou de son représentant* légal le cas échéant.**

**Les personnes susceptibles de se prêter à des recherches mentionnées au 1° de l'article L. 1121-1 du Code de la santé publique bénéficient d'un examen médical préalable adapté à la recherche.**

## Inclusion

La sélection a lieu lors de la prise en charge pré-hospitalière par une équipe d’un des centres participants d’un patient présentant un ACEH. Si ce patient présente un rythme chocable de type FV, alors il sera considéré comme éligible. La sélection aura par conséquent lieu quelques minutes avant l’inclusion le cas échéant.

Après vérification des critères d’inclusion et de non-inclusion, l’investigateur inclura le patient s’il répond aux critères définis par l’étude. Il s’agira en résumé d’un patient présentant un ACEH avec une FV authentifiée sur le monitorage et pour laquelle 3 CEE n’ont pas permis d’obtenir le retour à un rythme cardiaque spontané efficace sur le plan hémodynamique.

L’inclusion se fera donc essentiellement sur des données anamnestiques concernant l’ACEH, des données cliniques et le monitorage du rythme cardiaque du patient. Aucun examen complémentaire biologique ou d’imagerie n’est nécessaire pour permettre l’inclusion du patient dans l’étude.

S’agissant d’une urgence vitale immédiate, une dérogation à l’obligation de la recherche du consentement préalablement à l’inclusion du patient est requise (article L1122-1-3 du Code de Santé Publique).

## Intervention

La figure 2 présente l’algorithme de prise en charge d’un patient en ACEH par fibrillation ventriculaire selon les recommandations internationales (fig. 3A) et dans le cadre de la recherche (fig. 3B).

**Figure 3 :** Algorithme de prise en charge d’un patient en ACEH par FV selon les recommandations internationales (fig. 3A) et dans le cadre de la recherche (fig. 3B). Les injections d’adrénaline ne sont pas représentées sur la figure dans un souci de simplification.

Selon les recommandations internationales **[5,6]**, une administration de 300 mg IVD d’amiodarone doit être effectuée en cas de FV réfractaire (ou récidivante) après 3 CEE. Si la FV perdure, une deuxième administration d’amiodarone IVD doit être effectuée à demi-dose (150 mg) après le 5^ème^ CEE. Si la FV persiste toujours, il est alors possible de débuter une administration IV continue à la seringue électrique à la dose de 900 mg/j, ou de recourir à une administration intraveineuse directe de lidocaïne (1 mg/kg).

Dans le cadre de la recherche, **l’injection intraveineuse directe de 20 mmol de chlorure de potassium sera effectuée après le 3^ème^ CEE, en lieu et place de la 1^ère^ administration d’amiodarone**. Si nécessaire, la 1^ère^ injection d’amiodarone 300 mg IVD sera effectuée après le 5^ème^ CEE, la 2^ème^ injection 150 mg IVD après le 7^ème^ CEE, avec un relais par l’amiodarone en continu ou le recours à la lidocaïne ensuite.

## Suivi dans la recherche

**Aucune visite de suivi spécifique à la recherche n’est prévue.**

Le **suivi en pré-hospitalier** sera effectué par le médecin pré-hospitalier investigateur qui a inclus le patient.

Deux temps d’évaluation sont prévus :

- sur le lieu de survenue de l’ACEH à la fin de la prise en charge pré-hospitalière du patient, avant de l’emmener à l’hôpital ou de le déclarer décédé, les paramètres recueillis seront :
- Le retour à une activité cardiaque spontanée ;
- La dose totale d’adrénaline administrée ;
- Le nombre total de chocs électriques externes délivrés ;
- Le nombre de persistances et/ou récidives de trouble du rythme nécessitant un choc électrique externe.
- au moment de l’admission hospitalière du patient le cas échéant, les paramètres recueillis seront :
- La survie du patient à son arrivée à l’hôpital (présence ou absence d’une activité cardiaque spontanée efficace), qui est le critère d’évaluation principal de l’étude ;
- Les paramètres hémodynamiques à l’admission à l’hôpital.

**A la fin de l’intervention**, le patient, s’il est vivant, et le cas échéant après passage par la salle de cathétérisme cardiaque si celui-ci est indiqué, sera amené vers un service de réanimation où la prise en charge initiale sera poursuivie selon les pratiques habituelles du service. Le choix des traitements entrepris en réanimation ne sera pas impacté par la prise en charge pré-hospitalière.

Le **suivi à l’hôpital** sera alors effectué par les médecins hospitaliers accueillant le patient après la prise en charge pré-hospitalière. Deux temps d’évaluation de la survie et du pronostic neurologique (score *Cerebral Performance Category*) sont prévus :

- au moment de la sortie du patient de l’hôpital le cas échéant ;
- à 3 mois (J90) +/- 1 semaine après l’inclusion du patient.

Ces 2 évaluations pourront être effectuées à partir du ou des compte rendu(s) d’hospitalisation à la sortie du patient de l’hôpital et lors d’entretiens téléphoniques avec le patient lui-même ou un proche et/ou un médecin le prenant en charge à cette période (appel réalisé par un technicien d’études cliniques centralisé).

Lors de l’hospitalisation, le patient, ou le cas échéant les membres de la famille ou la personne de confiance, seront informés dès que possible de son inclusion dans la recherche. Son consentement si le patient est en état de consentir, ou le consentement des proches, sera demandé pour la poursuite de la recherche (article L1122-1-3 du Code de Santé Publique).

Les données recueillies lors du suivi seront des données anamnestiques, cliniques et de monitorage électrocardioscopique**. Aucun examen complémentaire supplémentaire en particulier électrocardioscopique, d’imagerie ou biologique ne sera effectué lors de ce suivi spécifiquement dans le cadre de cette recherche**. L’évaluation du score CPC sera considérée comme une donnée clinique.

Les coordonnées postales, électroniques, et téléphoniques du patient et d’un proche seront recueillies pour permettre le suivi des patients survivants à 3 mois. La commune de naissance sera également recueillie afin de vérifier le statut vital des patients et en cas de décès, sa cause (registres RNIPP et CépiDC) s’ils sont perdus de vue à 3 mois et qu’il n’a pas été possible de récupérer des informations auprès des proches.

## Visite de fin de la recherche

Aucune visite de fin de recherche spécifique à la recherche n’est prévue.

## Schéma récapitulatif de la chronologie de la recherche

La séquence des temps d’évaluation de la recherche est présentée sur le chronogramme de la figure 4.

- L’évaluation de l’éligibilité du patient se fait entre le début de la prise en charge du patient par l’équipe médicale pré-hospitalière et le 3^ème^ CEE.

- L’inclusion a lieu immédiatement après le 3^ème^ CEE, si le patient présente toujours une FV après ce CEE

- Selon l’évolution du patient durant sa prise en charge intra-hospitalière, la sortie de l’hôpital peut éventuellement survenir après la fin du 3^ème^ mois suivant l’ACEH. Mais l’évaluation se fera toujours au 3^ème^ mois si le patient est vivant, qu’il soit sorti de l’hôpital ou toujours hospitalisé.

**Figure 4 :** Chronologie de la recherche.

| **Visite** | **Inclusion en**  **pré-hospitalier** | **Admission en réanimation** | **Sortie de l’hôpital** | **Suivi**  **(3 mois)** |
| --- | --- | --- | --- | --- |
| **Critères d’inclusion - non inclusion** | **X** |  |  |  |
| **Inclusion** | **X** |  |  |  |
| **Information** |  | **X** |  |  |
| **Recueil du consentement de poursuite** |  | **X** |  |  |
| **Examen clinique** | **X** | **X** | **X** |  |
| **Traitement à l’étude** | **X** |  |  |  |
| **Evaluation de la mortalité** | **X** | **X** | **X** | **X** |
| **Evaluation du score CPC** |  |  | **X** | **X** |

**TABLEAU : récapitulatif de la chronologi*e* de la recherche**

## Durée prévue de participation des personnes, description de la chronologie et de la durée de la recherche.

La durée du traitement pour chaque sujet est de quelques secondes (temps de réalisation de l’injection IVD de chlorure de potassium).

La durée prévue de participation des patients est de 3 mois.

La durée prévue de la période d’inclusion est de 18 mois.

La durée totale de la recherche est de 21 mois.

## Distinction soin-recherche

La figure 3 présente l’algorithme de prise en charge du patient selon les recommandations internationales (fig. 3A), et dans le cas de cette recherche (fig. 3B).

En pratique, la différence essentielle entre ces 2 algorithmes est l’administration IVD de 20 mmol de chlorure de potassium après le 3^ème^ CEE, en lieu et place de la première administration d’amiodarone 300 mg IVD. En conséquence, dans le cadre de la recherche, si elles s’avèrent nécessaires en cas d’échec de l’injection de chlorure de potassium pour interrompre la FV réfractaire, les administrations d’amiodarone 300 mg IVD, puis 150 mg IVD, puis le relais par l’administration continue d’amiodarone ou le recours à la lidocaïne, seront simplement « retardées » de 2 CEE par rapport à la prise en charge qui aurait été effectuée stricto sensu selon les recommandations internationales.

**TABLEAU : Distinction entre les actes liés au « soin » et les actes ajoutés par la « recherche »**

| **Actes, procédures et traitements réalisés dans le cadre de la recherche** | **Actes, procédures et traitements liés aux soins** | **Actes, procédures et traitements ajoutés par la recherche** |
| --- | --- | --- |
| **Traitements** | première administration d’amiodarone 300 mg IVD | administration IVD de 20 mmol de chlorure de potassium après le 3^ème^ CEE |
| **Suivi** |  | évaluation du score CPC à la sortie de l’hôpital et à 3 mois |

# CRITERES D’ELIGIBILITE

## Critères d’inclusion

- Patient majeur (âge supérieur ou égal à 18 ans).

- Patient victime d’un arrêt cardiaque extra-hospitalier d’origine cardiaque présumée et présentant une fibrillation ventriculaire réfractaire malgré 3 chocs électriques externes.

- Patient bénéficiant d’un régime d’assurance maladie.

## Critères de non inclusion

- Grossesse avérée.

- Incapable majeur (patient sous tutelle ou curatelle).

- Patient n’ayant encore pas de voie veineuse fonctionnelle après réalisation des 3 chocs électriques externes.

## Modalités de recrutement

Les patients adultes victimes d’un arrêt cardiaque extra-hospitalier d’origine cardiaque présumée avec une fibrillation ventriculaire réfractaire malgré 3 chocs électriques externes seront consécutivement inclus par une équipe médicale pré-hospitalière d’un des 4 centres participants (SMUR, ou Ambulance de Réanimation pour la Brigade des Sapeurs-Pompiers de Paris).

*Compte tenu du plan expérimental choisi (phase II de Simon en 2 étapes) : 34 patients seront inclus en 1^ère^ étape, puis, si le traitement est considéré comme efficace, l’essai se poursuivra avec l’inclusion de 47 patients avec au total de 81 patients inclus.*

| Nombre total de sujets sélectionnés | 81 |
| --- | --- |
| Nombre de centres recruteurs | 4 |
| Période d’inclusion (mois) | 18 |
| Nombre de sujets / centre | 21 |
| **Nombre de sujets / centre / mois** | **1 à 2** |

## Règles d’arrêt

### Critères et modalités d’arrêt prématuré du traitement de la recherche

Le traitement étant administré lors d’une injection IVD effectuée en quelques secondes, aucun critère d’arrêt de traitement n’est par conséquent définissable.

Cependant, l’extravasation ou l’échec à l’injection impliquent la sortie du schéma expérimental pour le participant.

### Critères et modalités d’arrêt prématuré de la participation à la recherche d’un sujet

Tout sujet peut arrêter sa participation à la recherche, à n’importe quel moment et quelle qu’en soit la raison.

L’investigateur peut interrompre définitivement la participation d’un sujet à la recherche pour toute raison ayant un impact sur sa sécurité ou qui servirait au mieux les intérêts du sujet.

Si le sujet est perdu de vue, l’investigateur doit mettre tout en œuvre pour reprendre contact avec le sujet ou ses proches (et le documenter dans le dossier source) afin de savoir au moins si le sujet est vivant ou décédé

Le cahier d’observation doit lister les différentes raisons d’arrêt de participation à la recherche :

- Effet indésirable
- Autre problème médical
- Raison personnelle du sujet
- Retrait explicite de consentement
- Perdu de vue

En cas d’arrêt prématuré de la recherche d’un sujet, ou de retrait du consentement, les données le concernant recueillies avant l’arrêt prématuré pourront être utilisées.

En cas de décès du sujet, **avant le recueil du consentement de poursuite** auprès du sujet ou d’un membre de sa famille ou de la personne de confiance, les données le concernant qui auront été recueillies avant son décès pourront être utilisées.

### Suivi des sujets suite à un arrêt de participation à la recherche

Dans le cas où un arrêt prématuré de participation à la recherche devait survenir, le critère d’évaluation principal (survie du patient à son arrivée à l’hôpital avec une activité cardiaque spontanée efficace) et les critères d’évaluation secondaires seront évalués s’ils sont disponibles au moment de cet arrêt prématuré.

L’arrêt de participation d'un sujet ne changera en rien sa prise en charge habituelle par rapport à sa maladie.

En cas d’évènements indésirables graves lors de l’arrêt prématuré du traitement et de participation à la recherche du patient, ceux-ci devront être notifiés par l’investigateur au promoteur. La notification de l’évènement indésirable grave sera transmise par mail ([eig-vigilance.drc@aphp.fr](mailto:eig-vigilance.drc@aphp.fr)) au promoteur. L’évènement indésirable grave sera suivi jusqu’à sa résolution. Le comité de surveillance indépendant pourra préciser et/ou valider les modalités du suivi.

### Modalités de remplacement de ces sujets, le cas échéant

Dans le cas d’un arrêt prématuré de la recherche lié à un retrait de consentement, et dans le cas où le patient ou ses proches refuseraient explicitement l’utilisation de l’ensemble de ses données, le patient serait exclu de l’analyse et serait remplacé.

### Arrêt d’une partie ou de la totalité de la recherche

Le promoteur AP-HP ou l’Autorité Compétente (ANSM) peuvent interrompre prématurément de façon temporaire ou définitive toute ou une partie de la recherche, suite aux recommandations d’un Comité de Surveillance Indépendant dans les situations suivantes :

- en premier, en cas d’effets indésirables graves inattendus (SUSARS) nécessitant une réévaluation du rapport bénéfices/risques de la recherche ;
- en cas d’analyse intermédiaire : arrêt pour démonstration de l’inefficacité du traitement.

De même, des faits imprévus, de nouvelles informations relatives au produit, au vu desquels les objectifs de la recherche ne seront vraisemblablement pas atteints, peuvent amener le promoteur AP-HP ou l’Autorité Compétente (ANSM) à interrompre prématurément la recherche**.**

Le promoteur AP-HP se réserve le droit de suspendre définitivement les inclusions, à tout moment, s'il s'avère que les objectifs d’inclusion ne sont pas atteints.

En cas d’arrêt prématuré de la recherche pour des raisons de sécurité, la décision et la justification sont transmises par le promoteur AP-HP dans un délai de 15 jours à l’Autorité Compétente (ANSM) et au CPP, accompagné des recommandations du Comité de Surveillance Indépendant dans le cadre d’une modification substantielle.

# TRAITEMENT ADMINISTRE AUX PERSONNES SE PRETANT A LA RECHERCHE

## Description du ou des médicaments expérimentaux

### Médicament expérimental 1

Le médicament expérimental est le chlorure de potassium :

- dosage : ampoule de 20 ml de KCl à 10% solution pour perfusion

- posologie : 20 mmol de chlorure de potassium soit 14,9 ml de KCl à 10%, posologie arrondie à 15 ml

- durée de traitement : quelques secondes, correspondant à l’injection IVD

- modalités et voie d’administration : administration intra veineuse directe (IVD) flash

- conditionnement :

Le promoteur fournira les traitements expérimentaux sous forme de coffrets de 1 ampoule de 20 ml de KCl à 10%. Ce coffret patient contiendra également tous les dispositifs (seringue, aiguille) nécessaires à l’administration du traitement en urgence ; les coffrets sont étiquetés aux mentions réglementaires de la recherche par le Département Essai Clinique de l’AGEPS et ne nécessitent pas de conditions de conservation spécifiques.

## Description du ou des traitements auxiliaires (médicaments nécessaires à la réalisation de la recherche)

En dehors du chlorure de potassium, aucun autre médicament spécifiquement utile à la recherche n’est prévu dans cette étude.

Les seuls autres médicaments utilisés seront ceux pouvant être habituellement administrés dans le cadre de la prise en charge d’un ACEH par FV réfractaire, donc en particulier l’adrénaline par voie intraveineuse directe à la posologie de 1 mg, l’amiodarone par voie intraveineuse directe à la posologie de 300 mg puis de 150 mg, puis en IV continue à la posologie de 900 mg/j, et enfin la lidocaïne par voie intraveineuse directe à la posologie de 1 mg/kg.

## Description des éléments de traçabilité qui accompagnent le ou les médicaments expérimentaux

La dispensation sera réalisée par les pharmacies hospitalières et une mise en dotation sera réalisée dans les services.

Une vignette de traçabilité permettra à la PUI de tracer l’attribution du coffret en dotation.

Une vignette de traçabilité permettra de tracer l’attribution du coffret au patient sur son ordonnance.

## Traitements (médicamenteux, auxiliaires, chirurgicaux) autorisés et interdits, y compris les médicaments de secours

Tous les autres médicaments habituellement administrés dans le cadre de la prise en charge d’un ACEH par FV réfractaire sont autorisés.

Il n’y a pas de médicament interdit.

## Méthodes de suivi de l'observance au traitement

Les modalités de dispensation du KCl seront recueillies dans l’eCRF : volume administré en totalité ou non, difficultés éventuelles pendant l’injection.

# Evaluation de la securité – RISQUES ET CONTRAINTES AJOUTES PAR LA RECHERCHE

## Procédures mises en place en vue de l’enregistrement et de la notification des évènements indésirables

### Définitions

D’après l’article R.1123-46 du Code de la Santé Publique :

- **Evènement indésirable :**

Toute manifestation nocive survenant chez une personne qui se prête à une recherche impliquant la personne humaine, que cette manifestation soit liée ou non à la recherche ou au produit sur lequel porte cette recherche.

- **Effet indésirable :**

Evénement indésirable survenant chez une personne qui se prête à une recherche impliquant la personne humaine, lorsque cet événement est lié à la recherche ou au produit sur lequel porte cette recherche.

- **Effet indésirable d’un médicament expérimental :**

Toute réaction nocive et non désirée à un médicament expérimental, quelle que soit la dose administrée.

- **Evènement ou effet indésirable grave :**

Tout événement ou effet indésirable qui entraîne la mort, met en danger la vie de la personne qui se prête à la recherche, nécessite une hospitalisation ou la prolongation de l'hospitalisation, provoque une incapacité ou un handicap importants ou durables, ou bien se traduit par une anomalie ou une malformation congénitale, et s'agissant du médicament, quelle que soit la dose administrée.

- **Effet indésirable inattendu d’un médicament expérimental :**

Tout effet indésirable du produit dont la nature, la sévérité, la fréquence ou l'évolution ne concordent pas avec les informations de référence sur la sécurité mentionnées dans le résumé des caractéristiques du produit ou dans la brochure pour l'investigateur lorsque le produit n'est pas autorisé.

D’après l’article R.1123-46 du Code de la Santé Publique et l'avis aux promoteurs d'essais cliniques de médicaments (ANSM) :

- **Fait nouveau :**

Toute nouvelle donnée pouvant conduire à une réévaluation du rapport des bénéfices et des risques de la recherche ou du produit objet de la recherche, à des modifications dans l'utilisation de ce produit, dans la conduite de la recherche, ou des documents relatifs à la recherche, ou à suspendre ou interrompre ou modifier le protocole de la recherche ou des recherches similaires.

Pour les essais portant sur la première administration ou utilisation d'un produit de santé chez des personnes qui ne présentent aucune affection : tout effet indésirable grave.

Exemples :

a) toute augmentation cliniquement significative de la fréquence d’apparition d’un effet indésirable grave attendu ;

b) des suspicions d’effets indésirables graves inattendus survenus chez des participants ayant terminé l’essai et qui sont notifiés par l’investigateur au promoteur, ainsi que des rapports de suivi éventuels ;

c) tout fait nouveau concernant le déroulement de l’essai clinique ou le développement du médicament expérimental, lorsque ce fait nouveau est susceptible de porter atteinte à la sécurité des participants ;

d) les recommandations du comité de surveillance indépendant (CSI), le cas échéant, si elles sont pertinentes pour la sécurité des personnes ;

e) tout effet indésirable grave inattendu transmis au promoteur par un autre promoteur d’un essai clinique mené dans un pays tiers portant sur le même médicament.

### Rôles de l’investigateur

L’investigateur doit **évaluer pour chaque évènement indésirable sa gravité** et reporter tous les évènements indésirables graves et non graves dans le cahier d’observation (e-CRF)*.*

L’investigateur doit **documenter au mieux** les évènements indésirables graves et donner dans la mesure du possible, le diagnostic médical définitif.

L’investigateur doit **évaluer l’intensité** des évènements indésirables par des termes généralistes :

- - *Léger : toléré par le patient, n’interférant pas avec ses activités quotidiennes,*
  - *Modéré : suffisamment inconfortable pour altérer les activités quotidiennes,*
  - *Sévère : qui empêche les activités quotidiennes.*

L’investigateur doit **évaluer le lien de causalité** des évènements indésirables graves avec le médicament expérimental.

La méthode utilisée par l’investigateur, basée sur la méthode OMS (WHO Uppsala Monitoring Centre), repose sur les 4 termes de causalité suivants :

- Certain,
- Probable/plausible,
- Possible,
- Improbable (non exclu).

Leur définition est présentée dans le tableau suivant (extrait de *WHO-UMC causality categories*, version du 17/04/2012).

Tableau : *WHO-UMC causality categories* (extrait).

| **Causality term** | Assessment criteria* |
| --- | --- |
| **Certain** | - Event or laboratory test abnormality, with plausible time relationship to drug intake ** - Cannot be explained by disease or other drugs - Response to withdrawal plausible (pharmacologically, pathologically) - Event definitive pharmacologically or phenomenologically (i.e. an objective and specific medical disorder or a recognized pharmacological phenomenon) - Rechallenge satisfactory, if necessary |
| **Probable / Likely** | - Event or laboratory test abnormality, with reasonable time relationship to drug intake** - Unlikely to be attributed to disease or other drugs - Response to withdrawal clinically reasonable - Rechallenge not required |
| **Possible** | - Event or laboratory test abnormality, with reasonable time relationship to drug intake ** - Could also be explained by disease or other drugs - Information on drug withdrawal may be lacking or unclear |
| **Unlikely** | - Event or laboratory test abnormality, with a time to drug intake ** - that makes a relationship improbable (but not impossible) - Disease or other drugs provide plausible explanations |

*All points should be reasonably complied with

** Or study procedures

### Evènements indésirables graves nécessitant une notification sans délai par l’investigateur au promoteur

Selon l’article R.1123-49 du Code de la Santé Publique, l’investigateur notifie au promoteur sans délai à compter du jour où il en a connaissance tous les événements indésirables graves, survenus au cours d’une recherche mentionnée au 1o de l’article L.1121-1, à l’exception de ceux qui sont recensés dans le protocole et, le cas échéant, dans la brochure pour l’investigateur comme ne nécessitant pas de notification.

Un évènement indésirable grave présente l’un des critères suivants :

1. évènement qui entraîne la mort,
2. évènement qui met en danger la vie de la personne qui se prête à la recherche,
3. évènement qui nécessite une hospitalisation ou la prolongation de l'hospitalisation,
4. évènement qui provoque une incapacité ou un handicap importants ou durables,
5. évènement qui se traduit par une anomalie ou une malformation congénitale.

### Spécificités du protocole

#### Autres évènements nécessitant une notification sans délai par l’investigateur au promoteur

- *Exposition* ***in utero***

Toute grossesse découverte au cours de la recherche, même si elle n’est pas associée à un évènement indésirable, doit être notifiée au promoteur par l’investigateur sans délai à compter du jour où il en a connaissance.

La notification est à faire en cas d'exposition maternelle (cf RCP).

#### Evènements indésirables graves ne nécessitant pas une notification sans délai par l’investigateur au promoteur

Ces EIGs seront uniquement recueillis dans la section « évènements indésirables » dans le cahier d’observation.

- *Evolution naturelle et habituelle de la pathologie :*

Le critère d’évaluation principal est la survie du patient à l’arrivée à l’hôpital. Le taux de mortalité suite à un ACEH par FV réfractaire est de 75 à 80 % à la sortie de l’hôpital **[7]**. Les EIGs survenant après l’admission hospitalière ne seront donc pas notifiés sans délai.

De même, **les décès ne seront pas à notifier sans délai au promoteur mais seront recueillis dans le cahier d’observation.** Une extraction des décès du cahier d’observation sera réalisée tous les 3 mois par l’unité de recherche clinique et sera envoyée, sans nécessité de demande préalable, via le référent projet du Pôle Promotion au secteur Vigilance et aux membres du CSI.

- *Evènements indésirables susceptibles d’être liés aux traitements prescrits dans le cadre du soin pendant le suivi de la recherche*

Ces effets indésirables doivent être notifiés par l’investigateur au centre régional de pharmacovigilance dont il dépend.

### Période de notification sans délai des EIG par l’investigateur au promoteur

L’investigateur doit notifier sans délai au promoteur les évènements indésirables graves tels que définis dans la rubrique correspondante :

- à partir de la date de début de traitement par le médicament expérimental,
- jusqu’à l’admission à l’hôpital,
- sans limitation de temps, lorsque l’EIG est susceptible d’être dû au médicament expérimental.

### Modalités et délais de notification au promoteur

La notification initiale d’EIG fait l’objet d’un rapport écrit et signé par l’investigateur à l’aide d’un formulaire de notification des EIG spécifique à la recherche et prévu à cet effet (dans le cahier d’observation).

Chaque item de ce document doit être complété par l’investigateur pour permettre au promoteur d’effectuer une analyse pertinente.

La notification initiale d’un événement indésirable grave au promoteur doit être suivie rapidement par un (ou des) rapport(s) complémentaire(s) écrit(s) détaillé(s) permettant de suivre l’évolution du cas en vigilance ou de compléter les informations.

L’investigateur transmettra, dans la mesure du possible, tout document pouvant être utile au promoteur (comptes rendus médicaux, résultats biologiques, résultats d’examens complémentaires, etc). Ces documents devront être rendus anonymes. Par ailleurs, ils devront être complétés par les mentions suivantes : acronyme de la recherche, numéro et initiales du participant.

Tout évènement indésirable sera suivi jusqu’à sa complète résolution (stabilisation à un niveau jugé acceptable par l’investigateur ou retour à l’état antérieur) même si le participant est sorti de la recherche.

La notification initiale, les rapports de suivi d'EIG et tout autre document seront transmis au promoteur représenté par son secteur Vigilance, par mail ([eig-vigilance.drc@aphp.fr](mailto:eig-vigilance.drc@aphp.fr)). Il est à noter qu’il est possible de transmettre les EIG au secteur Vigilance par télécopie au 01 44 84 17 99 uniquement en cas de tentative infructueuse d’envoi des EIG par mail (afin d’éviter les doublons).

Dans le cas de cette recherche avec e-CRF :

- l’investigateur complète le formulaire de notification d’EIG dans l’e-CRF, le valide, l’imprime, le signe puis l'envoie par mail ;
- en cas d’impossibilité de connexion à l’e-CRF, l’investigateur complètera, signera et adressera le formulaire de notification d’EIG au secteur Vigilance. Dès que la connexion sera rétablie, il régularisera en complétant le formulaire de notification d’EIG dans l’e-CRF.

L’investigateur doit répondre à toute demande d’informations complémentaires émanant du promoteur.

Pour toute question relative à la notification d’un évènement indésirable, il est possible de contacter le secteur Vigilance par courriel : [vigilance.drc@aphp.fr](mailto:vigilance.drc@aphp.fr).

En cas d’exposition *in utero*, l’investigateur complète le « formulaire de notification et de suivi d’une grossesse apparue au décours d’une recherche ».

L’investigateur doit suivre la femme enceinte jusqu’au terme de la grossesse ou de son interruption et en notifier l’issue au promoteur avec ce formulaire.

Si l’issue de la grossesse entre dans le cadre de la définition des évènements indésirables graves (avortement spontané, interruption de grossesse, mort fœtale, anomalie congénitale, etc.), l’investigateur doit suivre les modalités de notification des EIG.

La notification initiale de grossesse, les rapports de suivi d'EIG et tout autre document seront transmis au promoteur selon les mêmes modalités que précisées ci-dessus.

## Rôles du promoteur

Le promoteur représenté par son secteur Vigilance évalue la sécurité de chaque médicament expérimental de façon continue, tout au long de la recherche.

### Analyse et déclaration des évènements indésirables graves

Le promoteur évalue :

- la **gravité** de tous les évènements indésirables qui lui sont rapportés.
- leur **lien de causalité** avec chaque médicament expérimental et avec les autres traitements éventuels.
- Tous les évènements indésirables graves pour lesquels l'investigateur et/ou le promoteur estiment qu'une relation de causalité avec le médicament expérimental peut être raisonnablement envisagée sont considérés comme des suspicions d'effets indésirables graves.
- le **caractère attendu ou inattendu** des effets indésirables graves.
- Tout effet indésirable grave dont la nature, la sévérité, la fréquence ou l’évolution ne concordent pas avec les informations de référence sur la sécurité mentionnées dans le résumé des caractéristiques du produit ou dans la brochure pour l'investigateur lorsque le produit n'est pas autorisé est considéré comme inattendu.
- L’évaluation du caractère attendu/inattendu d’un effet indésirable grave est effectuée par le promoteur représenté par son secteur Vigilance sur la base des informations décrites ci-dessous.
- Pour les évènements indésirables graves susceptibles d’être liés au médicament expérimental :

Il convient de se référer au RCP de la spécialité **« Chlorure de Potassium »**

<http://agence-prd.ansm.sante.fr/php/ecodex/frames.php?specid=63662572&typedoc=R&ref=R0320091.htm>

Le promoteur déclare toute suspicion d'effet indésirable grave inattendu (SUSAR), dans les délais réglementaires, auprès de l'Agence nationale de sécurité des médicaments et des produits de santé (ANSM):

- La déclaration initiale doit être réalisée sans délai à compter du jour où le promoteur en a eu connaissance dans le cas d’effet indésirable grave inattendu ayant entraîné la mort ou mis la vie en danger et dans un délai de 15 jours à compter du jour où le promoteur en a eu connaissance pour le cas des autres effets indésirables graves inattendus ;
- Toutes les informations complémentaires pertinentes doivent être déclarées par le promoteur sous forme de rapports de suivi, dans un délai de 8 jours calendaires à compter du moment où il dispose de ces informations.

Toute suspicion d'effet indésirable grave inattendu est déclarée également par voie électronique dans la base de données européenne Eudravigilance relative aux effets indésirables de médicaments mise en place par l’Agence européenne des médicaments (EMA).

Le promoteur informe tous les investigateurs concernés de toute donnée qui pourrait avoir un impact défavorable sur la sécurité des personnes qui se prêtent à la recherche.

### Analyse et déclaration des autres données de sécurité

Il s’agit de toute nouvelle donnée pouvant conduire à une réévaluation du rapport des bénéfices et des risques de la recherche ou du produit objet de la recherche, à des modifications dans l’utilisation de ce produit, dans la conduite de la recherche, ou des documents relatifs à la recherche, ou à suspendre ou interrompre ou modifier le protocole de la recherche ou des recherches similaires. Pour les essais portant sur la première administration ou utilisation d'un produit de santé chez des personnes qui ne présentent aucune affection : tout effet indésirable grave.

Le promoteur informe sans délai l'autorité compétente et le comité de protection des personnes des faits nouveaux et, le cas échéant, des mesures prises, à compter du jour où il en a eu connaissance.

A la suite de la déclaration initiale relative à un fait nouveau, le promoteur adresse aux autorités compétentes sous forme d’un rapport de suivi du fait nouveau, toute information complémentaire pertinente relative à ce fait nouveau dans un délai de 8 jours maximum à compter du moment où il dispose de ces informations.

### Rapport annuel de sécurité

Le promoteur doit établir une fois par an pendant toute la durée de la recherche un rapport annuel de sécurité (*Development Safety Update Report* - DSUR) comprenant notamment :

- une analyse de la sécurité des personnes qui se prêtent à la recherche,

- une description des patients inclus dans la recherche (caractéristiques démographiques, etc.),

- une liste de toutes les suspicions d'effets indésirables graves survenus pendant la période couverte par le rapport,

- les tableaux de synthèse de tous les évènements indésirables graves survenus depuis le début de la recherche.

Le rapport est transmis dans les 60 jours après la date anniversaire correspondant à la date d'autorisation de la recherche par l'ANSM.

### Comité de Surveillance Indépendant

Un Comité de Surveillance Indépendant (CSI) est mis en place par le promoteur dans le cadre de cette recherche. Il a comme principale mission d’être un comité de suivi des données de sécurité. Il a également pour mission le suivi des données.

Une réunion préliminaire du CSI est prévue avant la première inclusion du premier participant, idéalement avant la soumission du protocole à l’autorité compétente et au CPP.

L’ensemble des missions ainsi que les modalités précises de fonctionnement du CSI sont décrites dans la charte du CSI de l’étude.

Les membres du CSI sont :

- Pr B. CHOLLEY : Anesthésie-Réanimation-HEGP

- Dr T. LOEB : Anesthésie-Réanimation-SAMU 92

- Pr F. CANOUÏ-POITRINE : Statisticienne-UPEC-MONDOR

- Pr E. WIEL : Anesthésie-Réanimation et Médecine d’Urgence-CHU LILLE

Le fonctionnement du CSI sera conforme aux procédures du promoteur. Le CSI a une fonction consultative, le promoteur reste décisionnaire.

# gestion des donnees

## Modalités de recueil des données

Lors de l’inclusion d’un patient, au retour de l’intervention, le médecin investigateur urgentiste enverra à l’URC un fax signalant l’inclusion d’un sujet.

Les coordonnées postales, électroniques, et téléphoniques du patient et d’un proche seront recueillies et envoyées au technicien d’études cliniques formé au protocole pour permettre le suivi des patients survivants à 3 mois.

La commune de naissance sera recueillie afin de vérifier le statut vital des patients s’ils sont perdus de vue à 3 mois.

Les données concernant la prise en charge pré-hospitalière du patient seront recueillies par l’équipe du service d’urgence pré-hospitalier qui aura inclus le patient.

Les données concernant le suivi du patient à l’hôpital seront recueillies par le service de réanimation qui aura pris en charge le patient.

Le technicien d’études cliniques contactera les patients survivants à 3 mois par téléphone pour l’évaluation du score CPC.

Les données de prise en charge pré-hospitalière puis de suivi à l’hôpital seront recueillies dans un cahier d’observation papier puis saisies dans l’eCRF.

## Identification des données recueillies directement dans les CRF qui seront considérées comme données-source

Les données recueillies lors de l’appel à 3 mois seront saisies directement dans l’eCRF.

## Droit d’accès aux données et documents source

### Accès aux données

Conformément aux BPC :

- le promoteur est chargé d’obtenir l’accord de l’ensemble des parties impliquées dans la recherche afin de garantir l’accès direct à tous les lieux de déroulement de la recherche, aux données source, aux documents source et aux rapports dans un but de contrôle de qualité et d’audit par le promoteur, ou d’inspection par l’autorité compétente

- les investigateurs mettront à disposition des personnes chargées du suivi, du contrôle de qualité ou de l'audit ou de l’inspection de la recherche interventionnelle impliquant la personne humaine, les documents et données individuelles strictement nécessaires à ce contrôle, conformément aux dispositions législatives et réglementaires en vigueur (articles L.1121-3 et R.5121-13 du Code de la Santé Publique).

### Documents source

Les documents source étant définis comme tout document ou objet original permettant de prouver l'existence ou l'exactitude d'une donnée ou d'un fait enregistré au cours de la recherche seront conservés selon la réglementation en vigueur par l'investigateur ou par l'hôpital s'il s'agit d'un dossier médical hospitalier.

Le document source sera, pour la partie pré-hospitalière de l’étude, la feuille d’intervention pré-hospitalière (SMUR ou Ambulance de Réanimation de la Brigade de Sapeurs Pompiers de Paris) et le CRF papier en dupliqué (1 feuillet sera conservé dans le service d’urgences pré-hospitalier qui a inclus le patient et l’original déliassé sera transmis par voie postale ou fax au technicien d’études cliniques pour saisie).

Les données recueillies directement dans le CRF sont des données spécifiques à la recherche.

Pour la phase hospitalière, les données seront recueillies via un eCRF. L’eCRF sera complété par un technicien d’étude clinique ou un investigateur sous la responsabilité de l’investigateur principal du service de réanimation accueillant le patient. Un dossier médical sera établi dès l’admission du patient à l’hôpital, et considéré alors comme dossier source pour le recueil des données de la phase hospitalière.

### Confidentialité des données

Les personnes chargées du contrôle de qualité d’une recherche impliquant la personne humaine (article L.1121-3 du code de la santé publique), prendront toutes les précautions nécessaires en vue d'assurer la confidentialité des informations relatives aux médicaments expérimentaux, à la recherche, aux personnes qui s'y prêtent et notamment en ce qui concerne leur identité ainsi qu’aux résultats obtenus.

Ces personnes, au même titre que les investigateurs eux-mêmes, sont soumises au secret professionnel (selon les conditions définies par les articles 226-13 et 226-14 du code pénal).

Pendant la recherche impliquant la personne humaine et à son issue, les données recueillies sur les personnes qui s’y prêtent et transmises au promoteur par les investigateurs (ou tous autres intervenants spécialisés) seront rendues non identifiantes. Elles ne doivent en aucun cas faire apparaître en clair les noms des personnes concernées ni leur adresse.

Seules les initiales du nom et du prénom seront enregistrées, accompagnées d’un numéro codé propre à la recherche indiquant l’ordre d’inclusion des sujets.

Le promoteur s’assurera que chaque personne qui se prête à la recherche a donné son accord par écrit pour l’accès aux données individuelles la concernant et strictement nécessaires au contrôle de qualité de la recherche.

## Traitement des données et conservation des documents et des données

### Identification du responsable et du lieu de la gestion du traitement des données

La gestion de la base de données cliniques sera sous la responsabilité de l’URC Paris Descartes – Necker - Cochin.

### Saisie des données

La saisie des données sur le cahier d’observation sera réalisée par les investigateurs*.*

La saisie des données sera réalisée sur un support électronique via un navigateur internet (eCRF, cleanWEB par la société Telemedecine technologies).

## Propriété des données

L’AP-HP est propriétaire des données et aucune utilisation ou transmission à un tiers ne peut être effectuée sans son accord préalable.

# aspects statistiques

## Hypothèses de calcul du nombre de sujets nécessaires et résultat

Le plan expérimental choisi est un plan de phase II en 2 étapes selon Simon (plan optimum). Cette procédure permet de tester les hypothèses suivantes :

- H0 : p≤ p0 (hypothèse nulle : taux d’efficacité insuffisant du traitement par KCl)

- H1 : p> p0 (hypothèse alternative : taux d’efficacité suffisant du traitement par KCl)

La probabilité p0 représente le taux d’efficacité théorique au-dessous duquel le traitement expérimental est jugé inintéressant. On doit également fixer un taux d’efficacité théorique que l’on désire absolument mettre en évidence, s’il existe (p1 ou taux de réponse cible).

Pour mettre en évidence une efficacité de 50% (p1) avec une efficacité minimale de 35% (p0, survie observée du groupe amiodarone dans Kudenchuk **[7]**), il est nécessaire d’inclure 81 patients, avec un risque alpha de 10% et une puissance de 90%.

34 patients seront inclus en 1^ère^ étape. Si 12 succès sont observés ou moins, l’essai ne sera pas poursuivi et on conclura à l’inefficacité du KCl. Si au moins 13 succès sont observés, l’essai se poursuivra avec l’inclusion de 47 nouveaux patients en 2^ème^ étape.

Le traitement sera considéré comme efficace, selon ces hypothèses, si au moins 34 succès sont observés au total sur les 81 patients inclus.

## Description des méthodes statistiques prévues y compris du calendrier des analyses intermédiaires prévues

L’analyse sera effectuée à l’Unité de Recherche Clinique Paris-Descartes Necker Cochin sous la responsabilité du Dr Caroline Elie à l’aide du logiciel R (http://cran.r-project.org/).

Une analyse descriptive des caractéristiques cliniques des patients à l’inclusion sera tout d’abord effectuée. Les données quantitatives seront exprimées sous forme de moyenne ± écart type ou de médiane [minimum-maximum], et sous forme d’effectifs et de pourcentages pour les données qualitatives.

Analyse du critère de jugement principal

Le critère d’évaluation principal est la survie du patient à son arrivée à l’hôpital (avec une activité cardiaque spontanée efficace.

Comme cela a déjà été précisé dans la justification de la taille d’échantillon, l’efficacité du traitement sera démontrée à la fin de la 2^ème^ étape, si au moins 34 patients présentent une activité cardiaque spontanée efficace à l’arrivée à l’hôpital, parmi la totalité des 81 patients inclus.

Le pourcentage de patients en succès sera calculé en fin d’étape 1 si le nombre de succès n’est pas compatible avec la poursuite de l’étude ou à la fin de l’étape 2 dans le cas contraire. L’intervalle de confiance à 95% de ce pourcentage sera estimé à partir d’une loi binomiale exacte.

Analyse des critères de jugement secondaires

Les données de tolérance seront décrites en termes de fréquence et pourcentage de survenue.

De même, les autres critères de jugement secondaires quantitatifs seront décrits sous forme de moyenne ± écart type ou de médiane [minimum-maximum], et les autres critères de jugement secondaires qualitatifs sous forme d’effectifs et de pourcentages.

La survie des patients à 3 mois pourra être décrite globalement à l’aide de courbes de Kaplan-Meier.

# contrôle ET ASSURANCE DE LA QUALITE

Chaque projet de recherche impliquant la personne humaine pris en charge par l’AP-HP est classé selon le risque prévisionnel encouru par les personnes se prêtant aux recherches grâce à la classification des recherches interventionnelles impliquant la personne humaine à promotion AP-HP

## Organisation générale

Le promoteur doit s’assurer de la sécurité et du respect des personnes qui ont accepté de participer à la recherche. Il doit mettre en place un système d'assurance qualité permettant de surveiller au mieux le déroulement de la recherche dans les centres investigateurs.

A cet effet, le promoteur mandate des Attachés de Recherche Clinique (ARC) qui ont pour mission principale d’effectuer des visites régulières de suivi dans les lieux de recherche après avoir effectué les visites d’ouvertures.

Les objectifs du suivi de la recherche, tels que définis dans les Bonnes Pratiques Cliniques, (BPC §5.18.1) sont de vérifier que :

• le droit, la sécurité et la protection des personnes qui se prêtent à la recherche sont satisfaits,

• les données rapportées sont exactes, complètes et cohérentes avec les documents sources,

• la recherche est conduite conformément au protocole en vigueur, aux BPC et aux dispositions législatives et réglementaires en vigueur.

### Stratégie d’ouverture des centres

La stratégie d’ouverture des centres mise en place pour cette recherche est déterminée grâce au plan de monitoring adapté.

Les ouvertures se feront sur site.

### Etendue du monitoring des centres

Dans le cas de cette recherche à risque **D,** le choix d’un niveau de monitoring adapté a été pondéré en fonction de la complexité, de l’impact et du budget de la recherche. A cet effet, le promoteur en accord avec l’investigateur coordonnateur a déterminé le score logistique et impact qui a permis d’obtenir le niveau de monitoring à mettre en place sur la recherche : niveau **élevé.**

## Contrôle de qualité

Un Attaché de Recherche Clinique (ARC) mandaté par le promoteur s’assurera de la bonne réalisation de la recherche, du recueil des données générées par écrit, de leur documentation, enregistrement et rapport, en accord avec les Procédures Opératoires Standard mises en application au sein de la DRCI et conformément aux Bonnes Pratiques Cliniques ainsi qu’aux dispositions législatives et réglementaires en vigueur.

L'investigateur et les membres de son équipe acceptent de se rendre disponibles lors des visites de Contrôle de qualité effectuées à intervalles réguliers par l’Attaché de Recherche Clinique. Lors de ces visites, les éléments suivant seront revus selon le niveau de monitoring:

- consentement écrit ;
- respect du protocole de la recherche et des procédures qui y sont définies ;
- qualité des données recueillies dans le cahier d'observation : exactitude, données manquantes, cohérence des données avec les documents "source" ;
- gestion des traitements utilisés.

## Cahier d’observation

Toutes les informations requises par le protocole doivent être consignées sur les cahiers d’observation et une explication doit être apportée pour chaque donnée manquante. Les données devront être recueillies au fur et à mesure qu'elles sont obtenues, et transcrites dans ces cahiers de façon nette et lisible.

Les données erronées relevées sur les cahiers d'observation seront barrées et les nouvelles données seront copiées, à côté de l'information barrée, accompagnées des initiales, de la date et éventuellement d’une justification par l’investigateur ou la personne autorisée qui aura fait la correction.

Le remplissage du cahier d'observation via internet par l'investigateur et/ou un technicien d’études cliniques permet ainsi à l'ARC de visualiser rapidement et à distance les données. L'investigateur est responsable de l'exactitude, de la qualité et de la pertinence de toutes les données saisies. De plus, lors de leurs saisies, ces données sont immédiatement vérifiées grâce à des contrôles de cohérence. A ce titre, l’investigateur doit valider toute modification de valeur dans le CRF. Ces modifications font l'objet d'un *audit trail*. Une justification peut éventuellement être intégrée en commentaire.

Une impression papier sera demandée en fin d'étude, authentifiée (datée et signée) par l'investigateur. Une copie du document authentifié à destination du promoteur devra être archivée par l’investigateur.

## Gestion des non conformités

Tout évènement survenant suite au non-respect du protocole, des procédures opératoires standardisées, des bonnes pratiques cliniques ou des dispositions législatives et réglementaires en vigueur par un investigateur ou toute autre personne impliquée dans la conduite de la recherche doit faire l’objet d’une déclaration de non-conformité au promoteur.

Ces non conformités seront gérées conformément aux procédures du promoteur.

## Audit / inspections

Les investigateurs s’engagent à accepter les audits d’assurance qualité effectués par le promoteur ainsi que les inspections effectuées par les autorités compétentes. Toutes les données, tous les documents et rapports peuvent faire l'objet d'audits et d'inspections réglementaires sans que puisse être opposé le secret médical*.*

Un audit peut être réalisé à tout moment par des personnes mandatées par le [promoteur](http://www.chusa.jussieu.fr/urcest/sous_cadre.php?fich=Lexique/new_index.php?isphp=0&fich=EC/legislation/DispositionslegislativesPromoteur.htm) et indépendantes des responsables de la recherche. Il a pour objectif de s'assurer de la qualité de la recherche, de la validité de ses résultats et du respect de la loi et des règlementations en vigueur.

Les personnes qui dirigent et surveillent la recherche acceptent de se conformer aux exigences du promoteur et à l’autorité compétente en ce qui concerne un audit ou une inspection de la recherche.

L’audit pourra s’appliquer à tous les stades de la recherche, du développement du protocole à la publication des résultats et au classement des données utilisées ou produites dans le cadre de la recherche.

## Engagement de responsabilités de l’Investigateur Principal

Avant de démarrer la recherche, chaque investigateur fournira au représentant du promoteur de la recherche son curriculum vitæ personnel actualisé, daté de moins d’un an et signé, comportant son numéro RPPS. Le CV devra comprendre les participations antérieures à des recherches et les formations liées à la recherche clinique.

Chaque investigateur s'engagera à respecter les obligations de la loi et à mener la recherche selon les BPC, en respectant les termes de la déclaration d'Helsinki en vigueur.

L’investigateur principal de chaque centre participant signera un engagement de responsabilités (document type DRCI) qui sera remis au représentant du promoteur.

Les investigateurs et leurs collaborateurs signeront un formulaire de délégation de fonctions précisant le rôle de chacun et fourniront leur CV.

# ASPECTS ETHIQUES ET LEGAUX

## Modalités d’information et de recueil du consentement des personnes se prêtant à la recherche

Conformément à l’article L1122-1-3 du CSP, en cas de recherches impliquant la personne humaine à mettre en œuvre dans des situations d’urgence qui ne permettent pas de recueillir le consentement préalable de la personne qui y sera soumise, le protocole présenté à l’avis du Comité de Protection des Personnes (CPP) prévoit que le consentement de cette personne n’est pas recherché et que seul est sollicité celui des membres de sa famille ou celui de la personne de confiance mentionnée à l’article L. 1111-6 dans les conditions prévues à l’article L. 1122-1-1, s’ils sont présents.

**Le protocole prévoit une dérogation à cette obligation dans le cas d’une urgence vitale immédiate, ce qui est le cas de notre étude. Cette dérogation est donc sollicitée auprès du CPP**. Le patient, ou le cas échéant, les membres de la famille ou la personne de confiance seront informés dès que possible et son (leur) consentement lui (leur) sera demandé pour la poursuite éventuelle de cette recherche. Ils peuvent également s’opposer à l’utilisation des données concernant la personne dans le cadre de cette recherche.

Conformément à l’article 57 de la loi n° 2018-493 du 20 juin 2018 relative à la protection des données personnelles, **les informations concernant les personnes décédées pourront faire l’objet d’un traitement de données, sauf si le patient a, de son vivant, exprimé son refus.**

Un exemplaire du document d’information et du formulaire de consentement daté et signé par la personne qui se prête à la recherche, ou par un membre de la famille le cas échéant, ainsi que par l’investigateur principal ou le médecin qui le représente est remis à la personne ou à un membre de la famille le cas échéant. L’investigateur principal ou le médecin qui le représente en conservera un exemplaire.

Un exemplaire sera placé en fin d’étude dans une enveloppe inviolable scellée regroupant l’ensemble des formulaires de consentement, celle-ci sera archivée par le promoteur.

En outre, l’investigateur précisera dans le dossier médical du patient la participation de celui-ci à la recherche, les modalités de recueil du consentement ainsi que les modalités de la délivrance de l’information en vue de le recueillir. Il conserve un exemplaire du formulaire du recueil du consentement de la personne daté et signé.

Le patient sera informé qu’en l’absence de réponse à l’appel à 3 mois, la commune de naissance pourra être contactée en vue du recueil du statut vital (RNIPP / CépiDC)

## Interdiction pour la personne de participer à une autre recherche ou période d’exclusion prévue à l’issu de la recherche, le cas échéant

Pendant sa durée de participation, le sujet ne peut pas participer à un autre protocole de recherche interventionnelle impliquant la personne humaine.

A la fin de la participation du sujet, il n’y a pas de période d’exclusion.

## Autorisation des lieux

La recherche se déroule dans des sites extra-hospitaliers ou services de soins sur des personnes présentant une condition clinique pour laquelle les services sont compétents et qui nécessite des actes pratiqués habituellement dans le cadre de leurs activités. Par conséquent, il n’est pas nécessaire d’avoir une autorisation de lieu spécifique pour la recherche.

## Obligations légales

### Rôle du promoteur

L’Assistance Publique - Hôpitaux de Paris (AP-HP) est le promoteur de cette recherche et par délégation la Délégation à la Recherche Clinique et à l'Innovation (DRCI) en assure les missions, conformément à l’article L.1121-1 du code de la santé publique. l'Assistance Publique - Hôpitaux de Paris se réserve le droit d'interrompre la recherche à tout moment pour des raisons médicales ou administratives ; dans cette éventualité, une notification sera fournie à l'investigateur

### Demande d’avis au comité de protection des personnes CPP

L’AP-HP en tant que promoteur obtient pour la recherche impliquant la personne humaine portant sur un médicament à usage humain, préalablement à sa mise en œuvre l’avis favorable du CPP concerné, dans le cadre de ses compétences et conformément aux dispositions législatives et réglementaires en vigueur.

### Demande d’autorisation à l’ANSM

L’AP-HP en tant que promoteur obtient pour la recherche impliquant la personne humaine portant sur un médicament à usage humain, préalablement à sa mise en œuvre l’autorisation de l’ANSM, dans le cadre de ses compétences et conformément aux dispositions législatives et réglementaires en vigueur.

### Démarches relatives à la réglementation informatiques et libertés

Le fichier informatique utilisé pour cette recherche est mis en œuvre conformément à la règlementation française (loi Informatique et Libertés modifiée) et européenne (Règlement Général sur la Protection des Données –RGPD).

Cette recherche n’entre pas dans le cadre de la « Méthodologie de Référence » (MR-001) de la CNIL car l’inclusion se fait dans le cadre d'une urgence sans recueil de consentement à l’inclusion.

Préalablement à la mise en œuvre du traitement des données nécessaires à la réalisation de la recherche, le promoteur devra obtenir l’autorisation de la CNIL.

### Modifications de la recherche

Toute modification substantielle apportée au protocole par l’investigateur coordonnateur, devra être transmise au promoteur pour approbation. Après cet accord, le promoteur devra obtenir préalablement à sa mise en œuvre un avis favorable du CPP et une autorisation de l’ANSM dans le cadre de leurs compétences respectives.

La note d’information et le formulaire de consentement pourront être révisés si nécessaire, notamment en cas de modification substantielle de la recherche ou de la survenue d’effets indésirables.

### Rapport final de la recherche

Le rapport final de la recherche impliquant la personne humaine mentionné à l’article R1123-67 du CSP est établi et signé par le promoteur et l’investigateur. Un résumé du rapport rédigé selon le plan de référence de l’autorité compétente doit être transmis à l’autorité compétente dans un délai d’un an, après la fin de la recherche, correspondant au terme de la participation de la dernière personne qui se prête à la recherche.

### Archivage

Les documents spécifiques d’une recherche interventionnelle impliquant la personne humaines portant sur un médicament à usage humain seront archivés par l’investigateur et le promoteur pour une durée de 15 ans après la fin de la recherche.

Cet archivage indexé comporte notamment :

- Une enveloppe scellée pour l’investigateur contenant un exemplaire de toutes les notes d’information et les formulaires de consentement signés de toutes les personnes du centre ayant participé à la recherche ;
- Une enveloppe scellée pour le promoteur contenant un exemplaire de toutes les notes d’information et les formulaires de consentement signés de toutes les personnes du centre ayant participé à la recherche ;
- Les classeurs « recherche » pour l’Investigateur et le promoteur comprenant (liste non exhaustive) :
- les versions successives du protocole (identifiées par le n° et la date de version), ses annexes,
- les autorisations de l’ANSM et les avis du CPP,
- les courriers de correspondance,
- la liste ou registre d’inclusion,
- les annexes spécifiques à la recherche,
- le rapport final de la recherche.
- Les documents de recueil des données

# Financement et assurance

## Source de financement

Ministère de la santé – PHRC inter-régional 2018

## Assurance

Le Promoteur, souscrit pour toute la durée de la recherche une assurance garantissant sa propre responsabilité civile ainsi que celle de tout médecin impliqué dans la réalisation de la recherche. Il assure également l'indemnisation intégrale des conséquences dommageables à la recherche pour la personne qui s'y prête et ses ayants droit, sauf preuve à sa charge que le dommage n'est pas imputable à sa faute ou à celle de tout intervenant, sans que puisse être opposé le fait d'un tiers ou le retrait volontaire de la personne qui avait initialement consenti à se prêter à la recherche.

L'Assistance Publique - Hôpitaux de Paris (AP-HP) a pris une assurance auprès de la compagnie HDI-GLOBAL SE par l’intermédiaire de BIOMEDIC-INSURE pour toute la durée de la recherche, garantissant sa responsabilité civile ainsi que celle de tout intervenant (médecin ou personnel impliqué dans la réalisation de la recherche), conformément à l’article L.1121-10 du CSP.

# regles relatives a la publication

L’APHP devra obligatoirement être mentionnée dans les affiliations du ou des auteurs des publications qui résulteront de cette recherche et mentionner le promoteur AP-HP (DRCI) et la source de financement (cf. ci-dessous les modalités d’affiliation et de mention du promoteur et du financeur).

## Mention de l’affiliation de l’AP-HP pour les projets promus par l’AP-HP

Si un auteur a plusieurs affiliations, l’ordre dans lequel sont citées les institutions (AP-HP, Université, INSERM…) n’a pas d’importance. Chacune de ces affiliations doit être identifiée par une adresse séparée par un point virgule (;). L’institution AP-HP doit apparaître sous le sigle « AP-HP » en premier dans l’adresse suivi précisément par : AP-HP, hôpital, service, ville, code postal, France.

## Mention du promoteur AP-HP (DRCI) dans les ”acknowledgments” du manuscrit

”The sponsor was Assistance Publique – Hôpitaux de Paris (Délégation à la Recherche Clinique et à l'Innovation)”.

## Mention du financeur dans les ”acknowledgments” du manuscrit

*“*The study was funded by a grant from Programme Hospitalier de Recherche Clinique – PHRC-IR 2018 (Ministère de la Santé)”.

**Cette recherche est enregistrée sur le site http://clinicaltrials.gov/ sous le n° *numéro d’enregistrement*** *(ajouter le n° numéro d’enregistrement quand la recherche est enregistrée)*.

# bibliographie

| **1** | Gueugniaud PY, Bertrand C, Savary D, Hubert H. L’arrêt cardiaque en France : pourquoi un registre national ? Presse Med 2011;40(6):634-8. |
| --- | --- |
| **2** | Link MS, Berkow LC, Kudenchuk PJ, et al. Part 7: Adult Advanced Cardiovascular Life Support: 2015 American Heart Association Guidelines Update for Cardiopulmonary Resuscitation and Emergency Cardiovascular Care. Circulation 2015 Nov 3;132(18 Suppl 2):S444-64. |
| **3** | Sasson C, Rogers MA, Dahl J, Kellermann AL. Predictors of survival from out-of-hospital cardiac arrest: a systematic review and meta-analysis. Circ Cardiovasc Qual Outcomes 2010 ; 3 : 63-81. |
| **4** | Daya MR, Schmicker RH, Zive DM, et al ; Resuscitation Outcomes Consortium Investigators. Out-of-hospital cardiac arrest survival improving over time: Results from the Resuscitation Outcomes Consortium (ROC). Resuscitation 2015 ; 91 : 108-15. |
| **5** | American Heart Association. [2015 American Heart Association Guidelines Update for Cardiopulmonary Resuscitation and Emergency Cardiovascular Care](http://circ.ahajournals.org/content/132/18_suppl_2/S315). Circulation 2015 ; 132 : S 315-S589. |
| **6** | European Resuscitation Council. European Resuscitation Council Guidelines for Resuscitation 2015. Resuscitation 2015, 95 : 1-311. |
| **7** | Kudenchuk PJ, Brown SP, Daya M, et al.; Resuscitation Outcomes Consortium Investigators. Amiodarone, Lidocaine, or Placebo in Out-of-Hospital Cardiac Arrest. N Engl J Med 2016 May 5;374(18):1711-22. |
| **8** | Menasché P. Protection myocardique. In : Janvier G, Lehot JJ. Circulation extracorporelle : principes et pratique. Rueil-Malmaison, France : Arnette, 2000 : 77-96. |
| **9** | Weinstock L, Clark JH. Successful treatment of ventricular fibrillation with intracardiac potassium chloride. Am J Cardiol 1961 May;7:742-5. |
| **10** | Robicsek F. Biochemical termination of sustained fibrillation occurring after artificially induced ischemic arrest. J Thorac Cardiovasc Surg 1984 Jan;87(1):143-5. |
| **11** | Øvrum E, Tangen G, Holen EA, Ringdal MA, Istad R. Conversion of postischemic ventricular fibrillation with intraaortic infusion of potassium chloride. Ann Thorac Surg 1995 Jul;60(1):156-9. |
| **12** | Almdahl SM, Damstuen J, Eide M, Mølstad P, Halvorsen P, Veel T. Potassium-induced conversion of ventricular fibrillation after aortic declamping. Interact Cardiovasc Thorac Surg 2013 Feb;16(2):143-50. |
| **13** | Liakopoulos OJ, Allen BS, Buckberg GD, Hristov N, Tan Z, Villablanca JP, Trummer G. Resuscitation after prolonged cardiac arrest: role of cardiopulmonary bypass and systemic hyperkalemia. Ann Thorac Surg 2010 Jun;89(6):1972-9. |
| **14** | Watanabe G, Yashiki N, Tomita S, Yamaguchi S. Potassium-induced cardiac resetting technique for persistent ventricular tachycardia and fibrillation after aortic declamping. Ann Thorac Surg 2011 Feb;91(2):619-20. |
| **15** | Jouffroy R, Lamhaut L, Philippe P, An K, Carli P, Vivien B. A new approach for treatment of refractory ventricular fibrillation allowed by extra corporeal life support (ECLS)? Resuscitation 2014 Aug;85(8):e118. |
| **16** | Koller ML, Riccio ML, Gilmour RF Jr. Effects of [K(+)](o) on electrical restitution and activation dynamics during ventricular fibrillation. Am J Physiol Heart Circ Physiol. 2000 Dec;279(6):H2665-72. |
| **17** | Pandit SV, Warren M, Mironov S, Tolkacheva EG, Kalifa J, Berenfeld O, Jalife J. Mechanisms underlying the antifibrillatory action of hyperkalemia in Guinea pig hearts. Biophys J. 2010 May 19;98(10):2091-101. |
| **18** | Almdahl SM, Veel T, Eide M, Damstuen J, Halvorsen P, Mølstad P. Postcardioplegia ventricular fibrillation: no impact on subsequent survival. Scand Cardiovasc J 2014 Aug;48(4):249-54. |
| **19** | Houiller P, Paillard M. Régulation du métabolisme du potassium. Désordres acido-basiques et hydro-électrolytiques. Paris : Arnette ; 1997. p. 221-51. |
| **20** | Brenner BM, Berliner RW. The transport of potassium. In: Orloff J, Berliner RW, eds Renal Physiology, handbook of physiology. Bethesda: American Physiological Society; 1973. p. 497-519. |
| **21** | Garcia P, Belhoula M, Grimaud D. Les dyskaliémies. Consensus d’actualisation SFAR 1999. |
| **22** | Paillard M, Houiller P. Bilan de potassium et kaliémie. In : Paillard M, éd. Physiologie rénale et désordres électrolytiques. Paris : Hermann ; 1992. p. 153-83. |

# LISTE des addenda

## Liste des Investigateurs

| **N° centre** | **Coordonnées du lieu de recherche** | **Titre** | **Prénom - Nom** | **Téléphone / e-mail / Fax** |
| --- | --- | --- | --- | --- |
| 001 | SAMU de Paris – SMUR Necker | Pr | Benoît VIVIEN | Tél : 01 44 49 23 67  Mail : [benoit.vivien@aphp.fr](mailto:benoit.vivien@aphp.fr)  Fax : 01 44 49 23 25 |
| 002 | SAMU de Paris – SMUR Pitié Salpêtrière | Dr | Patrick ECOLLAN | Tél : 01 42 16 76 60  Mail : [patrick.ecollan@aphp.fr](mailto:patrick.ecollan@aphp.fr)  Fax : 01 42 16 76 76 |
| 003 | Brigade de Sapeurs Pompiers de Paris | Pr | Bertrand PRUNET | Tél : 01 56 79 67 53  Mail : [bertrand.prunet@pompiersparis.fr](mailto:bertrand.prunet@pompiersparis.fr)  Fax : 01 56 79 67 67 |
| 004 | SAMU 94 – SMUR Henri Mondor | Dr | Charlotte CHOLLET-XEMARD | Tél : 01 45 17 95 39  Mail  [charlotte.chollet@aphp.fr](mailto:charlotte.chollet@aphp.fr)  Fax : 01 45 17 95 30 |
| 010 | Service de Médecine Intensive et Réanimation – Hôpital Cochin | Pr | Alain CARIOU | Tél : 01 58 41 25 01  Mail : [alain.cariou@aphp.fr](mailto:alain.cariou@aphp.fr)  Fax : 01 58 41 25 05 |
| 011 | Service de Médecine Intensive et Réanimation - Hôpital Pitié Salpêtrière | Pr | Alain COMBES | Tél : 01 42 16 38 18  Mail : [alain.combes@aphp.fr](mailto:alain.combes@aphp.fr)  Fax : 01 42 16 38 17 |
| 012 | Service de Médecine Intensive et Réanimation EOLE - Hôpital Pitié Salpêtrière | Pr | Alexandre DEMOULE | Tel : 01 42 16 78 58  Mail : [alexandre.demoule@aphp.fr](mailto:alexandre.demoule@aphp.fr)  Fax : 01 42 16 78 43 |
| 013 | Service de Médecine Intensive et Réanimation et toxicologique - Hôpital Lariboisière | Pr | Bruno MEGARBANE | Tel : 06 60 22 18 04  Mail : bruno.megarbane@aphp.fr  Fax : 01 49 95 65 78 |
| 014 | Service de Médecine Intensive et Réanimation - Hôpital Bichat | Pr | Jean-François TIMSIT | Tel : 01 40 25 77 02  Mail : [jean-francois.timsit@aphp.fr](mailto:jean-francois.timsit@aphp.fr)  Fax : 01 40 25 88 37 |
| 015 | Service de Médecine Intensive et Réanimation - Hôpital Ambroise Paré | Pr | Antoine VIEILLARD-BARON | Tel : 01 49 09 58 92  Mail : [antoine.vieillard-baron@aphp.fr](mailto:antoine.vieillard-baron@aphp.fr)  Fax : 01 49 09 58 92 |
| 016 | Service de Médecine Intensive et Réanimation - Hôpital Européen Georges Pompidou | Pr | Jean-Luc DIEHL | Tel : 01 56 09 32 01  Mail : [jean-luc.diehl@aphp.fr](mailto:jean-luc.diehl@aphp.fr)  Fax : 01 56 09 32 02 |
| 017 | Service de Médecine Intensive et Réanimation - Hôpital Bicêtre | Pr | Christian RICHARD | Tel : 01 45 21 35 45  Mail : [christian.richard@aphp.fr](mailto:christian.richard@aphp.fr)  Fax : 01 45 21 35 51 |
| 018 | Service de Médecine Intensive et Réanimation - Hôpital Henri Mondor | Pr | Armand MEKONTSO DESSAP | Tel : 01 49 81 23 94  Mail : [armand.dessap@aphp.fr](mailto:armand.dessap@aphp.fr)  Fax : 01 49 81 49 43 |

## Formulaire de notification des Evénements Indésirables Graves

## Formulaire de notification et de suivi d’une grossesse

## Echelle CPC score

Echelle CPC score, d’après Ajam et al. Scand J Trauma Resusc Emerg Med 2011 (doi : [10.1186/1757-7241-19-38](https://dx.doi.org/10.1186%2F1757-7241-19-38))
