# Supplementary material for: Evaluation of the effectiveness of potassium chloride in the management of out-of hospital cardiac arrest by refractory ventricular fibrillation: Study protocol of the POTACREH study
Source: PLoS One. 2023 Apr 12;18(4):e0284429. doi: 10.1371/journal.pone.0284429 (PMC10096226; doi:10.1371/journal.pone.0284429)
Supplement: S2 File — (DOC) [file pone.0284429.s003.doc]

| "EVALUATION OF THE EFFICACY OF POTASSIUM CHLORIDE  IN THE MANAGEMENT OF OUT-OF-HOSPITAL CARDIAC ARREST  BY REFRACTORY VENTRICULAR FIBRILLATION".  POTACREH |
| --- |

PROTOCOL FOR INTERVENTIONAL RESEARCH INVOLVING THE HUMAN BEING AND A MEDICINAL PRODUCT FOR HUMAN USE

Version N°1.1 of 18/12/2019

Project code: APHP180577 / EUDRACT N°: 2019-002544-24

**Coordinating investigator:** Pr Benoît VIVIEN

Service d’Anesthésie - Réanimation - SAMU

Hôpital Necker – Enfants malades

Tél : 01 44 49 23 67

Courriel : [benoit.vivien@aphp.fr](mailto:benoit.vivien@aphp.fr)

**Scientific Officer:** Dr Romain JOUFFROY

Service d’Anesthésie - Réanimation - SAMU

Hôpital Necker – Enfants malades

Tél : 01 44 49 24 75

Courriel : [romain.jouffroy@aphp.fr](mailto:romain.jouffroy@aphp.fr)

**Promoter:** Assistance Publique – Hôpitaux de Paris (AP-HP)

et par délégation : Direction de la Recherche Clinique et de l’Innovation (DRCI)

Hôpital Saint-Louis

1, avenue Claude Vellefaux

Référent projet DRCI-Siege : Sylvie PRIEUR

Tél : 01 44 84 17 92

Courriel : [sylvie.prieur@aphp.fr](mailto:sylvie.prieur@aphp.fr)

Référent vigilance DRCI-Siège : Céline LE GALLUDEC

Courriel : [celine.legalludec@aphp.fr](mailto:celine.legalludec@aphp.fr)

**Structure responsible for**

**monitoring of the research:** Unité de Recherche Clinique (URC)

Paris Descartes Necker Cochin

Hôpital Necker-Enfants malades

Responsable : Pr Jean-Marc TRELUYER

Référent projet DRCI-URC : Nelly BRIAND

Tél 01 44 38 18 62

Courriel : [nelly.briand@aphp.fr](mailto:nelly.briand@aphp.fr)

**Structure in charge of**

**the medication circuit:** Département Essais Clinique (DEC)

AGEPS-Paris

Pharmacien Référent projet: Dr Florence EMPANA-BARAT

Chef de projet : Elodie PERROT

Tél Secrétariat : 01 46 69 14 02

Courriel : [florence.barat@aphp.fr](mailto:florence.barat@aphp.fr) / [elodie.perrot@aphp.fr](mailto:elodie.perrot@aphp.fr)

Délégation à la Recherche Clinique et à l’Innovation (DRCI)

Hôpital Saint Louis 75010 PARIS

**Signing page of a research agreement**

**Research Code: APHP180577**

Title: Evaluation of the effectiveness of potassium chloride in the management of out-of-hospital cardiac arrest due to refractory ventricular fibrillation

Version N° 1.1 of 19/12/2019

The research will be conducted in accordance with the protocol, the good practices in force and the legislative and regulatory provisions in force.

| **Coordinating investigator**: |  |
| --- | --- |
| Pr Benoît Vivien  Service d’Anesthésie - Réanimation - SAMU Hôpital Necker – Enfants malades  Paris | Date : ……………/………/………..  Signature : |
| **Promoter** |  |
| Assistance Publique – Hôpitaux de Paris  Délégation à la Recherche Clinique et à l’Innovation (DRCI)  Hôpital Saint Louis  1 avenue Claude Vellefaux  75010 PARIS | Date : ……………/………/………..  Signature : |

**TABLE OF CONTENTS**

1 Synoptic summary 5

2 Scientific justification for the research 8

2.1 Hypotheses of the research 8

2.2 Description of knowledge of the disease concerned 8

2.3 Summary of the pre-clinical experiments and clinical trials concerned 10

2.4 Pharmacokinetics of KCl 12

2.5 Description of the population to be studied and justification for its choice 12

2.6 Name and description of the investigational medicinal product(s) 13

2.7 Description and justification of the dosage, route of administration, administration schedule and duration of treatment 13

2.8 Summary of the foreseeable and known benefits and risks to the persons undergoing the research 13

3 Objectives 15

3.1 Primary objective 15

3.2 Secondary objectives 15

4 Research design 15

4.1 Criteria for evaluating the research 15

4.2 Description of the research methodology 16

5 Conduct of the research 17

5.1 Inclusion 17

5.2 Intervention 18

5.3 Follow-up in the research 18

5.4 End-of-research visit 19

5.5 Summary diagram of the research timeline 19

5.6 Expected duration of participation of individuals, description of the timeline and duration of the research. 21

5.7 Distinction between care and research 21

6 Eligibility criteria 22

6.1 Inclusion criteria 22

6.2 Non-inclusion criteria 22

6.3 Recruitment procedures 22

6.4 Stopping rules 22

7 Treatment of research subjects 24

7.1 Description of the investigational drug(s) 24

7.2 Description of the ancillary treatment(s) (medicines needed to carry out the research) 24

7.3 Description of the traceability elements that accompany the investigational medicinal product(s) 24

7.4 Permitted and prohibited treatments (medicinal, ancillary, surgical), including rescue medication 24

7.5 Methods for monitoring compliance with treatment 24

8 Safety assessment - risks and constraints added by the research 24

8.1 Procedures in place for recording and reporting adverse events 24

8.2 Roles of the sponsor 25

9 Data management 30

9.1 Data collection procedures 30

9.2 Identification of data collected directly in the CRF which will be considered as source data 30

9.3 Right of access to source data and documents 30

9.4 Data processing and retention of documents and data 31

9.5 Ownership of data 31

10 Statistical Aspects 32

10.1 Assumptions for calculating the number of subjects needed and the result 32

10.2 Description of planned statistical methods including the timing of planned interim analyses 32

11 Quality Control and Assurance 33

11.1 General organisation 33

11.2 Quality control 33

11.3 Observation book 33

11.4 Management of non-conformities 34

11.5 Audit/inspection 34

11.6 Principal investigator's responsibilities 34

12 Ethical and legal aspects 35

12.1 Procedures for informing and obtaining the consent of the persons taking part in the research 35

12.2 Prohibition of participation in other research or period of exclusion from research, if applicable 35

12.3 Permission of the premises 35

12.4 Legal obligations 35

13 Funding and Insurance 36

13.1 Source of funding 36

13.2 Insurance 36

14 Rules on Publication 38

14.1 Mention of APHP affiliation for projects promoted by the APHP 38

14.2 Mention of the APHP sponsor (Drci) in the manuscript acknowledgments 38

14.3 Mention of the funder in the "Acknowledgments" of the Manuscript 38

15 Bibliography 39

16 List of Addenda 40

16.1 List of Investigators 40

16.2 CPC Score Scale 41

# 1 SYNOPTIC SUMMARY

| Titre | Evaluation of the effectiveness of potassium chloride in the management of out-of-hospital cardiac arrest due to refractory ventricular fibrillation |
| --- | --- |
| Acronym | POTACREH |
| Coordinating investigator | Pr Benoît Vivien |
| Scientific officer | Dr Romain Jouffroy |
| Promoter | Assistance Publique – Hôpitaux de Paris |
| Scientific justification | Out-of-hospital cardiac arrest (OHCA) has a poor prognosis, with an overall survival rate of around 5% at hospital discharge. Cardiac arrests (CA) in shockable rhythm (ventricular fibrillation (VF) and pulseless ventricular tachycardia) have a better prognosis.  In the case of shockable rhythm, treatment is based on defibrillation, then in case of failure of 3 external electric shocks (EES), on the administration of 300 mg of amiodarone in direct intravenous (IVD), followed by an additional 150 mg in case of inefficiency of 2 other EES. Lidocaine, which has long been used in this indication, is only recommended if amiodarone is unavailable or fails. Nevertheless, these 2 drugs, while they may be effective in terminating refractory VF, have marked cardiodepressor effects (bradycardia and/or negative inotropic effect) that persist after IVD administration. This explains, at least in part, why a recent study showed no significant difference in survival at hospital discharge between amiodarone, lidocaine and placebo in patients with refractory VF OHCA.  During surgery with extracorporeal circulation, cardioplegia solution is administered to interrupt cardiac activity and facilitate the surgical procedure. Physiopathologically, the mode of action of these solutions is based on a high concentration of potassium that lowers the resting membrane potential of the myocytes. By extension, it has been shown that the IVD administration of 20 mmol of potassium chloride (KCl) can cause a VF to cease, leading to a return to an organised hemodynamically effective heart rhythm within a few minutes. Kalemia levels measured were at the upper limit of normal (5.5 mmol/l) 10 min after this injection, and normal at 20 min. A recent clinical case of a resuscitation patient on bypass in refractory VF showed the effectiveness of IVD injection of 3 g of KCl, allowing a return to sinus rhythm in a few minutes.  The immediate advantage of KCl, compared to amiodarone (and also lidocaine), is the absence of a persistent cardiodepressor effect (bradycardia and/or hypotension) at a distance from the injection. The mode of action of the IVD injection of KCl to induce VF is indeed related to the peak of hyperkalaemia, and since kaliemia then normalises rapidly within a few minutes, there is no subsequent persistent deleterious effect following this injection of KCl. Furthermore, in the case of CA, as the patient is already under continuous external cardiac massage, no deleterious consequences related to this transient hyperkaliemia are expected.  The IVD injection of KCl in a patient in HCA with refractory VF at 3 EES, instead of amiodarone, should allow the interruption of this VF and then allow a rapid return to an organized cardiac rhythm, and thus the restoration of an efficient spontaneous cardiac activity. |
| Objective and primary endpoint | The primary objective is to evaluate the efficacy of an IVD injection of 20 mmol KCl on survival to hospital admission in patients undergoing OHCAF with refractory VF to 3 EES.  The primary endpoint is patient survival on hospital admission. |
| Objectives and secondary evaluation criteria | The secondary objectives are, in these patients, to evaluate the effectiveness of an IVD injection of 20 mmol KCl on  - the percentage of return to spontaneous cardiac activity in the pre-hospital setting,  - the time to return to spontaneous cardiac activity in the pre-hospital setting,  - the total dose of adrenaline administered in the pre-hospital setting,  - the total number of external electric shocks delivered in the pre-hospital setting,  - the number of persistent or recurrent rhythm disturbances requiring a pre-hospital ERC,  - haemodynamic parameters on admission to hospital,  - survival at hospital discharge with a good neurological prognosis (CPC scores 1 and 2),  - 3-month survival with good neurological prognosis (CPC scores 1 and 2).  The secondary endpoints were  - return to spontaneous cardiac activity in the pre-hospital setting,  - time to return to spontaneous cardiac activity in the pre-hospital setting,  - the total dose of adrenaline administered in the pre-hospital setting,  - the total number of external electric shocks delivered in the pre-hospital setting,  - the number of persistent or recurrent rhythm disturbances requiring a pre-hospital ERC,  - haemodynamic parameters on admission to hospital,  - survival at hospital discharge with a good neurological prognosis (CPC scores 1 and 2),  - survival at 3 months with a good neurological prognosis (CPC scores 1 and 2). |
| Experimental design | Prospective non-comparative phase II clinical trial. |
| Population concerned | Population concerned Adult patient, victim of an out-of-hospital cardiac arrest of presumed cardiac origin, with refractory ventricular fibrillation despite 3 external electric shocks. |
| Inclusion criteria | - Adult patient (age ≥ 18 years), - Victim of an OHCA of presumed cardiac origin with refractory VF despite 3 EESs,   - Covered by a health insurance plan. |
| Non-inclusion criteria | - Proven pregnancy,  - Incapacitated adult (patient under guardianship or curatorship); |
| Expected benefits for participants | To improve the probability of survival for each refractory OHCA patient in the study. |
| Risks added by the research | These are patients with refractory CA, already on external cardiac massage and artificial ventilation. In this context, no a priori deleterious consequences related to this transient hyperkaliemia are expected. |
| Practical implementation | Research risk level: D |
| Number of subjects selected | The experimental plan is a phase II of Simon in 2 stages. |
| Number of centres | * 34 patients will be included in the first stage. |
| Duration of the research | * If 12 or fewer successes are observed, the trial will not be continued and it will be concluded that KCl is not effective.  * If not, if at least 13 successes are observed, the trial will continue with the inclusion of 47 additional patients. |
| Number of planned inclusions per centre per month | The treatment will be considered effective under these assumptions if at least 34 successes are observed in total. |
| Statistical analysis | 81 |
| Source of funding | National multi-centre research |

**2. SCIENTIFIC JUSTIFICATION OF THE RESEARCH**

**2.1 Research hypotheses**

Direct intravenous administration of potassium chloride (KCl) should allow immediate interruption of refractory ventricular fibrillation (VF) in patients suffering from out-of-hospital cardiac arrest (OHCA), allowing a return to an organised spontaneous cardiac rhythm within a few minutes, associated with a resumption of effective spontaneous circulatory activity (RACS).

This modification of the management strategy for patients suffering from refractory VF OHCA should improve the prognosis of these patients in terms of survival at hospital discharge.

**2.2 Description of knowledge of the disease concerned**

Unexpected cardiac arrest (CA), or sudden adult death, is defined as the sudden interruption of spontaneous circulation. This condition is a major public health problem in industrialised countries, affecting more than 420,000 patients in the USA each year. In France, it is estimated that between 30,000 and 50,000 cardiac arrests (CAs) occur each year, 85% of which occur outside hospitals [1].

The classification of cardiac arrest, both in and out of hospital, is based on the analysis of the first rhythm identified by the electrocardioscopic tracing. Thus, a distinction is made between shockable rhythms, i.e. those justifying the administration of an external electric shock (EES): ventricular fibrillation (VF) and pulseless ventricular tachycardia (VT), and non-shockable rhythms: asystole and pulseless rhythms [2]. The prognosis of shockable rhythm CA is better than that of non-shockable rhythm CA, due to the potential efficacy of an EES allowing a return to a hemodynamically effective spontaneous heart rhythm.

OHCA has a poor prognosis, with an extremely low overall survival rate at discharge, classically considered to be around 5% [2]. A meta-analysis published in 2010 showed that the prognosis of OHCA had changed little in the 30 years prior to this publication, with an overall survival rate of 7.6% at hospital discharge [3]. However, more recent data suggest that the overall prognosis of OHCA has improved significantly, from 8.2% survival at hospital discharge in 2006 to 10.4% in 2010. On the other hand, if we look more specifically at patients suffering from OHCA due to shockable rhythms (VF and pulseless VT), rhythms with a better prognosis because they are accessible to early defibrillation, the prognosis increases from 23.5% survival at hospital discharge in 2006 to 30.3% in 2010 [4].

The management of a patient in AC follows international recommendations established by a consensus of experts and grouped within the ILCOR (International Liaison Committee on Resuscitation). The latest version of these ILCOR recommendations was published in 2015 by the American Heart Association (AHA) [5] and the European Resuscitation Council (ERC) [6]. Among other things, the cardiopulmonary resuscitation (CPR) algorithm differentiates between the management of shockable and non-shockable rhythms, by performing an ERC in the former case (fig. 1).

**
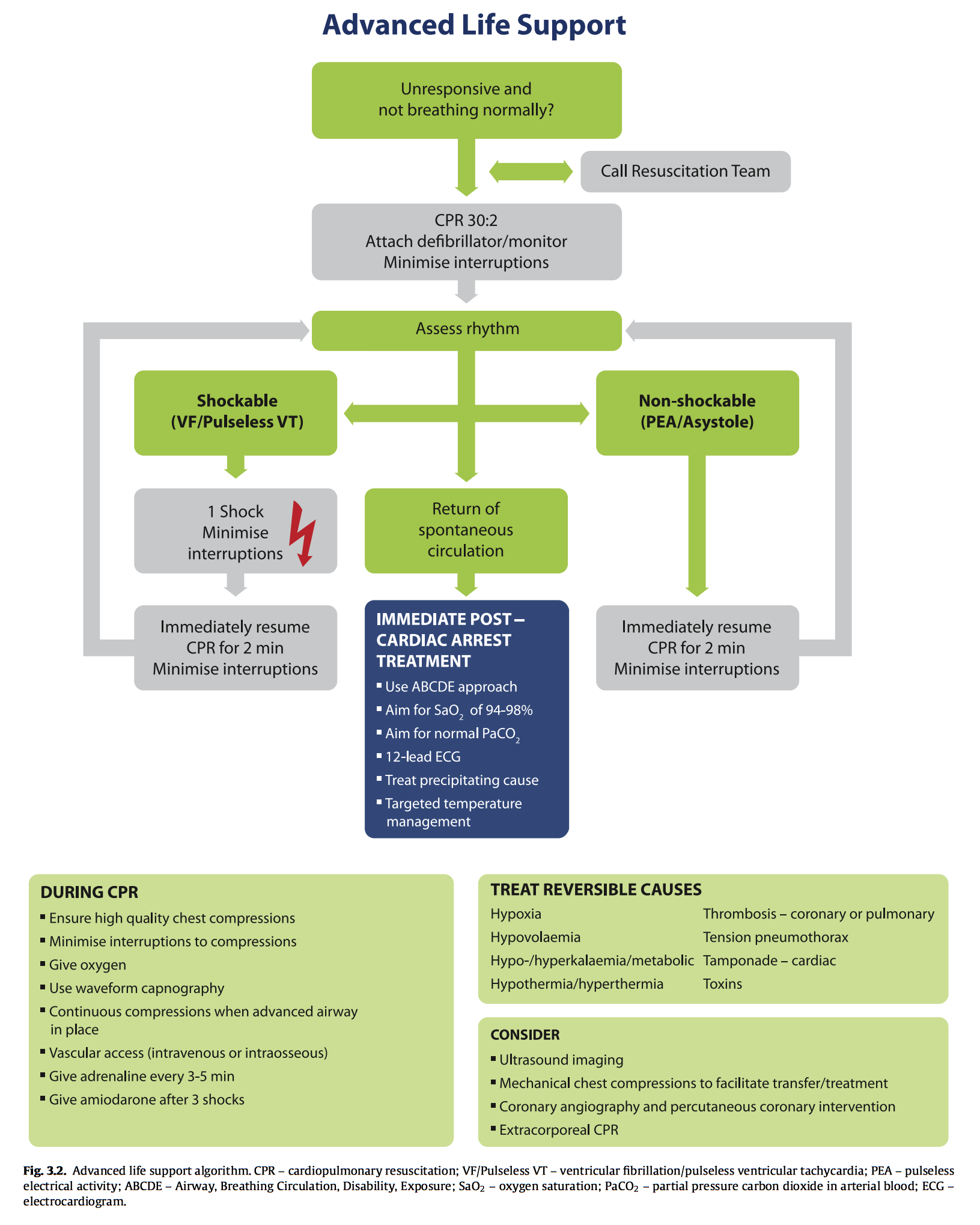
**

**Figure 1:** Algorithm for medical CPR, European Resuscitation Council (ERC) version [6].

Although in the case of a shockable rhythm, defibrillation most often allows the return of a haemodynamically effective heart rhythm, this therapy is sometimes ineffective. This is why international recommendations recommend the intravenous injection of an anti-arrhythmic drug beyond the 3rd ERC. Two drugs are classically recommended in this indication, described as shockable rhythm CA "refractory" to EESs: amiodarone (300 mg IVD) in the first line, and lidocaine (1 mg/kg) in the second line [2].

However, while these two drugs may, according to studies published in the literature, increase the rate of return to effective spontaneous circulatory activity, and/or the rate of patients admitted alive to hospital, it should be noted that the results are much more mixed regarding survival at 3 months and/or at hospital discharge, which is the "strong" criterion generally used to assess survival after HCA. Thus, one of the most recent major studies on this topic was published in the New England Journal of Medicine by Kudenchuk et al. in 2016 [7]. In this work the authors compared the administration of amiodarone to lidocaine and placebo, in 3026 patients with OHCA by VF or TV without refractory pulses at 3 EES. While amiodarone and lidocaine did increase the rate of patients admitted alive to hospital (respectively 45.7% vs 39.7%, p=0.01; and 47.0% vs 39.7%, p<0.001), with no statistical difference between these 2 molecules (45.7% vs 47.0%, p=0.55), it should be noted that there was no significant difference in terms of survival at hospital discharge between amiodarone, lidocaine and placebo (amiodarone vs placebo: 24.4% vs 21.0%, p=0.08; lidocaine vs placebo: 23.7% vs 21.0% p=0.16; amiodarone vs lidocaine: 24.4% vs 23.7%, p=0.70).

These non-significant results in terms of survival can probably be explained by the pharmacokinetic and pharmacodynamic properties of the two antiarrhythmic molecules, amiodarone and lidocaine. Amiodarone, a class III antiarrhythmic according to the Vaughan-Williams classification, has a bradycardia effect and a negative dromotropic effect (slowing of conduction), which are more pronounced when administered in high doses and by the intravenous route, which is the case in refractory CA. One of the side effects of lidocaine, a Vaughan-Williams class Ib antiarrhythmic, is a myocardial depressant effect, characterised by hypotension, myocardial depression and bradycardia. It is therefore perfectly possible that the beneficial effects of these two molecules in terms of immediate RACS are counterbalanced by their "persistent" negative cardiovascular side effects when administered intravenously in high doses, and therefore ultimately do not improve survival at hospital discharge.

Furthermore, it should be noted that the survival rates at discharge in the amiodarone (24.4%), lidocaine (23.7%) and placebo (21%) groups in the Kudenchuk et al. study [7] may appear at first glance to be much lower than the 30.3% survival at discharge in the Daya et al. study published in 2010 [4]. However, it should be noted that Daya et al. analysed all patients with a shockable rhythm, whereas Kudenchuk et al. only analysed patients with a shockable rhythm that was already refractory at 3 EESs, which explains the poorer prognosis in this second study.

It therefore appears from the analysis of the literature that no molecule is more effective than a placebo in terms of survival at hospital discharge in patients presenting with OHCA by VF or pulseless VT refractory to 3 EES. Further pharmacological research is therefore needed to try to improve the prognosis of these patients.

**2.3. Summary of relevant pre-clinical experiments and clinical trials**

During surgery performed under extracorporeal circulation (ECG), particularly in valvular and/or coronary heart surgery, a cardioplegia solution is administered to interrupt the mechanical activity of the heart and thus facilitate the surgical procedure. Various cardioplegia solutions are available, varying in their chemical composition. However, pathophysiologically, the essential component of these solutions is always potassium chloride at a high concentration, usually in the range of 20-30 mmol/L [8]. The direct intravenous administration of this solution is then responsible for a transient hyperkalaemia, which has the immediate consequence of lowering the membrane resting potential of the myocytes, leading to the cessation of their electrical and mechanical activity, and consequently to the immobility of the cardiac muscle.

As these cardioplegia solutions are effective in stopping myocardial electrical activity during the implementation of a bypass surgery, some authors have by extension evaluated their effectiveness in interrupting ventricular fibrillation, essentially in the context of aortic unclamping after bypass surgery, with the exception of the first clinical case presented below.

Indeed, the first scientific publication describing the administration of potassium chloride in humans to convert ventricular fibrillation is a clinical case published by Weinstock and Clark in 1961 [9]. The authors report the case of a 3 year old child presenting with refractory VF after an impossible intubation in the operating theatre, which was eventually converted to sinus rhythm by the intracardiac injection of 4 mmol of potassium chloride. The child subsequently survived with completely normal psychomotor development according to the authors.

In 1984, Robicsek et al. published a series of 12 patients in whom an injection of potassium chloride resulted in the cessation of VF following cardiac surgery with bypass graft and cardioplegia [10].

Eleven years later, Øvrum et al. described a series of 200 cardiac surgery patients undergoing bypass surgery and cardioplegia [11]. An injection of 20 mmol potassium chloride was performed in 100 patients, resulting in VF failure in 82% of cases. The remaining 18 patients who received 20 mmol of potassium chloride but did not achieve VF regulation required fewer ERCs (p<0.005) to achieve VF closure compared to the 100 patients in the control group.

A very similar study to the previous one was published by Almdahl et al. in 2013 on a group of 8465 patients operated on under ECC with cardioplegia [12]. In this study, 1721 patients with VF after aortic decompression received an injection of potassium chloride, which resulted in the cessation of the rhythm disorder in 1366 (79.1%). Of all the patients in whom this injection was effective, the dose of potassium required was 20 mmol in 88% of cases, and 30 mmol in 12% of cases. No deleterious effect linked to this injection of potassium chloride was reported in this study. Only 355 patients (20.9%), in whom potassium chloride injection was ineffective, required one or more electric shocks to stop the VF. The main advantage of this "pharmacological defibrillation" put forward by the authors is the absence of need to interrupt surgery to place the defibrillation paddles, and consequently the absence of risk of iatrogenic injury to the coronary bypasses linked to the positioning of these paddles and the movement induced during the ERC.

Apart from these cases of VF occurring after cardiac surgery under CEC and cardioplegia, several publications report the effectiveness of an injection of potassium chloride to treat pulseless VF or VT refractory to defibrillation by electric shocks.

For example, in a porcine animal model (35±2 kg), Liakopoulos et al. demonstrated the efficacy of potassium chloride injection (15±2 mEq) in VF lasting for more than 10 minutes [13].

A series of 3 patients with rhythmic storm with refractory VT and VF after aortic decompression after bypass surgery was published in 2011 by Watanabe et al [14]. These 3 patients had refractory VF or VT despite 5-10 internal electric shocks of 50 J. An injection of 20 mEq of potassium chloride allowed each of them to relinquish the refractory ventricular rhythm disorder, with a return to an organised and haemodynamically effective heart rhythm.

Finally, we have recently published a case of a resuscitation patient undergoing bypass surgery who presented with refractory VF after a OHCA of ischaemic origin [15]. IVD injection of 3 g (40 mmol) of potassium chloride resulted in a return to sinus rhythm within minutes, with no subsequent recurrence of rhythm disturbances, and the patient was discharged alive with a good neurological prognosis.

These various studies and clinical cases reporting the effectiveness of an injection of potassium chloride are supported by experimental data. For example, it has been shown in an isolated endocardial fibre model that a concentration of 12 mmol/l of potassium chloride was able to induce VF with a consequent return to an organised electrical rhythm [16]. A recent electrophysiological study in an isolated heart model showed that hyperkaliemia mainly affects the dynamic character and temporal-spatial organisation of VF [17].

The question that obviously arises after an injection of potassium chloride is the pharmacokinetics of this hyperkalaemia. Although studies are limited in this area, data are available in the literature. For example, in their series of 100 patients injected with 20 mmol of potassium chloride, Øvrum et al. report blood kaliemia values at the upper limit of normal (5.5±1.0 mmol/l) 10 minutes after the injection, and back to normal values (4.3±0.4 mmol/l) after 20 minutes [11]. This therefore attests to the extremely transient nature of this hyperkaliemia during a direct intravenous injection of 20 mmol of potassium chloride.

Finally, concerning the possible morbimortality linked to an injection of potassium chloride, Almdahl et al. published reassuring data in a second article [18] using the same methodology as their first article [12]. In a series of 12113 cardiac surgery patients undergoing bypass surgery and cardioplegia, survival at D30 was not different between the 9723 patients who did not develop post bypass VF and the 1877 patients who developed VF successfully converted by 20 mmol potassium chloride injection (1.2% vs. 1.32%, p=0.269). In contrast, there was a trend towards increased long-term mortality in the 400 patients with VF resistant to "pharmacological defibrillation" and therefore requiring an internal electric shock (hazard ratio=1.19 CI95% [0.99-1.4], p=0.07). These results are therefore reassuring with regard to possible deleterious consequences that could have been linked to the injection of potassium chloride. They also suggest (but with all the reservations of non-statistically significant results) that this pharmacological defibrillation could improve survival in comparison with conventional defibrillation by electric shocks (in this case internal electric shocks in this work since it concerns cardiac surgery patients).

All of this work therefore allows us to envisage that a direct intravenous injection of 20 mmol of potassium chloride, in a patient in OHCA presenting a VF or VT without a refractory pulse at 3 EES, will make it possible, thanks to a transient hyperkalaemia, to make this rhythm disorder cease and consequently authorize the return to an organized and effective cardiac rhythm on the hemodynamic level, and this without any immediate or long-term deleterious consequence.

**2.4. Pharmacokinetics of KCl**

The body contains 3,500 mmol of potassium, 98% of which is distributed in the intracellular sector and represents the majority of exchangeable potassium. Red blood cells store 2% of the intracellular potassium, or 70 mmol. The extracellular sector contains 80 mmol of K+ or 2% of the total potassium. The maintenance of the gradient between the extracellular (Ke) and intracellular (Ki) sectors is actively carried out by the Na+K+-ATPase pump and by intracellular electronegativity.

Short-term variations in kalaemia, such as during the administration of a loading dose of potassium by direct intravenous route, are regulated by these transmembrane exchanges, essentially linked to the Na+K+-ATPase pump. In contrast, urinary excretion of potassium is a much slower phenomenon, dependent on its tubular secretion in the distal nephron which adjusts renal losses to daily intakes [19]. It should therefore be considered that the kidney regulates the potassium balance not in the short term, but in the medium term [20].


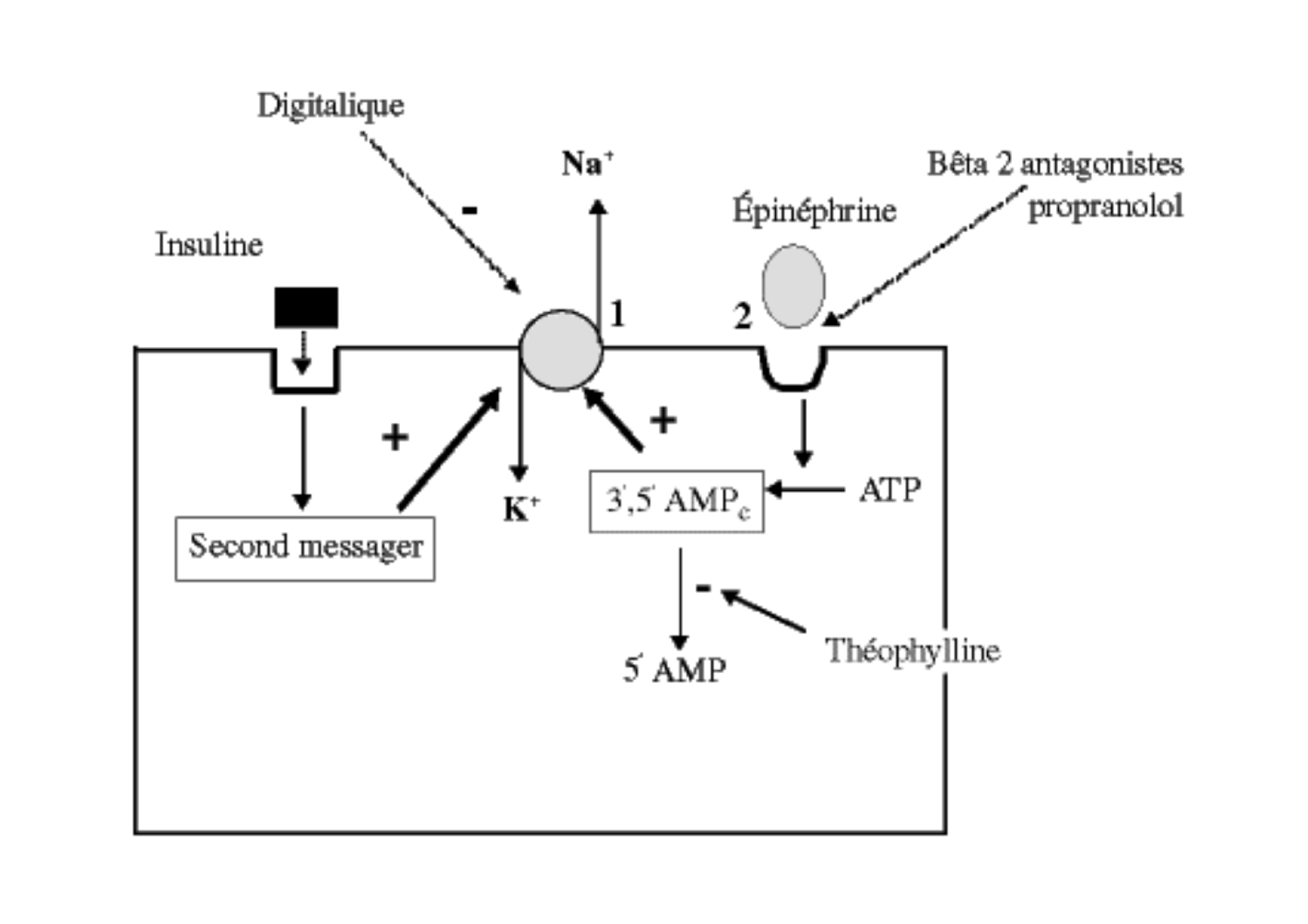
**Figure 3:** Regulation of potassium flows between the intra-cellular and extra-cellular compartments (after [21]).

## This mode of short-term regulation of kaliemia linked to the Na+K+-ATPase pump makes it possible to explain that an injection of 20 mmol of potassium by direct intravenous route results in a transient hyperkalaemia, with the return to a kaliemia value at the upper limit of the normal 10 min. after the injection, and returned to the normal values after 20 min. As a consequence of this rapid decrease in kaliemia during a direct intravenous injection, it can be considered that the renal elimination of potassium therefore plays a minor role in this decrease, in comparison with the immediate activation of the Na+K+-ATPase pump which will favour the transfer of potassium from the extra-cellular compartment to the intra-cellular compartment. Consequently, in a patient with renal insufficiency, the complete absence of urinary elimination should have little impact on the pharmacokinetics of potassium in comparison with a subject with preserved renal function.

## Finally, we should also mention the role of catecholamines, which favour the intracellular transfer of potassium by stimulation of beta 2 adrenergic receptors via cAMP [22]. In the case of a patient in cardiac arrest, the administration of high doses (1 mg IVD) of adrenaline, stimulating both alpha and beta-adrenergic receptors, will therefore further activate the transmembrane Na+K+-ATPase, which will further promote the transfer of potassium from the extracellular compartment to the intracellular compartment.

## 2.5. Description of the population to be studied and justification for its choice

## The population studied will concern adult patients (age greater than or equal to 18 years) victims of out-of-hospital cardiac arrest of presumed cardiac origin and presenting a shockable rhythm, i.e. ventricular fibrillation, refractory in spite of the realization of 3 external electric shocks. The choice of this population was made because of the absence of anti-arrhythmic treatment (amiodarone and lidocaine) having demonstrated superior efficacy against placebo on improving survival at hospital discharge. On the other hand, it has been observed that the administration of potassium chloride IVD in cardiac surgery and in intensive care has been effective in interrupting VF or VT without refractory pulse, without deleterious effect and allowing a good neurological prognosis at hospital discharge. It is therefore legitimate to evaluate the efficacy of an injection of potassium chloride IVD to interrupt refractory ventricular fibrillation after 3 external electric shocks.

## 2.6. Name and description of the investigational medicinal product(s)

## - Name of the medicinal product: Potassium chloride (KCl) 10% ampoule, solution for infusion.

## - Dosage used: 20 mmol of potassium chloride, i.e., 14.9 ml of 10% KCl, which is pragmatically rounded up to 15 ml of 10% KCl.

## - Modalities and route of administration: single direct intravenous administration (IVD).

**2. 7. Description and rationale of dosage, route of administration, schedule and duration of treatment**

The dosage of 20 mmol KCl by direct intravenous injection for this study is the same as that used in most of the previously reported studies in adults. However, it should be noted that in these studies, KCl was generally administered intra-aortically and/or via the bypass graft, which is obviously not possible in our study of patients in out-of-hospital cardiac arrest. The route of administration will therefore be direct peripheral intravenous. This is a single "flash" administration, with the aim of obtaining a peak in hyperkalaemia, so the duration of treatment stricto sensu (= duration of administration) will be of the order of a few seconds.

In parallel to this injection, specialised cardiopulmonary resuscitation will be performed in a conventional manner in accordance with international recommendations and good clinical practice, particularly in terms of external cardiac massage and ventilation. The only difference from international guidelines is that once the patient has received 3 EESs, instead of the recommended 300 mg of amiodarone IVD, the patient will receive a single dose of 20 mmol potassium chloride IVD (i.e. 14.9 ml rounded to 15 ml of 10% KCl). Amiodarone 300 mg, if required due to refractory or recurrent VF, will simply be "delayed" by 2 EESs for research purposes.

Specialist cardiopulmonary resuscitation will then be continued in the conventional manner, again in accordance with international recommendations. In particular, electric shocks will be repeated if the patient presents persistent or recurrent ventricular fibrillation after this injection of 20 mmol potassium chloride IVD.

**2.8. Summary of the foreseeable and known benefits and risks for the persons undergoing the research**

This injection of 20 mmol IVD potassium chloride may allow for the immediate interruption of refractory VF, with a consequent rapid return to a hemodynamically effective spontaneous heart rhythm.

The effectiveness of potassium chloride in interrupting refractory VF is indeed related to the peak of hyperkalaemia, and it has been shown that the kaliemia measured 10 minutes after this injection is borderline normal (5.5 mmol/l) and normal after 20 minutes. There is therefore no risk of a persistent cardiovascular depressant effect once the hyperkaliemia peak has disappeared. Furthermore, the second study by Almdahl et al, certainly in a post-operative context of cardiac surgery under CEC, clearly showed the absence of specific morbimortality in the 1877 patients who presented a VF successfully converted by an injection of 20 mmol of potassium chloride [18].

In contrast, amiodarone and lidocaine, the reference drugs according to international guidelines for interrupting refractory VF, are characterised by cardiovascular depressant effects that persist well after direct intravenous administration.

The injection of potassium chloride IVD, instead of amiodarone or lidocaine, could therefore allow, like these last two molecules, the immediate interruption of refractory VF, but without the risk of a persistent depressant effect on the cardiovascular level.

The possible risk of this IVD injection of potassium chloride can be considered as nil, since the patient is already in refractory cardiac arrest, under specialised cardiopulmonary resuscitation, thus with external cardiac massage to compensate for the absence of spontaneous cardiac activity, and manual or mechanical artificial ventilation to compensate for the absence of spontaneous ventilation.

The potential efficacy of potassium chloride injection in the treatment of refractory VF HCA would therefore be likely to improve the prognosis of patients with refractory VF during HCA. This should logically lead to a change in the international recommendations for this condition.

# 3. OBJECTIVES

# 3.1. Main objective

# The primary objective is to evaluate in patients in out-of-hospital cardiac arrest due to ventricular fibrillation refractory to 3 external electric shocks the efficacy of an intravenous injection of 20 mmol potassium chloride on survival to hospital admission, i.e. on the percentage of patients arriving at the hospital with effective spontaneous cardiac activity

# 3.2 Secondary objectives

# The secondary objectives are, in these patients, to evaluate the efficacy of an IVD injection of 20 mmol potassium chloride on

# - the return to effective spontaneous cardiac activity in the pre-hospital setting,

# - time to return to pre-hospital spontaneous cardiac activity,

# - the total dose of adrenaline administered in the pre-hospital setting,

# - the total number of external electric shocks delivered in the pre-hospital setting,

# - the number of persistent and/or recurrent rhythm disturbances requiring an external electric shock in the pre-hospital setting,

# - haemodynamic parameters on admission to hospital,

# - survival at hospital discharge with a good neurological prognosis (cerebral performance category scores 1 and 2),

# - survival at 3 months with a good neurological prognosis (cerebral performance category scores 1 and 2).

# 4. RESEARCH DESIGN

# 4.1. Research evaluation criteria

The primary endpoint is patient survival on arrival at the hospital (with effective spontaneous cardiac activity).

The assessment will be qualitative: presence or absence of effective spontaneous cardiac activity measured at the time of the patient's arrival in the hospital ward (resuscitation or cardiac catheterisation room).

Secondary evaluation criteria

### The secondary endpoints are:

### - The return to spontaneous cardiac activity in the pre-hospital setting. The assessment will be qualitative: presence or absence of effective spontaneous cardiac activity prior to the patient's arrival in hospital.

### - Time to return to spontaneous cardiac activity in the pre-hospital setting. The assessment will be quantitative: time in minutes from the time of cardiac arrest to the return to effective spontaneous cardiac activity.

### - The total dose of adrenaline administered in the pre-hospital setting. The assessment will be quantitative: the total number of milligrams of adrenaline administered prior to the patient's arrival in the hospital department (resuscitation or cardiac catheterisation room) will be recorded.

### - The total number of external electric shocks delivered pre-hospital. The evaluation will be quantitative: the total number of shocks delivered before the patient's arrival in the hospital department (resuscitation or cardiac catheterisation room) will be recorded.

### - The number of persistent and/or recurrent rhythm disorders requiring an external electric shock in the pre-hospital setting. The evaluation will be quantitative: the total number of rhythm disturbances requiring an external electric shock before the patient's arrival in the hospital service (resuscitation or cardiac catheterisation room) will be recorded.

### - Haemodynamic parameters on admission to hospital. The assessment will be quantitative: heart rate and blood pressure will be measured at the time of the patient's arrival in the hospital ward (resuscitation or cardiac catheterisation room).

### - Survival at hospital discharge with a good neurological prognosis (Cerebral Performance Category scores 1 and 2). At discharge, the evaluation will be qualitative for survival (present or absent) and quantitative for the CPC score rated on a scale of 1 to 5.

### - Survival at 3 months with a good neurological prognosis (Cerebral Performance Category scores 1 and 2). At 3 months, the evaluation will be qualitative for survival (present or absent) and quantitative for the CPC score rated on a scale from 1 to 5.

### 4.2. Description of the research methodology

Experimental design

This is a prospective, non-comparative phase II clinical trial.

The objective of this phase II trial is to have a first evaluation of the efficacy of the treatment. If the efficacy proves to be satisfactory (survival at admission close to 50%), a national randomised trial will be carried out to compare the strategy including potassium chloride in the first line with the classic strategy.

In the absence of preliminary efficacy data in out-of-hospital cardiac arrest due to refractory VF, it would not have been relevant to propose a randomised placebo-controlled trial from the outset. Indeed, in this context, to show a clinically significant improvement in survival, a large number of subjects would have to be included (e.g., 1500 subjects per group to show an improvement in survival of 35% to 40%).

Number of participating centres

The centres participating in this regional multicentre research are:

- On the one hand, pre-hospital emergency services which will ensure the pre-hospital recruitment of the patients participating in this study,

- On the other hand, intensive care units in which the patients will be hospitalised after their pre-hospital care.

### Identification of subjects

### For the purposes of this research, subjects will be identified in the following way:

### centre number (3 numeric positions) - person's order of selection in the centre (4 numeric positions) - initial surname - initial first name.

### This reference is unique and will be kept for the duration of the research.

### **5. CONDUCT OF THE RESEARCH**

**Before any examination or act related to the research, the investigator obtains the free, informed and written consent of the person who is to be the subject of the research or of his or her legal representative, if any.**

**Persons likely to be involved in research mentioned in 1° of Article L. 1121-1 of the Public Health Code benefit from a prior medical examination appropriate to the research.**

**5.1. Inclusion**

Selection takes place during the pre-hospital management of a patient with HCA by a team from one of the participating centres. If the patient presents with a shockable VF rhythm, then he/she will be considered eligible. Screening will therefore take place a few minutes prior to inclusion if applicable.

After checking the inclusion and non-inclusion criteria, the investigator will include the patient if he/she meets the criteria defined by the study. In summary, this will be a patient with a OHCA with a VF authenticated on monitoring and for whom 3 EESs have not resulted in a return to a hemodynamically effective spontaneous heart rhythm.

Inclusion will therefore be based primarily on anamnestic data regarding the OHCA, clinical data and monitoring of the patient's heart rhythm. No additional biological or imaging tests are required for inclusion of the patient in the study.

As this is an immediate life-threatening emergency, a waiver of the requirement to seek consent prior to patient inclusion is required (Article L1122-1-3 of the Public Health Code).

**Figure 3:** Algorithm for the management of a patient with HEA by VF according to international recommendations (Fig. 3A) and in the research setting (Fig. 3B). Adrenaline injections are not shown in the figure for simplicity.

According to international recommendations [5,6], amiodarone 300 mg IVD should be administered for refractory (or recurrent) VF after 3 EES. If VF persists, a second administration of amiodarone IVD should be given at half dose (150 mg) after the 5th EES. If VF still persists, then continuous IV administration with an electric syringe at a dose of 900 mg/d can be started, or direct intravenous administration of lidocaine (1 mg/kg).

**5.2. Intervention**

For research purposes, direct intravenous injection of 20 mmol potassium chloride will be given after the 3rd ERC, instead of the 1st amiodarone administration. If necessary, the 1st injection of amiodarone 300 mg IVD will be given after the 5th EES, the 2nd injection 150 mg IVD after the 7th EES, with a relay with continuous amiodarone or the use of lidocaine afterwards.

**5.3. Follow-up in research**

No research-specific follow-up visits are planned.

Pre-hospital follow-up will be carried out by the pre-hospital investigating physician who included the patient.

Two evaluation times are planned:

- At the site of the OHCA at the end of the pre-hospital management of the patient, before the patient is taken to hospital or declared dead, the parameters collected will be:

- Return to spontaneous cardiac activity;

- Total dose of adrenaline administered;

- The total number of external electric shocks delivered;

- The number of persistent and/or recurrent rhythm disturbances requiring an external electric shock.

- At the time of the patient's hospital admission, if applicable, the parameters collected will be:

- Patient survival on arrival at the hospital (presence or absence of effective spontaneous cardiac activity), which is the primary endpoint of the study;

- Haemodynamic parameters at hospital admission.

At the end of the operation, the patient, if alive, and if necessary, after passing through the cardiac catheterisation room if this is indicated, will be taken to a resuscitation service where the initial management will be continued according to the usual practices of the service. The choice of treatments undertaken in the intensive care unit will not be affected by the pre-hospital management.

Follow-up in hospital will then be carried out by the hospital doctors receiving the patient after the pre-hospital care. Two times of evaluation of survival and neurological prognosis (Cerebral Performance Category score) are planned:

- at the time of the patient's discharge from hospital, if applicable.

- at 3 months (D90) +/- 1 week after patient inclusion.

These two assessments can be carried out on the basis of the hospitalization report(s) at the time of the patient's discharge from hospital and during telephone interviews with the patient himself/herself or a family member and/or a physician in charge of the patient at that time (call carried out by a centralized clinical study technician).

During hospitalization, the patient, or if necessary, the family members or the trusted person, will be informed as soon as possible of their inclusion in the research. His/her consent, if the patient is in a state to consent, or the consent of his/her relatives, will be requested for the continuation of the research (article L1122-1-3 of the Public Health Code).

The data collected during the follow-up will be anamnestic, clinical and electrocardioscopic monitoring data. No additional complementary examination, in particular electrocardioscopic, imaging or biological, will be carried out during this follow-up specifically in the context of this research. The evaluation of the CPC score will be considered as clinical data.

Postal, electronic and telephone contact details of the patient and a relative will be collected to allow follow-up of surviving patients at 3 months. The commune of birth will also be collected in order to verify the vital status of the patients and in case of death, its cause (RNIPP and CépiDC registers) if they are lost to follow-up at 3 months and it has not been possible to retrieve information from relatives.

**5.5. End-of-research visit**

No specific end-of-research visit is planned.

**5.6. Summary diagram of the research chronology**

The sequence of research assessment times is shown in the timeline in Figure 4.

- The assessment of patient eligibility takes place between the start of the patient's care by the pre-hospital medical team and the 3^rd^ EES.

- Inclusion takes place immediately after the 3^rd^ EES, if the patient still has VF after this ERC

- Depending on the patient's progress during in-hospital management, discharge from hospital may occur after the end of the 3rd month following the OHCA. But the assessment will always be done at the 3rd month if the patient is alive, whether discharged or still in hospital.

**Figure 4**: Research timeline.

| **Visit** | **Inclusion in**  **Pre-hospital** | **Intensive care unit admission** | **Discharge** | **Follow-up**  **(3 months)** |
| --- | --- | --- | --- | --- |
| **Inclusion - Non-inclusion criteria** | **X** |  |  |  |
| **Inclusion** | **X** |  |  |  |
| **Information** |  | **X** |  |  |
| **Collection of consent to continue** |  | **X** |  |  |
| **Clinical examination** | **X** | **X** | **X** |  |
| **Study treatment** | **X** |  |  |  |
| **Mortality assessment** | **X** | **X** | **X** | **X** |
| **CPC score assessment** |  |  | **X** | **X** |

**TABLE:** Research timeline summary

## 5.7. Expected duration of participation of individuals, description of the timeline and duration of the research.

## The duration of treatment for each subject is a few seconds (time to perform the IVD injection of potassium chloride).

## The expected duration of patient participation is 3 months.

## The expected duration of the inclusion period is 18 months.

## The total duration of the research is 21 months.

## 5.8. Distinction between care and research

## Figure 3 shows the patient management algorithm according to international recommendations (Fig. 3A), and in the case of this research (Fig. 3B).

## In practice, the key difference between these two algorithms is the administration of 20 mmol potassium chloride IVD after the 3rd ERC, instead of the first administration of amiodarone 300 mg IVD. Therefore, in the research setting, if necessary, in the event of failure of potassium chloride injection to interrupt refractory VF, the administrations of amiodarone 300 mg IVD, then 150 mg IVD, followed by continuous amiodarone administration or the use of lidocaine, will simply be "delayed" by 2 EESs compared to the management that would have been carried out in the strict sense according to international recommendations.

## TABLE: Distinction between procedures related to "care" and procedures added by "research

| **Acts, procedures and treatments carried out in the context of research** | **Acts, procedures and treatments related to care** | **Acts, procedures and treatments added by the research** |
| --- | --- | --- |
| **Treatments** | first administration of amiodarone 300 mg IVD | IVD administration of 20 mmol potassium chloride after the 3^rd^ EES |
| **Follow up** |  | CPC score assessment at discharge and at 3 months |

**6. Eligibility criteria**

**6.1. Inclusion criteria**

- patient over 18 years of age (age greater than or equal to 18 years).

- patient with out-of-hospital cardiac arrest of presumed cardiac origin and presenting refractory ventricular fibrillation despite 3 external electric shocks.

- patient with a health insurance plan.

**6.2. Non-inclusion criteria**

- proven pregnancy.

- incapacitated adult (patient under guardianship or curatorship).

- patient who still does not have a functional venous line after 3 external electric shocks.

**6.3. Recruitment procedures**

Adult patients suffering from out-of-hospital cardiac arrest of presumed cardiac origin with refractory ventricular fibrillation despite 3 external electric shocks will be consecutively included by a pre-hospital medical team of one of the 4 participating centres (SMUR, or resuscitation ambulance for the Paris fire brigade).

Given the experimental design chosen (Simon's phase ii in 2 stages): 34 patients will be included in the 1st stage, then, if the treatment is considered effective, the trial will continue with the inclusion of 47 patients with a total of 81 patients included.

| Total number of subjects selected | 81 |
| --- | --- |
| Number of recruiting centres | 4 |
| Inclusion period (months) | 18 |
| Number of subjects / centre | 21 |
| **Number of subjects / centre / month** | **1 à 2** |

**6.4. Discontinuation rules**

Criteria and modalities for premature discontinuation of the research treatment

As the treatment is administered in an IVD injection within seconds, no criteria for stopping treatment can be defined.

However, extravasation or injection failure implies exit from the experimental design for the participant.

Criteria and modalities for premature termination of a subject's participation in the research

Any subject may discontinue participation in the research at any time and for any reason.

The investigator may permanently discontinue a subject's participation in the research for any reason that affects the safety of the subject or is in the best interests of the subject.

If the subject is lost to follow-up, the investigator should make every effort to re-establish contact with the subject or the subject's relatives (and document this in the source file) in order to ascertain, at a minimum, whether the subject is alive or dead

The case report form should list the various reasons for discontinuing participation in the research:

- Adverse event
- Other medical problem
- Personal reason of the subject
- Explicit withdrawal of consent
- Lost to follow-up

In the event that a subject's research is prematurely terminated, or consent is withdrawn, the data collected prior to the premature termination may be used.

In the event of the subject's death, prior to the collection of consent for continuation from the subject or a family member or support person, the subject's data collected prior to death may be used.

Follow-up of subjects following discontinuation of participation in the research

In the event of premature termination of participation in the study, the primary endpoint (survival to hospital arrival with effective spontaneous cardiac activity) and secondary endpoints will be assessed if available at the time of premature termination.

Discontinuation of a subject's participation will not change their usual management of their disease.

In the event of serious adverse events during premature discontinuation of treatment and patient participation in the research, these should be notified by the investigator to the sponsor. The notification of the serious adverse event will be sent by email ([eig-vigilance.drc@aphp.fr](mailto:eig-vigilance.drc@aphp.fr)) to the sponsor. The serious adverse event will be followed up until its resolution. The independent monitoring committee may specify and/or validate the follow-up procedures.

Arrangements for replacing these subjects, if necessary

In the event of premature termination of the research due to withdrawal of consent, and in the event that the patient or his/her relatives explicitly refuse the use of all his/her data, the patient will be excluded from the analysis and replaced.

Discontinuation of part or all of the research

The AP-HP sponsor or the Competent Authority (ANSM) may prematurely interrupt all or part of the research, following the recommendations of an Independent Monitoring Committee in the following situations

- firstly, in the event of unexpected serious adverse reactions (SUSARS) requiring a reassessment of the benefit/risk ratio of the research;

- in case of intermediate analysis: discontinuation for demonstration of treatment ineffectiveness.

Similarly, unforeseen events, new information relating to the product, in the light of which the objectives of the research are unlikely to be achieved, may lead the AP-HP sponsor or the Competent Authority (ANSM) to prematurely interrupt the research.

The AP-HP sponsor reserves the right to definitively suspend the inclusions, at any time, if it appears that the inclusion objectives have not been achieved.

In case of premature termination of the research for safety reasons, the decision and the justification are transmitted by the AP-HP sponsor within 15 days to the Competent Authority (ANSM) and to the CPP, together with the recommendations of the Independent Monitoring Committee in the case of a substantial modification.

# 7. TREATMENT OF RESEARCH SUBJECTS

### Description of the investigational medicinal product(s)

# 7.1. Investigational drug 1

# The investigational drug is potassium chloride:

# - dosage: 20 ml ampoule of 10% KCl solution for infusion

# - dosage: 20 mmol of potassium chloride or 14.9 ml of 10% KCl, dosage rounded to 15 ml

# - duration of treatment: a few seconds, corresponding to the IVD injection

# - modalities and route of administration: direct intravenous administration (IVD) flash

# - packaging:

# The sponsor will provide the experimental treatments in the form of boxes of 1 ampoule of 20 ml of 10% KCl. This patient box will also contain all the devices (syringe, needle) necessary for the administration of the treatment in emergency; the boxes are labelled with the regulatory mentions of the research by the Clinical Trial Department of AGEPS and do not require specific storage conditions.

# 7.2. Description of the auxiliary treatment(s) (medicines needed to carry out the research)

# Apart from potassium chloride, no other medication specifically useful for the research is planned in this study.

# The only other drugs used will be those that can usually be administered in the management of a refractory VF OHCA, in particular adrenaline by direct intravenous route at a dosage of 1 mg, amiodarone by direct intravenous route at a dosage of 300 mg then 150 mg, then by continuous IV at a dosage of 900 mg/d, and finally lidocaine by direct intravenous route at a dosage of 1 mg/kg.

# 7.3. Description of the traceability elements that accompany the investigational drug(s)

# Dispensing will be carried out by the hospital pharmacies and a supply will be made available in the departments.

# A traceability sticker will allow the PUI to trace the allocation of the box in the endowment.

# A traceability sticker will enable the allocation of the box to the patient on his prescription to be traced.

# 7.4. Authorised and prohibited treatments (medicinal, auxiliary, surgical), including emergency medicines

# All other drugs normally used in the management of refractory OHCA are permitted.

# There are no prohibited drugs.

# 7.5. Methods of monitoring compliance

# The methods of dispensing KCl will be collected in the eCRF: volume administered in full or not, possible difficulties during injection.

**8. SAFETY ASSESSMENT - RISKS AND CONSTRAINTS ADDED BY RESEARCH**

**8.1. Procedures for recording and reporting adverse events**

Definitions

According to article R.1123-46 of the Public Health Code:

- Adverse event:

Any harmful event occurring in a person who undergoes research involving the human person, whether or not this event is related to the research or to the product to which this research relates.

- Adverse event:

An adverse event occurring in a person who is a subject of research involving humans, where that event is related to the research or the product to which the research relates.

- Adverse reaction to an investigational medicinal product:

Any noxious and unintended response to an investigational medicinal product, regardless of the dose administered.

- Serious adverse event or reaction:

Any adverse event or reaction that results in death, is life-threatening to the research subject, requires hospitalisation or prolongation of existing hospitalisation, results in persistent or significant disability or handicap, or is a congenital anomaly or malformation, regardless of the dose administered.

- Unexpected adverse reaction to an investigational medicinal product:

Any adverse reaction to the product, the nature, severity, frequency or course of which is not consistent with the reference safety information given in the summary of product characteristics or in the Investigator's Brochure when the product is not authorised.

According to Article R.1123-46 of the Public Health Code and the Notice to sponsors of clinical trials of medicinal products (ANSM):

- New fact:

Any new data that may lead to a reassessment of the benefit/risk ratio of the research or of the product that is the subject of the research, to changes in the use of this product, in the conduct of the research, or in the documents relating to the research, or to the suspension or interruption or modification of the protocol of the research or similar research.

For trials involving the first administration or use of a health product in individuals without a medical condition: any serious adverse reaction.

Examples:

(a) any clinically significant increase in the incidence of an expected serious adverse reaction;

(b) suspected unexpected serious adverse events in participants who have completed the trial, which are reported by the investigator to the sponsor, and any follow-up reports;

(c) any new facts concerning the conduct of the clinical trial or the development of the investigational medicinal product, where such new facts may affect the safety of the participants

(d) the recommendations of the Independent Monitoring Committee (IMC), if any, if they are relevant to the safety of individuals

(e) any unexpected serious adverse reaction reported to the sponsor by another sponsor of a clinical trial conducted in a third country involving the same medicinal product.

**8.2. Roles of the investigator**

The investigator should assess the seriousness of each adverse event and report all serious and non-serious adverse events in the e-CRF.

The investigator should document serious adverse events to the best of his/her ability and provide a definitive medical diagnosis whenever possible.

The investigator should evaluate the intensity of the adverse events using generalist terms:

o Mild: tolerated by the patient, not interfering with daily activities,

o Moderate: sufficiently uncomfortable to interfere with daily activities,

o Severe: which prevents daily activities.

The investigator should assess the causal relationship of serious adverse events to the investigational medicinal product.

The method used by the investigator, based on the WHO Uppsala Monitoring Centre method, is based on the following 4 causality terms:

- Certain,

- Probable/plausible,

- Possible,

- Unlikely (not excluded).

Their definition is presented in the following table (extracted from WHO-UMC causality categories, version of 17/04/2012).

Table: WHO-UMC causality categories (extract).

| **Causality term** | Assessment criteria* |
| --- | --- |
| **Certain** | - Event or laboratory test abnormality, with plausible time relationship to drug intake ** - Cannot be explained by disease or other drugs - Response to withdrawal plausible (pharmacologically, pathologically) - Event definitive pharmacologically or phenomenologically (i.e. an objective and specific medical disorder or a recognized pharmacological phenomenon) - Rechallenge satisfactory, if necessary |
| **Probable / Likely** | - Event or laboratory test abnormality, with reasonable time relationship to drug intake** - Unlikely to be attributed to disease or other drugs - Response to withdrawal clinically reasonable - Rechallenge not required |
| **Possible** | - Event or laboratory test abnormality, with reasonable time relationship to drug intake ** - Could also be explained by disease or other drugs - Information on drug withdrawal may be lacking or unclear |
| **Unlikely** | - Event or laboratory test abnormality, with a time to drug intake ** - that makes a relationship improbable (but not impossible) - Disease or other drugs provide plausible explanations |

*All points should be reasonably complied with

** Or study procedures

**Serious adverse events requiring immediate notification by the investigator to the sponsor**

According to Article R.1123-49 of the French Public Health Code, the investigator must notify the sponsor without delay from the day on which he/she becomes aware of all serious adverse events occurring in the course of research mentioned in Article L.1121-1, except those listed in the protocol and, where applicable, in the investigator's brochure as not requiring notification.

A serious adverse event presents one of the following criteria:

1. event that results in death,
2. event that endangers the life of the person who is the subject of the research,
3. event that requires hospitalisation or prolongation of hospitalisation,
4. an event that results in a significant or lasting disability or handicap,
5. an event that results in a congenital anomaly or malformation

### Protocol specifics

### Other events requiring immediate notification by the investigator to the sponsor

### - In utero exposure

### Any pregnancy discovered during the course of the research, even if it is not associated with an adverse event, must be notified to the sponsor by the investigator without delay from the day he/she becomes aware of it.

### Notification should be made in the case of maternal exposure (see RCP).

### Serious adverse events not requiring immediate notification by the investigator to the sponsor

### These SAEs will only be collected in the "adverse events" section of the case report form.

### - Natural and usual course of the disease:

### The primary endpoint is patient survival on arrival at the hospital. The mortality rate following refractory VF **OHCA** is 75-80% at hospital discharge [7]. Therefore, SAEs occurring after hospital admission will not be reported without delay.

### Similarly, deaths will not be notified to the sponsor without delay but will be recorded in the case report book. An extraction of deaths from the observation book will be carried out every 3 months by the clinical research unit and will be sent, without the need for a prior request, via the project referent of the Promotion Unit to the Vigilance sector and to the members of the IHC.

### - Adverse events likely to be linked to treatments prescribed as part of the care during the research follow-up

### These adverse events must be notified by the investigator to the regional pharmacovigilance centre on which he/she depends.

### Period of immediate reporting of SAEs by the investigator to the sponsor

### The investigator should report serious adverse events as defined in the relevant section to the sponsor without delay:

### - from the date of initiation of treatment with the investigational medicinal product,

### - until admission to hospital,

### - without time limit, when the SAE is likely to be due to the investigational medicinal product.

### Procedures and deadlines for notifying the sponsor

### The initial notification of an adverse event is the subject of a written report signed by the investigator using an adverse event notification form specific to the research and provided for this purpose (in the observation booklet).

### Each item in this document must be completed by the investigator to enable the sponsor to carry out a relevant analysis.

### The initial notification of a serious adverse event to the sponsor should be followed promptly by a detailed written follow-up report(s) to monitor the progress of the case or to supplement the information.

### The investigator should, as far as possible, transmit any document that may be useful to the sponsor (medical reports, biological results, results of additional examinations, etc.). These documents must be made anonymous. In addition, they must be completed with the following information: research acronym, participant number and initials.

### Any adverse event will be followed up until its complete resolution (stabilisation at a level deemed acceptable by the investigator or return to the previous state) even if the participant has left the research.

The initial notification, the SAE follow-up reports and any other document will be sent to the sponsor represented by its Vigilance Sector, by e-mail (eig-vigilance.drc@aphp.fr). It should be noted that it is possible to send the SAEs to the Vigilance sector by fax to +33 (0)1 44 84 17 99 only in the event of an unsuccessful attempt to send the SAEs by e-mail (in order to avoid duplication).

In the case of this research with e-CRF:

- the investigator completes the SAE notification form in the e-CRF, validates it, prints it, signs it and sends it by e-mail;

- if the connection to the e-CRF is not possible, the investigator completes, signs and sends the SAE notification form to the Vigilance section. As soon as the connection is re-established, the investigator should complete the SAE notification form in e-CRF.

The investigator must respond to any request for additional information from the sponsor.

For any questions relating to the notification of an adverse event, the Vigilance Sector can be contacted by e-mail: vigilance.drc@aphp.fr.

In the case of in utero exposure, the investigator must complete the "Form for the notification and follow-up of a pregnancy that occurred during research".

The investigator should follow the pregnant woman through the pregnancy or termination of the pregnancy and notify the sponsor of the outcome with this form.

If the outcome of the pregnancy falls within the definition of serious adverse events (spontaneous abortion, termination of pregnancy, foetal death, congenital anomaly, etc.), the investigator should follow the reporting procedures for SAEs.

Initial pregnancy notification, follow-up reports of SAEs and any other documents should be sent to the sponsor in the same way as above.

Roles of the promoter

The sponsor, represented by its Vigilance Sector, assesses the safety of each investigational medicinal product on an ongoing basis, throughout the research.

**Analysis and reporting of serious adverse events**

The sponsor assesses:

- the seriousness of all adverse events reported to him.

- their causal relationship to each investigational medicinal product and to any other treatments.

- All serious adverse events for which the investigator and/or sponsor believe that a causal relationship with the investigational medicinal product can reasonably be considered are considered as suspected serious adverse events.

- The expected or unexpected nature of the serious adverse reactions.

- Any serious adverse reaction whose nature, severity, frequency or course is not consistent with the reference safety information given in the summary of product characteristics or in the Investigator's Brochure when the product is not authorised is considered unexpected.

- The assessment of the expected/unexpected nature of a serious adverse reaction is carried out by the sponsor represented by its Vigilance Sector on the basis of the information described below.

For serious adverse events that may be related to the investigational medicinal product:

It is advisable to refer to the SPC of the product "Potassium chloride".

<http://agence-prd.ansm.sante.fr/php/ecodex/frames.php?specid=63662572&typedoc=R&ref=R0320091.htm>

- The sponsor must report any suspected serious unexpected adverse reaction (SUSAR) to the Agence nationale de sécurité des médicaments et des produits de santé (ANSM) within the regulatory timeframe:
- The initial report must be made without delay from the date of knowledge of the sponsor in the case of serious unexpected adverse reactions resulting in death or life-threatening conditions and within 15 days from the date of knowledge of the sponsor in the case of other serious unexpected adverse reactions;
- All relevant additional information should be reported by the sponsor in the form of follow-up reports, within 8 calendar days of the information becoming available to the sponsor.

Any suspected serious unexpected adverse reaction is also reported electronically to the European Medicines Agency (EMA) Eudravigilance database on adverse drug reactions.

The sponsor informs all relevant investigators of any data that could have an adverse impact on the safety of research subjects.

**Analysis and reporting of other safety data**

This is any new information that may lead to a reassessment of the risk-benefit balance of the research or of the investigational product, to changes in the use of the investigational product, in the conduct of the research, or in the documentation of the research, or to the suspension or discontinuation or modification of the protocol of the research or similar research. For trials involving the first administration or use of a health product in persons without medical conditions: any serious adverse reaction.

The sponsor shall inform the competent authority and the Data Protection Committee without delay of the new facts and, where appropriate, of the measures taken, as from the day on which he became aware of them.

Following the initial report of a new event, the sponsor shall send to the competent authorities, in the form of a follow-up report on the new event, any additional information relevant to the new event within a maximum of 8 days from the time when the information becomes available.

**Annual safety report**

The sponsor must prepare an annual Development Safety Update Report (DSUR) once a year for the duration of the research, including

- an analysis of the safety of the persons involved in the research,

- a description of the patients included in the research (demographic characteristics, etc.)

- a list of all suspected serious adverse events that occurred during the reporting period,

- summary tables of all serious adverse events that have occurred since the beginning of the research.

The report is transmitted within 60 days after the anniversary date corresponding to the date of authorisation of the research by the ANSM.

**Independent Monitoring Committee**

# An Independent Monitoring Committee (IMC) is set up by the sponsor for this research. Its main mission is to be a data safety monitoring committee. It also has the task of data monitoring.

# A preliminary meeting of the ISC is planned before the first participant is included, ideally before the protocol is submitted to the competent authority and the CPP.

# All of the tasks and the precise operating procedures of the ISC are described in the study's ISC charter.

# The members of the ISC are

# - Pr B. CHOLLEY: Anesthesia-Resuscitation-HEGP

# - Dr T. LOEB: Anaesthesia-Resuscitation-SAMU 92

# - Pr F. CANOUÏ-POITRINE: Statistician-UPEC-MONDOR

# - Pr E. WIEL: Anaesthesia-Resuscitation and Emergency Medicine-CHU LILLE

# The functioning of the ISC will be in accordance with the promoter's procedures. The ISC has an advisory function, the promoter remains the decision maker.

# 9. DATA MANAGEMENT

## 9.1. Data collection procedures

## Upon inclusion of a patient, upon return from the procedure, the emergency physician investigator will send a fax to the CRU indicating the inclusion of a subject.

## Postal, electronic, and telephone contact information for the patient and a relative will be collected and sent to the clinical study technician trained in the protocol to allow follow-up of surviving patients at 3 months.

## The commune of birth will be collected to verify the vital status of patients if they are lost to follow-up at 3 months.

## Data on the pre-hospital management of the patient will be collected by the pre-hospital emergency department team that included the patient.

## Data on the patient's in-hospital follow-up will be collected by the intensive care unit that managed the patient.

## The clinical study technician will contact surviving patients at 3 months by telephone for assessment of the CPC score.

## Pre-hospital management and hospital follow-up data will be collected in a paper case report form and entered into the eCRF.

## 9.2. Identification of data collected directly in the CRFs which will be considered as source data

## Data collected at the 3-month call will be entered directly into the eCRF.

## 9.3. Right of access to source data and documents.

Access to data

According to GCP:

- the sponsor is responsible for obtaining the agreement of all parties involved in the research to ensure direct access to all research sites, source data, source documents and reports for the purposes of quality control and audit by the sponsor, or inspection by the competent authority

- the investigators will make available to the persons responsible for monitoring, quality control or audit or inspection of interventional research involving the human person, the documents and individual data strictly necessary for this control, in accordance with the legislative and regulatory provisions in force (Articles L.1121-3 and R.5121-13 of the Public Health Code).

Source documents

The source documents, defined as any original document or object allowing the existence or accuracy of a data or fact recorded during the research to be proven, will be kept according to the regulations in force by the investigator or by the hospital in the case of a hospital medical file.

For the pre-hospital part of the study, the source document will be the pre-hospital intervention sheet (SMUR or Ambulance de Réanimation de la Brigade de Sapeurs Pompiers de Paris) and the duplicate paper CRF (1 sheet will be kept in the pre-hospital emergency service that included the patient and the original deletion will be sent by post or fax to the clinical study technician for entry).

The data collected directly in the CRF are research-specific data.

For the hospital phase, data will be collected via an eCRF. The eCRF will be completed by a clinical study technician or an investigator under the responsibility of the principal investigator of the intensive care unit receiving the patient. A medical record will be established upon admission of the patient to the hospital, and will then be considered as the source record for the collection of data during the hospital phase.

Confidentiality of data

The persons responsible for the quality control of research involving the human person (article L.1121-3 of the public health code), will take all the necessary precautions to ensure the confidentiality of information relating to the experimental medicinal products, the research, the persons involved and in particular their identity as well as the results obtained.

These persons, in the same way as the investigators themselves, are subject to professional secrecy (in accordance with the conditions defined by Articles 226-13 and 226-14 of the Penal Code).

During research involving the human person and at its conclusion, the data collected on the persons involved and transmitted to the sponsor by the investigators (or any other specialist) must be made non-identifying. Under no circumstances should the names of the individuals concerned or their addresses appear in clear text.

Only the initials of the surname and first name will be recorded, together with a coded number specific to the research indicating the order of inclusion of the subjects.

The sponsor must ensure that each person who takes part in the research has given written consent for access to individual data concerning him or her and strictly necessary for the quality control of the research.

**9.4. Data processing and storage of documents and data**

Identification of the person responsible and the place where the data processing is managed

The management of the clinical database will be under the responsibility of the URC Paris Descartes - Necker - Cochin.

**Data entry**

Data entry on the observation book will be carried out by the investigators.

Data entry will be carried out on an electronic medium via an Internet browser (eCRF, cleanWEB by the company Telemedecine technologies).

**9.5. Ownership of the data**

The AP-HP is the owner of the data and no use or transmission to a third party may be made without its prior agreement.

# 10. STATISTICAL ASPECTS

**10.1 Assumptions for calculating the number of subjects required and the result**

The experimental design chosen is a 2-step phase II design according to Simon (optimum design). This procedure allows the following hypotheses to be tested:

- H0: p≤ p0 (null hypothesis: insufficient efficacy of KCl treatment)

- H1: p> p0 (alternative hypothesis: sufficient effectiveness of KCl treatment)

The probability p0 represents the theoretical efficacy rate below which the experimental treatment is considered unattractive. One should also set a theoretical efficacy rate that one wishes to demonstrate, if it exists (p1 or target response rate).

To demonstrate 50% efficacy (p1) with a minimum efficacy of 35% (p0, observed survival of the amiodarone group in Kudenchuk [7]), it is necessary to include 81 patients, with an alpha risk of 10% and power of 90%.

34 patients will be included in the first stage. If 12 or fewer successes are observed, the trial will not be continued and it will be concluded that KCl is ineffective. If at least 13 successes are observed, the trial will continue with the inclusion of 47 new patients in the second stage.

The treatment will be considered effective, according to these assumptions, if at least 34 successes are observed in total out of the 81 patients included.

**10.2. Description of planned statistical methods including timing of planned interim analyses**

The analysis will be performed at the Clinical Research Unit Paris-Descartes Necker Cochin under the responsibility of Dr Caroline Elie using R software (http://cran.r-project.org/).

A descriptive analysis of the clinical characteristics of the patients at inclusion will first be carried out. Quantitative data will be expressed as mean ± standard deviation or median [minimum-maximum], and as numbers and percentages for qualitative data.

Analysis of the primary endpoint

The primary endpoint is the survival of the patient on arrival at the hospital (with effective spontaneous cardiac activity).

As already stated in the sample size justification, efficacy will be demonstrated at the end of stage 2 if at least 34 patients have effective spontaneous cardiac activity at hospital arrival, out of the total 81 patients included.

The percentage of successful patients will be calculated at the end of stage 1 if the number of successes is not compatible with the continuation of the study or at the end of stage 2 if it is not. The 95% confidence interval of this percentage will be estimated from an exact binomial distribution.

Analysis of secondary endpoints

Tolerance data should be described in terms of frequency and percentage of occurrence.

Similarly, other quantitative secondary endpoints should be described as mean ± standard deviation or median [minimum - maximum], and other qualitative secondary endpoints as numbers and percentages.

The survival of patients at 3 months can be described globally using Kaplan-Meier curves.

# 11. QUALITY CONTROL AND ASSURANCE

Each research project involving the human being taken on by the AP-HP is classified according to the anticipated risk incurred by the persons taking part in the research thanks to the classification of interventional research involving the human being promoted by the AP-HP

**11.1. General organisation**

The sponsor must ensure the safety and respect of the persons who have agreed to participate in the research. The sponsor must set up a quality assurance system to monitor the progress of the research in the investigating centres.

To this end, the sponsor appoints Clinical Research Associates (CRAs) whose main task is to carry out regular follow-up visits to the research sites after having carried out the opening visits.

The objectives of the research monitoring, as defined in the Good Clinical Practices, (GCP §5.18.1) are to verify that

- the rights, safety and protection of the persons undergoing the research are satisfied,

- the data reported are accurate, complete and consistent with the source documents

- the research is conducted in accordance with the applicable protocol, GCP and applicable laws and regulations.

Centre opening strategy

The strategy for opening centres for this research is determined by the appropriate monitoring plan.

The openings will be done on site.

Scope of centre monitoring

In the case of this D-risk research, the choice of an appropriate level of monitoring was weighed against the complexity, impact and budget of the research. To this end, the promoter, in agreement with the coordinating investigator, determined the logistical and impact score which made it possible to obtain the level of monitoring to be implemented on the research: high level.

**11.2. Quality control**

A Clinical Research Associate (CRA) mandated by the sponsor will ensure that the research is carried out correctly, that the data generated are collected in writing, documented, recorded and reported, in accordance with the Standard Operating Procedures implemented within the DRCI and in compliance with Good Clinical Practice as well as with the legislative and regulatory provisions in force.

The investigator and the members of his/her team agree to be available for quality control visits carried out at regular intervals by the Clinical Research Associate. During these visits, the following elements will be reviewed according to the level of monitoring

- written consent;

- compliance with the research protocol and the procedures defined therein

- quality of the data collected in the observation book: accuracy, missing data, consistency of the data with the "source" documents

- management of the treatments used.

**11.3. Observation book**

All the information required by the protocol must be recorded in the observation books and an explanation must be given for each missing item of data. Data should be collected as they are obtained and transcribed into these notebooks in a neat and legible manner.

Erroneous data recorded in the case report forms should be crossed out and the new data should be copied next to the crossed-out information, accompanied by initials, date and possibly a justification by the investigator or authorised person who made the correction.

Filling in the case report form via the internet by the investigator and/or a clinical study technician allows the CRA to view the data quickly and remotely. The investigator is responsible for the accuracy, quality and relevance of all data entered. In addition, as data is entered, it is immediately checked for consistency. As such, the investigator must validate any change in value in the CRF. These changes are subject to an audit trail. A justification may be included as a comment.

A paper printout will be requested at the end of the study, authenticated (dated and signed) by the investigator. A copy of the authenticated document for the sponsor must be archived by the investigator.

**11.4. Management of non-compliance**

Any event arising from non-compliance with the protocol, standard operating procedures, good clinical practice or applicable laws and regulations by an investigator or any other person involved in the conduct of the research must be reported to the sponsor.

Such non-compliance will be managed in accordance with the sponsor's procedures.

**11.5. Audit / inspections**

Investigators agree to accept quality assurance audits by the sponsor and inspections by the competent authorities. All data, documents and reports are subject to audit and regulatory inspection without prejudice to medical confidentiality.

An audit may be carried out at any time by persons mandated by the sponsor and independent of the research managers. Its purpose is to ensure the quality of the research, the validity of its results and compliance with the law and regulations in force.

The persons conducting and supervising the research agree to comply with the requirements of the sponsor and the competent authority with regard to an audit or inspection of the research.

The audit may apply to all stages of the research, from the development of the protocol to the publication of the results and the classification of the data used or generated in the research.

**11.6. Undertaking of responsibilities of the Principal Investigator**

Prior to the commencement of the research, each investigator will provide the research sponsor's representative with an up to date, signed personal curriculum vitae (CV) that is less than one year old and includes his/her RPPS number. The CV should include previous research involvement and training related to clinical research.

Each investigator will undertake to comply with the obligations of the law and to conduct the research in accordance with GCP, respecting the terms of the Declaration of Helsinki in force.

The principal investigator of each participating centre will sign a commitment of responsibility (DRCI type document) which will be given to the sponsor's representative.

The investigators and their collaborators will sign a delegation of duties form specifying the role of each and will provide their CVs.

# 12. ETHICAL AND LEGAL ASPECTS

**12.1. Procedures for informing and obtaining the consent of persons undergoing research**

In accordance with article L1122-1-3 of the CSP, in the case of research involving the human person to be carried out in emergency situations which do not allow the prior consent of the person who will be subjected to it to be obtained, the protocol presented for the opinion of the CPP (Comité de Protection des Personnes) stipulates that the consent of this person is not sought and that only the consent of the members of his or her family or that of the trusted person mentioned in article L. 1111-6 under the conditions provided for in article L. 1122-1-1, if they are present, is sought.

The protocol provides for a derogation from this obligation in the case of an immediate life-threatening emergency, which is the case in our study. This exemption is therefore requested from the CPP. The patient, or if applicable, the family members or the trusted person will be informed as soon as possible, and his/her/their consent will be requested for the possible continuation of this research. They may also object to the use of the person's data in this research.

In accordance with Article 57 of Law No. 2018-493 of 20 June 2018 on the protection of personal data, information concerning deceased persons may be subject to data processing, unless the patient has, during his/her lifetime, expressed his/her refusal.

A copy of the information document and consent form dated and signed by the person undergoing the research, or a family member if applicable, and by the principal investigator or the physician representing him or her, is given to the person or a family member if applicable. The principal investigator or his or her medical representative should retain a copy.

One copy should be placed at the end of the study in a sealed tamper-proof envelope containing all the consent forms, which should be archived by the sponsor.

In addition, the investigator must specify in the patient's medical record the patient's participation in the research, the procedures for obtaining consent, and the procedures for providing the information required to obtain it. He/she will keep a copy of the form for collecting the person's consent, dated and signed.

The patient will be informed that if there is no response to the call within 3 months, the commune of birth may be contacted in order to collect the vital status (RNIPP / CépiDC)

**12.2. Prohibition for the person to participate in other research or period of exclusion foreseen at the end of the research, if applicable**

During the period of participation, the subject may not participate in another interventional research protocol involving the human being.

At the end of the subject's participation, there is no exclusion period.

**12.3. Authorisation of sites**

The research takes place in non-hospital sites or health care services on persons with a clinical condition for which the services are competent and which requires procedures usually performed in the course of their activities. Therefore, it is not necessary to have a specific site authorisation for the research.

**12.4. Legal obligations**

Role of the sponsor

The Assistance Publique - Hôpitaux de Paris (AP-HP) is the promoter of this research and, by delegation, the Délégation à la Recherche Clinique et à l'Innovation (DRCI) carries out its missions, in accordance with Article L.1121-1 of the Public Health Code. Assistance Publique - Hôpitaux de Paris reserves the right to interrupt the research at any time for medical or administrative reasons; in this eventuality, a notification will be provided to the investigator

Request for an opinion from the CPP

The AP-HP, as the sponsor, obtains a favourable opinion from the CPP concerned for research involving the human being and involving a medicinal product for human use, prior to its implementation, within the framework of its competences and in accordance with the legislative and regulatory provisions in force.

Request for authorisation from the ANSM

The AP-HP as promoter obtains for research involving the human person relating to a medicinal product for human use, prior to its implementation, the authorisation of the ANSM, within the framework of its competence and in accordance with the legislative and regulatory provisions in force.

Procedures relating to data protection regulations

The computer file used for this research is implemented in accordance with French (amended Data Protection Act) and European (General Data Protection Regulation - GDPR) regulations.

This research does not fall within the framework of the "Reference Methodology" (MR-001) of the CNIL because the inclusion is done within the framework of an emergency without collecting consent at inclusion.

Prior to the implementation of the data processing necessary to carry out the research, the sponsor must obtain the authorisation of the CNIL.

Modifications to the research

Any substantial modification of the protocol by the coordinating investigator must be sent to the sponsor for approval. After this approval, the sponsor must obtain a favourable opinion from the CPP and an authorisation from the ANSM within the framework of their respective competences before implementation.

The information note and the consent form may be revised if necessary, particularly in the event of substantial modification of the research or the occurrence of adverse effects.

Final report of the research

The final report of the research involving the human person mentioned in article R1123-67 of the CSP is drawn up and signed by the sponsor and the investigator. A summary of the report drawn up in accordance with the reference plan of the competent authority must be sent to the competent authority within one year of the end of the research, corresponding to the end of the participation of the last person who takes part in the research.

Archiving

The specific documents of an interventional research involving the human being and a medicinal product for human use shall be archived by the investigator and the sponsor for a period of 15 years after the end of the research.

This indexed archiving includes

- A sealed envelope for the investigator containing a copy of all the information notes and consent forms signed by all persons at the centre who participated in the research;

- A sealed envelope for the sponsor containing a copy of all the information notes and consent forms signed by all the people at the centre who took part in the research;

- Research binders for the Investigator and the sponsor including (but not limited to)

- the successive versions of the protocol (identified by the version number and date), its appendices,

- ANSM authorisations and CPP opinions,

- correspondence letters,

- the inclusion list or register,

- the annexes specific to the research,

- the final report of the research.

- Data collection documents

**13. FUNDING AND INSURANCE**

**13.1. Source of funding**

Ministry of Health - PHRC inter-regional 2018

**13.2. Insurance**

The Promoter takes out insurance for the entire duration of the research, covering its own civil liability as well as that of any doctor involved in the research. It also ensures full compensation for the harmful consequences of the research for the person who takes part in it and his/her beneficiaries, unless it can prove that the damage is not attributable to its fault or to that of any other party involved, without the possibility of invoking the act of a third party or the voluntary withdrawal of the person who had initially agreed to take part in the research

The Assistance Publique - Hôpitaux de Paris (AP-HP) has taken out insurance with the company HDI-GLOBAL SE through BIOMEDIC-INSURE for the entire duration of the research, guaranteeing its civil liability as well as that of any participant (doctor or staff involved in the research), in accordance with article L.1121-10 of the CSP.

# 14. RULES FOR PUBLICATION

The APHP must be mentioned in the affiliations of the author(s) of the publications resulting from this research and mention the AP-HP promoter (DRCI) and the source of funding (see below for the modalities of affiliation and mention of the promoter and funder).

**14.1 Mention of the AP-HP affiliation for projects promoted by the AP-HP**

If an author has several affiliations, the order in which the institutions are cited (AP-HP, University, INSERM...) is not important. Each of these affiliations must be identified by an address separated by a semicolon (;). The institution AP-HP must appear under the acronym "AP-HP" first in the address followed precisely by: AP-HP, hospital, department, city, postal code, France.

**14.2 Mention of the AP-HP sponsor (DRCI) in the acknowledgments of the manuscript**

"The sponsor was Assistance Publique - Hôpitaux de Paris (Délégation à la Recherche Clinique et à l'Innovation)".

**14.3 Mention of the funder in the acknowledgments of the manuscript**

"The study was funded by a grant from Programme Hospitalier de Recherche Clinique - PHRC-IR 2018 (Ministry of Health)".

This research is registered on the website http://clinicaltrials.gov/ under the registration number (add the registration number when the research is registered).

# 15. bibliographY

| **1** | Gueugniaud PY, Bertrand C, Savary D, Hubert H. L’arrêt cardiaque en France : pourquoi un registre national ? Presse Med 2011;40(6):634-8. |
| --- | --- |
| **2** | Link MS, Berkow LC, Kudenchuk PJ, et al. Part 7: Adult Advanced Cardiovascular Life Support: 2015 American Heart Association Guidelines Update for Cardiopulmonary Resuscitation and Emergency Cardiovascular Care. Circulation 2015 Nov 3;132(18 Suppl 2):S444-64. |
| **3** | Sasson C, Rogers MA, Dahl J, Kellermann AL. Predictors of survival from out-of-hospital cardiac arrest: a systematic review and meta-analysis. Circ Cardiovasc Qual Outcomes 2010 ; 3 : 63-81. |
| **4** | Daya MR, Schmicker RH, Zive DM, et al ; Resuscitation Outcomes Consortium Investigators. Out-of-hospital cardiac arrest survival improving over time: Results from the Resuscitation Outcomes Consortium (ROC). Resuscitation 2015 ; 91 : 108-15. |
| **5** | American Heart Association. [2015 American Heart Association Guidelines Update for Cardiopulmonary Resuscitation and Emergency Cardiovascular Care](http://circ.ahajournals.org/content/132/18_suppl_2/S315). Circulation 2015 ; 132 : S 315-S589. |
| **6** | European Resuscitation Council. European Resuscitation Council Guidelines for Resuscitation 2015. Resuscitation 2015, 95 : 1-311. |
| **7** | Kudenchuk PJ, Brown SP, Daya M, et al.; Resuscitation Outcomes Consortium Investigators. Amiodarone, Lidocaine, or Placebo in Out-of-Hospital Cardiac Arrest. N Engl J Med 2016 May 5;374(18):1711-22. |
| **8** | Menasché P. Protection myocardique. In : Janvier G, Lehot JJ. Circulation extracorporelle : principes et pratique. Rueil-Malmaison, France : Arnette, 2000 : 77-96. |
| **9** | Weinstock L, Clark JH. Successful treatment of ventricular fibrillation with intracardiac potassium chloride. Am J Cardiol 1961 May;7:742-5. |
| **10** | Robicsek F. Biochemical termination of sustained fibrillation occurring after artificially induced ischemic arrest. J Thorac Cardiovasc Surg 1984 Jan;87(1):143-5. |
| **11** | Øvrum E, Tangen G, Holen EA, Ringdal MA, Istad R. Conversion of postischemic ventricular fibrillation with intraaortic infusion of potassium chloride. Ann Thorac Surg 1995 Jul;60(1):156-9. |
| **12** | Almdahl SM, Damstuen J, Eide M, Mølstad P, Halvorsen P, Veel T. Potassium-induced conversion of ventricular fibrillation after aortic declamping. Interact Cardiovasc Thorac Surg 2013 Feb;16(2):143-50. |
| **13** | Liakopoulos OJ, Allen BS, Buckberg GD, Hristov N, Tan Z, Villablanca JP, Trummer G. Resuscitation after prolonged cardiac arrest: role of cardiopulmonary bypass and systemic hyperkalemia. Ann Thorac Surg 2010 Jun;89(6):1972-9. |
| **14** | Watanabe G, Yashiki N, Tomita S, Yamaguchi S. Potassium-induced cardiac resetting technique for persistent ventricular tachycardia and fibrillation after aortic declamping. Ann Thorac Surg 2011 Feb;91(2):619-20. |
| **15** | Jouffroy R, Lamhaut L, Philippe P, An K, Carli P, Vivien B. A new approach for treatment of refractory ventricular fibrillation allowed by extra corporeal life support (ECLS)? Resuscitation 2014 Aug;85(8):e118. |
| **16** | Koller ML, Riccio ML, Gilmour RF Jr. Effects of [K(+)](o) on electrical restitution and activation dynamics during ventricular fibrillation. Am J Physiol Heart Circ Physiol. 2000 Dec;279(6):H2665-72. |
| **17** | Pandit SV, Warren M, Mironov S, Tolkacheva EG, Kalifa J, Berenfeld O, Jalife J. Mechanisms underlying the antifibrillatory action of hyperkalemia in Guinea pig hearts. Biophys J. 2010 May 19;98(10):2091-101. |
| **18** | Almdahl SM, Veel T, Eide M, Damstuen J, Halvorsen P, Mølstad P. Postcardioplegia ventricular fibrillation: no impact on subsequent survival. Scand Cardiovasc J 2014 Aug;48(4):249-54. |
| **19** | Houiller P, Paillard M. Régulation du métabolisme du potassium. Désordres acido-basiques et hydro-électrolytiques. Paris : Arnette ; 1997. p. 221-51. |
| **20** | Brenner BM, Berliner RW. The transport of potassium. In: Orloff J, Berliner RW, eds Renal Physiology, handbook of physiology. Bethesda: American Physiological Society; 1973. p. 497-519. |
| **21** | Garcia P, Belhoula M, Grimaud D. Les dyskaliémies. Consensus d’actualisation SFAR 1999. |
| **22** | Paillard M, Houiller P. Bilan de potassium et kaliémie. In : Paillard M, éd. Physiologie rénale et désordres électrolytiques. Paris : Hermann ; 1992. p. 153-83. |

# 16. LIST OF ADDENDA

# List of investigators

| **N° centre** | **Contact details of the research location** | **Title** | **First name - Name** | **Phone / e-mail / Fac simile** |
| --- | --- | --- | --- | --- |
| 001 | SAMU de Paris – SMUR Necker | Pr | Benoît VIVIEN | Tél : 01 44 49 23 67  Mail : [benoit.vivien@aphp.fr](mailto:benoit.vivien@aphp.fr)  Fax : 01 44 49 23 25 |
| 002 | SAMU de Paris – SMUR Pitié Salpêtrière | Dr | Patrick ECOLLAN | Tél : 01 42 16 76 60  Mail : [patrick.ecollan@aphp.fr](mailto:patrick.ecollan@aphp.fr)  Fax : 01 42 16 76 76 |
| 003 | Brigade de Sapeurs Pompiers de Paris | Pr | Bertrand PRUNET | Tél : 01 56 79 67 53  Mail : [bertrand.prunet@pompiersparis.fr](mailto:bertrand.prunet@pompiersparis.fr)  Fax : 01 56 79 67 67 |
| 004 | SAMU 94 – SMUR Henri Mondor | Dr | Charlotte CHOLLET-XEMARD | Tél : 01 45 17 95 39  Mail  [charlotte.chollet@aphp.fr](mailto:charlotte.chollet@aphp.fr)  Fax : 01 45 17 95 30 |
| 010 | Service de Médecine Intensive et Réanimation – Hôpital Cochin | Pr | Alain CARIOU | Tél : 01 58 41 25 01  Mail : [alain.cariou@aphp.fr](mailto:alain.cariou@aphp.fr)  Fax : 01 58 41 25 05 |
| 011 | Service de Médecine Intensive et Réanimation - Hôpital Pitié Salpêtrière | Pr | Alain COMBES | Tél : 01 42 16 38 18  Mail : [alain.combes@aphp.fr](mailto:alain.combes@aphp.fr)  Fax : 01 42 16 38 17 |
| 012 | Service de Médecine Intensive et Réanimation EOLE - Hôpital Pitié Salpêtrière | Pr | Alexandre DEMOULE | Tel : 01 42 16 78 58  Mail : [alexandre.demoule@aphp.fr](mailto:alexandre.demoule@aphp.fr)  Fax : 01 42 16 78 43 |
| 013 | Service de Médecine Intensive et Réanimation et toxicologique - Hôpital Lariboisière | Pr | Bruno MEGARBANE | Tel : 06 60 22 18 04  Mail : bruno.megarbane@aphp.fr  Fax : 01 49 95 65 78 |
| 014 | Service de Médecine Intensive et Réanimation - Hôpital Bichat | Pr | Jean-François TIMSIT | Tel : 01 40 25 77 02  Mail : [jean-francois.timsit@aphp.fr](mailto:jean-francois.timsit@aphp.fr)  Fax : 01 40 25 88 37 |
| 015 | Service de Médecine Intensive et Réanimation - Hôpital Ambroise Paré | Pr | Antoine VIEILLARD-BARON | Tel : 01 49 09 58 92  Mail : [antoine.vieillard-baron@aphp.fr](mailto:antoine.vieillard-baron@aphp.fr)  Fax : 01 49 09 58 92 |
| 016 | Service de Médecine Intensive et Réanimation - Hôpital Européen Georges Pompidou | Pr | Jean-Luc DIEHL | Tel : 01 56 09 32 01  Mail : [jean-luc.diehl@aphp.fr](mailto:jean-luc.diehl@aphp.fr)  Fax : 01 56 09 32 02 |
| 017 | Service de Médecine Intensive et Réanimation - Hôpital Bicêtre | Pr | Christian RICHARD | Tel : 01 45 21 35 45  Mail : [christian.richard@aphp.fr](mailto:christian.richard@aphp.fr)  Fax : 01 45 21 35 51 |
| 018 | Service de Médecine Intensive et Réanimation - Hôpital Henri Mondor | Pr | Armand MEKONTSO DESSAP | Tel : 01 49 81 23 94  Mail : [armand.dessap@aphp.fr](mailto:armand.dessap@aphp.fr)  Fax : 01 49 81 49 43 |

## 16.2. CPC score scale

CPC score scale, according to Ajam et al. Scand J Trauma Resusc Emerg Med 2011 (doi : [10.1186/1757-7241-19-38](https://dx.doi.org/10.1186%2F1757-7241-19-38))
