# Supplementary material for: Evaluation of the effectiveness of potassium chloride in the management of out-of hospital cardiac arrest by refractory ventricular fibrillation: Study protocol of the POTACREH study
Source: PLoS One. 2023 Apr 12;18(4):e0284429. doi: 10.1371/journal.pone.0284429 (PMC10096226; doi:10.1371/journal.pone.0284429)
Supplement: Attachment — Submitted filename: Responses to reviewers Plos One.docx [file pone.0284429.s004.docx]

Journal Requirements:

When submitting your revision, we need you to address these additional requirements.

1. Please ensure that your manuscript meets PLOS ONE's style requirements, including those for file naming. The PLOS ONE style templates can be found at 
<https://journals.plos.org/plosone/s/file?id=wjVg/PLOSOne_formatting_sample_main_body.pdf> and 
<https://journals.plos.org/plosone/s/file?id=ba62/PLOSOne_formatting_sample_title_authors_affiliations.pdf>

**Answer: the revised version of the manuscript meets PLOS ONE's style requirements**

2. During your revisions, please note that a simple title correction is required: please add 'study protocol' to your title, i.e. Evaluation of the effectiveness of potassium chloride in the management of out-of hospital cardiac arrest by refractory ventricular fibrillation: study protocol of the POTACREH Study. Please ensure this is updated in the manuscript file and the online submission information.

**Answer: in the revised version of the manuscript, the title was modified accordingly in the manuscript file and on the online submission.**

3. Thank you for stating the following financial disclosure: 
"The funders had and will not have a role in study design, data collection and analysis, decision to publish, or preparation of the manuscript."

**Answer: in the revised version of the manuscript, the requested financial disclosure was added.**

At this time, please address the following queries:

a) Please clarify the sources of funding (financial or material support) for your study. List the grants or organizations that supported your study, including funding received from your institution. 
b) State what role the funders took in the study. If the funders had no role in your study, please state: “The funders had no role in study design, data collection and analysis, decision to publish, or preparation of the manuscript.”
c) If any authors received a salary from any of your funders, please state which authors and which funders.
d) If you did not receive any funding for this study, please state: “The authors received no specific funding for this work.”

Please include your amended statements within your cover letter; we will change the online submission form on your behalf.

**Answer: all previous queries were corrected in the revised version of the manuscript and in the cover letter.**

4. Thank you for stating the following in the Funding Section of your manuscript: 
"The « POTACREH » trial is funded by the French Ministry of Health (Ministère des Solidarités et de la Santé) through the 2018 Inter Regional Clinical Research Program (Programme Hospitalier de Recherche Clinique Inter Régional). The funder has no influence on the recruitment and on care delivered to patients."

We note that you have provided funding information that is not currently declared in your Funding Statement. However, funding information should not appear in the Acknowledgments section or other areas of your manuscript. We will only publish funding information present in the Funding Statement section of the online submission form. 
Please remove any funding-related text from the manuscript and let us know how you would like to update your Funding Statement. Currently, your Funding Statement reads as follows: 
"The funders had and will not have a role in study design, data collection and analysis, decision to publish, or preparation of the manuscript."

Please include your amended statements within your cover letter; we will change the online submission form on your behalf.

**Answer: All above requested modifications have been done in the revised version of the manuscript.**

5. Your ethics statement should only appear in the Methods section of your manuscript. If your ethics statement is written in any section besides the Methods, please move it to the Methods section and delete it from any other section. Please ensure that your ethics statement is included in your manuscript, as the ethics statement entered into the online submission form will not be published alongside your manuscript.

**Answer: In the revised version of the manuscript, ethics statement appears in the Methods section.**

6. Please review your reference list to ensure that it is complete and correct. If you have cited papers that have been retracted, please include the rationale for doing so in the manuscript text, or remove these references and replace them with relevant current references. Any changes to the reference list should be mentioned in the rebuttal letter that accompanies your revised manuscript. If you need to cite a retracted article, indicate the article’s retracted status in the References list and also include a citation and full reference for the retraction notice.

**Answer: The reference list has been reviewed and is complete and correct.**

Reviewers' comments:

Reviewer's Responses to Questions

**Comments to the Author**

1. Does the manuscript provide a valid rationale for the proposed study, with clearly identified and justified research questions?

The research question outlined is expected to address a valid academic problem or topic and contribute to the base of knowledge in the field.

Reviewer #1: Yes

Reviewer #2: Yes

2. Is the protocol technically sound and planned in a manner that will lead to a meaningful outcome and allow testing the stated hypotheses?

The manuscript should describe the methods in sufficient detail to prevent undisclosed flexibility in the experimental procedure or analysis pipeline, including sufficient outcome-neutral conditions (e.g. necessary controls, absence of floor or ceiling effects) to test the proposed hypotheses and a statistical power analysis where applicable. As there may be aspects of the methodology and analysis which can only be refined once the work is undertaken, authors should outline potential assumptions and explicitly describe what aspects of the proposed analyses, if any, are exploratory.

Reviewer #1: Yes

Reviewer #2: Yes

3. Is the methodology feasible and described in sufficient detail to allow the work to be replicable?


Descriptions of methods and materials in the protocol should be reported in sufficient detail for another researcher to reproduce all experiments and analyses. The protocol should describe the appropriate controls, sample size calculations, and replication needed to ensure that the data are robust and reproducible.

Reviewer #1: Yes

Reviewer #2: Yes

4. Have the authors described where all data underlying the findings will be made available when the study is complete?

The [PLOS Data policy](https://journals.plos.org/plosone/s/materials-and-software-sharing) requires authors to make all data underlying the findings described in their manuscript fully available without restriction, with rare exception, at the time of publication. The data should be provided as part of the manuscript or its supporting information, or deposited to a public repository. For example, in addition to summary statistics, the data points behind means, medians and variance measures should be available. If there are restrictions on publicly sharing data—e.g. participant privacy or use of data from a third party—those must be specified.

Reviewer #1: No

Reviewer #2: Yes

5. Is the manuscript presented in an intelligible fashion and written in standard English?

PLOS ONE does not copyedit accepted manuscripts, so the language in submitted articles must be clear, correct, and unambiguous. Any typographical or grammatical errors should be corrected at revision, so please note any specific errors here.

Reviewer #1: Yes

Reviewer #2: Yes

**Answer: We thank the reviewers for their positive comments.**

6. Review Comments to the Author

Please use the space provided to explain your answers to the questions above and, if applicable, provide comments about issues authors must address before this protocol can be accepted for publication. You may also include additional comments for the author, including concerns about research or publication ethics.

You may also provide optional suggestions and comments to authors that they might find helpful in planning their study.

(Please upload your review as an attachment if it exceeds 20,000 characters)

Reviewer #1: The study protocol aims to address an important clinical question and the use of KCl in refractory VF is a novel treatment. The rationale and motivation of the study is well described in the introduction. I have a few detailed comments:
1. In the methodology section, the study setting is not specified.

2. In the sample size calculation, the outcome is not specified. what did you mean by "efficacy rate"? is that survival outcome? ideally, the sample size calculation should be based on the primary outcome. If this is a preliminary feasibility study, I suggest to change primary outcome to some more intermediate outcome, which requires less sample size.

**Answer: We agree and thank the reviewer for this remark. The efficacy rate is patient’s survival at hospital admission.**

3. The target population of the study is OHCA, but your participating centers are hospitals. How can you know which hospital will a patient be sent to when the OHCA is first witnessed.

**Answer: We thank the reviewer for this question. As specified in the manuscript, investigation centers are mobile intensive care unit of the 4 centers. The mobile intensive care unit does not always transport the patient to his own centre. A sentence was added in the revised version of the manuscript to clarify this point.**
4. The study design should be explicitly described. is that a pre-and post-implementation study. Or paralell cohort study?

**Answer: We thank and agree with the reviewer for this comment. The study design is 2 steps cohort design. A sentence was added in the revised version to clarify this point.**

Reviewer #2: First of all, this paper is the protocol of future study to evaluate the effectiveness of potassium chloride in the management of out-of-hospital cardiac arrest by refractory ventricular fibrillation.
The Reviewer felt that the current manuscript has somewhat value in terms of the detailed impact of potassium chloride in the management of out-of-hospital cardiac arrest. The Reviewer would like to raise some comments. However, the reviewer sincerely hope that those would be helpful for the authors.

λ Adjusting for sample size is the most important factor for the success of clinical studies. The authors described in this paper that it is necessary to include 81 patients to demonstrate an efficacy of 50% with a minimum efficacy of 35%. The reviewer wonders the sample number is too small for this study. The causes of OHCA will be multiple, although patients with presumed cardiac outpatient cardiac arrest are included.

**Answer: We thank and agree with reviewer with this interesting remark. However, the** Simon’s two-stage experimental design was chosen to avoid unnecessary inclusion and to optimize the number of patients to include in the second phase of the study. This design allows to define the optimal

λ How is the cause of OHCA presumed to be of cardiac origin?

**Answer: The presumed cardiac origin of OHCA is based on clinical history, anamnestic elements. A sentence was added in the revised version of the manuscript to clarify this.**

7. PLOS authors have the option to publish the peer review history of their article ([what does this mean?](https://journals.plos.org/plosone/s/editorial-and-peer-review-process#loc-peer-review-history)). If published, this will include your full peer review and any attached files.


If you choose “no”, your identity will remain anonymous but your review may still be made public.


**Do you want your identity to be public for this peer review?** For information about this choice, including consent withdrawal, please see our [Privacy Policy](https://www.plos.org/privacy-policy).

Reviewer #1: **Yes:** zhongheng zhang

Reviewer #2: No
